# Virtual Screening of Cablin Patchouli Herb as a Treatment for Heat Stress: A Study Based on Network Pharmacology, Molecular Docking, and Experimental Verification

**DOI:** 10.1155/2021/8057587

**Published:** 2021-03-10

**Authors:** Yan Xu, Lizhong Ding, Zhongtian Wang, Yanbo Wang, Liping Sun

**Affiliations:** ^1^Changchun University of Chinese Medicine, Changchun, 130117, China; ^2^Affiliated Hospital, Changchun University of Chinese Medicine, Changchun, Jilin 130021, China

## Abstract

Heat-related diseases have long been known to damage the structure and function of essential macromolecules such as proteins, lipids, and nucleic acids, thereby compromising the integrity of cells and tissues and the physiological functions of the entire organism. Heat stress is the physical discomfort caused by overheating the body and is also the initial manifestation of heat-related diseases. Cablin patchouli herb (CPB) has been used in China for two thousand years and has been used to treat heat stress, but to date, no related mechanistic research is available. In this study, KEGG and PPI networks and the TCMSP and GEO databases were used to explore the components of CPB in relation to heat stress: quercetin, genkwanin, irisolidone, 3,23-dihydroxy-12-oleanen-28-oic acid, and quercetin 7-O-*β*-D-glucoside. The targets identified were EGFR, NCOA1, FOS, HIF1A, NFKBIA, and NCOA2; these proteins were verified by molecular docking and experimental verification. In short, our research represents the first report on the use of the traditional Chinese medicine CPB to treat heat stress and thus has pioneering significance.

## 1. Introduction

As global warming intensifies, heat-related diseases have become a major public health issue during heat waves around the world, even in areas traditionally considered to be temperate and cold. In July 1995, a heat wave in the United States killed more than 700 people in less than 7 days, and more than 3,000 people visited the emergency room [1]. In 2003 and 2010, two severe heat waves affected Western Europe and Russia, respectively, causing 70,000 deaths and 55,000 deaths [2, 3]. More seriously, children account for almost half of the total population suffering from heat-related diseases (47.6%) [4].

Heat-related diseases have been known since ancient times; they reflect the relationship between the human body and heat imbalance [5], and long-term exposure to excessive heat or certain drugs that cause fever are the main drivers of heat-related diseases [6]. Heat can damage the structure and function of essential macromolecules such as proteins, lipids, and nucleic acids, thereby compromising the integrity of cells and tissues and the physiological functions of the entire organism, especially the central nervous system [7–10]. Heat-related conditions can be categorized into heat rash, heat cramps, heat stress, heat exhaustion, and heat stroke according to their severity; all these conditions have fever in common [4, 6]. In this study, we focused on exploring heat stress, an early manifestation of heat-related diseases. The symptoms of heat stress include prolonged fever, excess sweating or thirst, tiredness, and cramps; however, tachycardia and nervous system damage do not manifest.

Cablin patchouli herb (CPB) was first published in “Mingyi Bielu,” a medical book of the Han Dynasty, more than 2,000 years ago. It has the effect of “dispelling heat and dampness” and is recognized as a cure for heat stress. The Chinese patent medicine Huoxiang Zhengqi water, which uses CPB as its main medicinal material, was described in the textbook “Internal Medicine of Traditional Chinese Medicine” in China, and it is also considered to exhibit excellent effects in treating heat stress (yin-related heat-related diseases). It should be noted that according to the Chinese Yin-Yang Theory, the scope of heat-related diseases is broad and can be divided into Yin and Yang. Heat stress is usually biased towards yin-related heat-related diseases that is relatively less harmful to the human body. Heat exhaustion and heat stroke are generally regarded as yang-related heat-related diseases that are more harmful to the human body. CPB is usually used to treat heat stress (i.e., yin-related heat-related diseases). At present, reports on CPB treatment of heat stress are concentrated in the Chinese literature. Fusen used scraping therapy combined with Huoxiang Zhengqi water (for which the main component is CPB) to treat 48 cases of yin-related heat stroke and achieved good results [11]. Li used Huoxiang Zhengqi water to treat 240 cases of pediatric fever and found that children's heat-related symptoms were significantly reduced 3-4 hours after treatment [12]. Gu Fang and Rinan also used Huoxiang Zhengqi water to treat 18 cases of pediatric heat rash, a rash caused by heat. The effective rate for the treatment group reached 94.4%, which was significantly better than that of the control group [13]. Chaobin and Wanzhou elaborated on the theoretical basis of traditional Chinese medicine for the treatment of yin-related heat stroke with CPB. They believed that CPB can also treat fever and diarrhea [14]. There are some studies on the treatment of diarrhea [15, 16]. In short, since 2000, Chinese people have reliably used CPB to treat heat-related diseases, and its therapeutic effects are very good. However, with the strong development of science in the modern era, a great deal of interest has arisen in understanding such traditional disease treatment methods. It is necessary to study specific treatment mechanisms and to clarify their specific ingredients and targets.

However, there is a major difficulty in exploring the treatment mechanism of CPB: the complexity of Chinese medicine components and the multiplicity of pharmacological effects make it difficult to conduct comprehensive and systematic research in the context of Chinese medicine [17]. To solve this problem, after decades of research, Li Shao proposed in 1999 that there may be a connection between TCM syndrome and molecular networks [18–23]. Based on the rapid development of systems biology and computer technology, network pharmacology was born. Network pharmacology is based on the herb network-biological network-phenotype network interaction network [20]. Through network analysis, we can systematically observe the interventions and impacts of drugs on diseases and reveal the mysteries of drug molecules synergistically acting on the human body. This is the same as the core concept of traditional Chinese medicine: the principle of multicomponent, multipathway, and multitarget synergistic effects. Network pharmacology is also rapidly becoming a frontier research field in current drug research and next-generation drug research models [18].

Based on the above information, in this study, we used network pharmacology and molecular docking and experimental verification methods to explore the specific mechanisms by which CPB treats heat stress. Our goal was to enable people outside China to recognize and accept CPB and to help patients with heat-related diseases worldwide.

## 2. Materials and Methods

### 2.1. The Composition and Targets of CPB

The TCMSP database (http://tcmspw.com/tcmsp.php) [24] was used to identify the active ingredients and targets of CPB. Oral bioavailability (OB), drug-like properties (DL), relative molecular mass (MV), octanol-water partition coefficient (AlogP), number of H-bond coordination electron donors (Hdon), and number of H-bond coordination electron acceptors (Hacc) were used as indicators to compare the physical and chemical properties of the CPB components and to study the similarities and differences between the components.

### 2.2. Genes Differentially Expressed during Heat Stress

Differentially expressed genes were obtained from NCBI-GEO, a free public microarray/gene profile database. GEO accession number GSE90763 includes the transcriptome information of 15 volunteers who were subjected to heat stress. The data before exposure (T0) and at the end of heat exposure (T1) were selected for comparison. These data were analyzed using the GEO2R online tool, and 1,789 differentially expressed genes were identified using |logFC| ≥ 1 and *P* value < 0.05 as criteria.

### 2.3. Disease-Drug-Target Network

After the composition of CPB was obtained using OB > 30% and DL > 0.18, the targets of CPB components were downloaded from the TCMSP database, and the target elements were entered into the UniProt database one by one (https://www.UniProt.org/, currently the most complete nonredundant protein sequence database with the most complete sequence data and the richest annotation information in the world) for correction and conversion to GeneSymbol. Next, differentially expressed genes were used to intersect the corrected CPB target and were imported into Cytoscape (version: 3.6.1) and used to construct the CPB ingredient-target-disease network.

### 2.4. Molecular Docking

The protein data for P-gp (PDB ID : 4XWK), CYP3A4 (PDB ID : 3NXU), EGFR (PDB ID : 2ITW), FOS (PDB ID : 1A02), HIF1A (PDB ID : 1H2M), NCOA1 (PDB ID : 1K4W), NCOA2 (PDB ID : 2ZXZ), and NFKBIA (PDB ID : 6Y1J) were downloaded from the PDB database (http://www.rcsb.org/). Then, MGLTools 1.5.6 was used to process the protein data, simulate hydrogenation, calculate the charge, merge the nonpolar hydrogen, and save the results as a pdbqt file. According to each protein's ligand, the active site was defined, the grid box coordinates were set, and the box size was defined as 40 × 40 × 40 grid points; the distance between the small grid points was 0.1 nm. AutoDock Vina 1.1.2 was used to simulate the docking of molecules and proteins. The affinity was less than 0, indicating that the receptor and ligand can spontaneously bind.

### 2.5. PPI Network Enrichment and KEGG Analyses

The protein-protein interaction (PPI) network was analyzed using the Search Tool for the Retrieval of Interacting Genes (STRING, http://string.embl.de/) database (organism: *Homo sapiens)*. Kyoto Encyclopedia of Genes and Genomes (KEGG) pathway enrichment analyses were performed using the clusterProfiler package in R (3.6.1) software with pvaluecutoff = 0.05 and qvaluecutoff = 0.05 [25].

### 2.6. CPB Animal Experiment Verification

#### 2.6.1. Drugs and Materials

CPB was purchased from Jiangyin Tianjiang Pharmaceutical Co., Ltd. (production batch number: 19086254) and diluted to 10 mg/mL, 20 mg/mL, and 40 mg/mL with normal saline (NS) for use. Ibuprofen suspension (production batch number: H19991011) was purchased from Shanghai Johnson Pharmaceutical Co., Ltd. and was used at 0.6 g/kg and diluted with NS. The NCOA1 (MD696513) kit, RIPA lysis buffer (article number: MD912016), BCA protein concentration determination kit (article number: MD913053), SDS-PAGE precast gel kit (article number: MD911919), and Rabbit Anti-Mouse antibody (MD912566) were purchased from Beijing Biotek Biomedical Technology Co., Ltd. PVDF membrane (Millipore, USA, ISEQ00010) and medium protein molecular weight marker (26617) were purchased from Thermo, USA, and EGFR antibody (ab32077) and C-FOS antibody (ab134122) were purchased from Abcam, USA. SDS-PAGE system (U.S. BIO-Rad company, model Mini-PROTEAN), protein wet transfer instrument (U.S. BIO-Rad company, model Mini Trans-Blot), and western blot imaging system (U.S. BIO-Rad company, model 170-8280) were employed.

#### 2.6.2. Animals and Models

Forty-two specific pathogen-free (SPF) rats (female, 3-5 weeks old, 50-70 g) were purchased from Liaoning Changsheng Biotechnology Co., Ltd. (experimental animal production license number: NO.SCXK (Liao) 2020-0001) and subjected to the following conditions: rearing temperature—(24 ± 3)°C, relative humidity—(40 ± 5)%, illumination—alternating light and dark, noise <55Db, free water and food, and twice-weekly litter change. The animal experimentation was approved by the Animal Ethics Committee of Changchun University of Traditional Chinese Medicine (No. 20190116). The basal body temperature of SD rats was measured 3 days before the experiment, and the average value of the rectal temperature measured at three time points in the morning, midnight, and evening was used as the basal body temperature of the experimental animal. The rats were divided into 7 groups, 6 in each group, and fasted 6 hours before the experiment. Except for the blank group and the blank + NS group, the other 5 groups were subcutaneously injected with 10% dry yeast suspension (10 mL/kg) on the backs of rats. Body temperature was measured every 1 hour after modeling. After successful modeling (△*T* > 0.8°C, approximately 5 hours), each group was given intragastric administration: the blank + NS and model group were given 31 mL/kgNS, and the CPB treatment group and the ibuprofen group were given different concentrations of CPB and 31 mL/kg ibuprofen. After the administration, the body temperature was measured every 1 h, a total of 10 times, and the body temperature change value was calculated. Ten hours after the dry yeast group was modeled, a microcapillary was used to collect blood from the fundus venous plexus of the rats in each group. After standing at room temperature for 30 minutes, the blood samples were centrifuged at 4°C and 300 r/min for 10 minutes, the upper serum was drawn and stored in a −80°C refrigerator for use, and IL-1*β*, IL-6, TNF-*α*, and NCOA1 were detected in serum according to the instructions of the ELISA kit. After blood collection, the rats were anesthetized by intraperitoneal injection of 10% chloral hydrate (4 mL/kg). The skin was then cut along the costal arches on both sides, the abdominal cavity was opened, and curved scissors were used to create a small cut at the junction of the diaphragm and the sternum stem; this cut was then extended to both sides, the diaphragm and ribs were cut, the heart was fully exposed, and intravenous drip with a needle was used. The apex of the left ventricle was entered and fixed with a vascular clamp. NS (room temperature) was quickly instilled while cutting the right atrial appendage. When the blood color of the outflow liquid was light and almost clear, the perfusion was stopped. The brain was decapitated immediately, and the hypothalamus was removed from the center of the optic chiasm and gray nodules. The hypothalamus was quickly frozen in liquid nitrogen for 20 minutes and then stored at −80°C.

#### 2.6.3. Western Blot Detection

Each experimental group included an equal mass of hypothalamic samples. RIPA buffer was added to produce lysate, and each sample was shaken with a tissue homogenizer for 5 minutes, centrifuged at 12,000 r/min for 15 minutes to obtain the supernatant, and the protein concentration was measured by BCA. After mixing with the loading buffer, samples were boiled in a boiling water bath for 5 minutes, and SDS-PAGE was employed. The protein was transferred to PVDF membrane by the wet transfer method, and 5% skimmed milk powder was sealed at room temperature for 2 hours. The primary antibody was incubated overnight at 4°C. After washing the membrane with TBST, the secondary antibody was added to incubate at 37°C for 1 hour. After washing the membrane with TBST, the color developed.

### 2.7. Statistics

The data were expressed as the mean ± SD (*n* ≥ 3) and analyzed by GraphPad Prism 6.0 (GraphPad Software) using Student's *t*-test ( ^*∗*^*p* < 0.05 and ^*∗∗*^*p* < 0.01).

## 3. Results

### 3.1. General Description of CPB

The TCMSP database was used to collect CPB composition information and produce statistical descriptions based on the OB, DL, MV, AlogP, Hdon, and Hacc parameters, as shown in Table 1. The average value of OB was 37.90 (20.22), and the average value of DL was 0.20 (0.22), higher than the value range found by previous studies (OB > 30%, DL > 0.18) [26]. This shows that CPB has good oral availability and drug-like properties.

### 3.2. Further Screening of CPB Components

To eliminate possible false-positive components, OB > 30% and DL > 0.18 were also used as screening indices [26], and the CPB components were screened more stringently. As shown in Table 2, a total of 11 components satisfied the new screening conditions.

### 3.3. Eleven Active Ingredients Docked with P-gp and CYP3A4 Molecules

**Figure 1:**
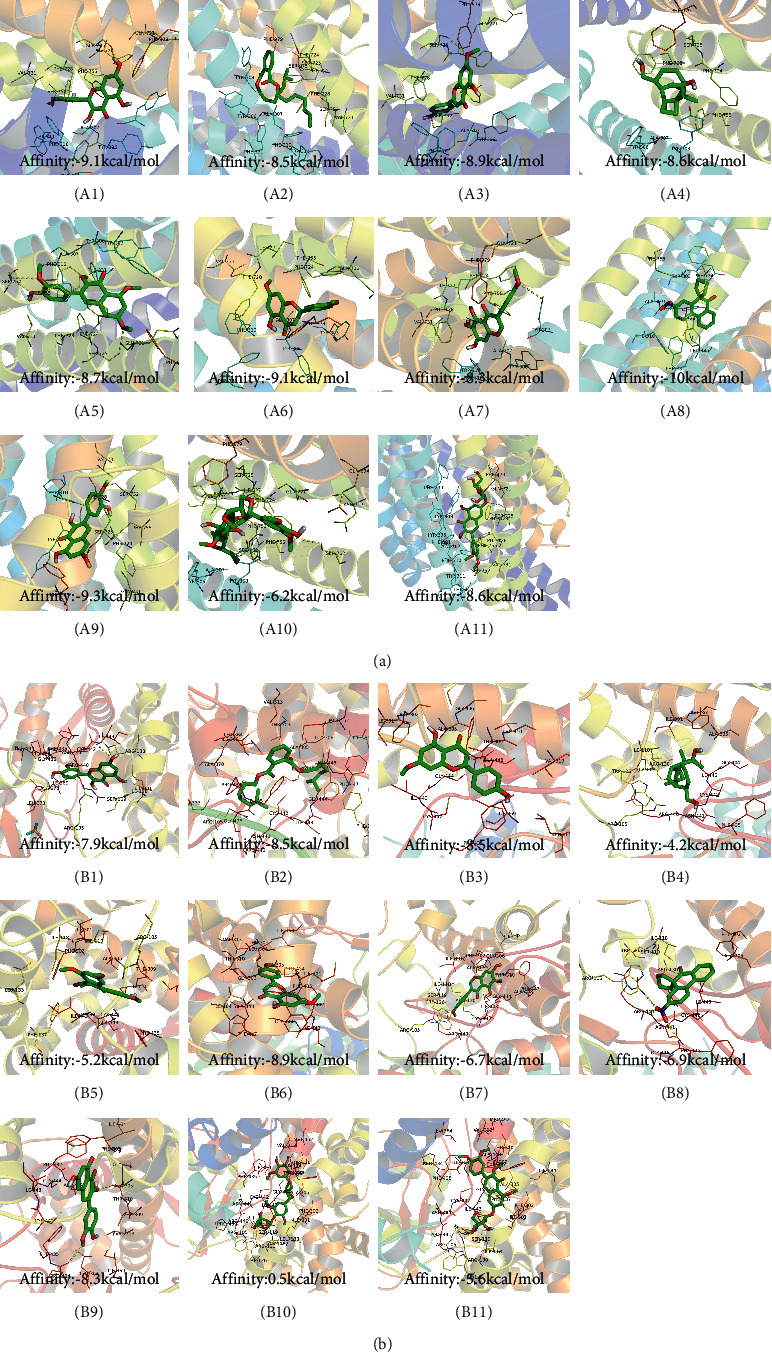
The 11 active components of CPB docked with P-gp and CYP3A4 molecules. (a) P-gp. (b) CYP3A4. 1, quercetin; 2, Diop; 3, genkwanin; 4; patchoulan 1,12-diol; 5, pachypodol; 6, 5-hydroxy-7,4′-dimethoxyflavanone; 7, irisolidone; 8, phenanthrone; 9, quercetin 7-O-*β*-D-glucoside; 10, acanthoside B; 11, 3,23-dihydroxy-12-oleanen-28-oic acid. *A*1, *A*3, *A*5, *A*7, *A*9, *A*10, *A*11, *B*1, *B*5, *B*7, *B*8, *B*9, *B*10, and *B*11 are hydrogen bond links and short-range van der Waals forces or *π* interaction forces. *A*2, *A*4, *A*6, *A*8, *B*2, *B*3, *B*4, and *B*6 are short-range van der Waals forces or *π* interaction force connections.

The absorption, distribution, metabolism, and excretion of CPB in the human body are affected by many factors. Among them, the interaction between P-gp and cytochrome CYP3A4 plays a key role [35]. P-gp is widely distributed and expressed in intestinal epithelial cells, hepatocytes, and capillary endothelial cells and plays an important role in the process by which most heterologous compounds pass through the blood-brain barrier and the blood-testis barrier [35, 36]. CYP3A4 is the most important metabolic enzyme in the P450 enzyme system and contributes to the metabolism of 1/3 of oral drugs [35, 37]. P-gp and CYP3A4 can synergistically restrict the entry of foreign substances into the human body in the intestine [35, 38]. To explore whether the active ingredients of CPB can enter the human body and play pharmacological roles, the molecular docking method was used for verification, as shown in Figure 1.

P-gp and CPB molecular docking showed that the 11 active ingredients could successfully dock and that their affinity values were less than 0. In addition, the docking results of CYP3A4 and CPB with the active ingredient molecules also showed that they could successfully dock, with the exception of acanthoside B. Given this result, acanthoside B was removed from the list of main active ingredients, and the subsequent steps examined only the remaining ingredients.

### 3.4. Drug-Ingredient-Target-Disease Network

**Figure 2:**
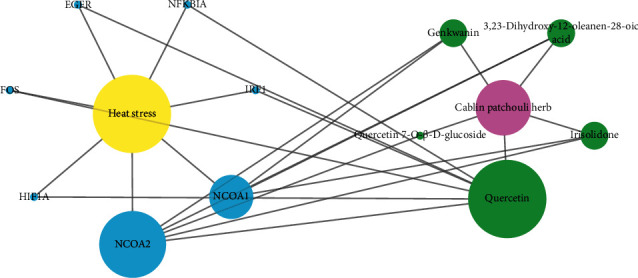
Drug-component-target-disease network. Blue represents targets, green represents drug molecules, magenta represents the drug, and yellow represents the disease. The size of each node represents its degree value: the larger the node is, the greater the degree value is.

Genes differentially expressed in heat stress were identified through the GEO database. Using |logFC|>1 and *P* value < 0.05 as criteria, a total of 1,789 differentially expressed genes were obtained (Supplementary Table 1). The CPB component targets were then downloaded from the TCMSP database, and UniProt database correction was performed to remove some targets that have not been manually annotated and reviewed. After correction, only 85 targets with 8 components (Supplementary Table 2) were obtained. Immediately afterwards, the differentially expressed genes of heat-related diseases and the corrected CPB targets were used for intersection, and a drug-component-target-disease network was generated, as shown in Figure 2. Only 5 molecules and their corresponding targets corresponded to heat stress genes. The molecules were quercetin, genkwanin, irisolidone, 3,23-dihydroxy-12-oleanen-28-oic acid, and quercetin 7-O-*β*-D-glucoside. Studies have shown that quercetin can increase secretion in cherry tomato fruit in a hot environment and can increase disease resistance [39]. It is worth mentioning that similar to quercetin, those molecules are also found in several other medicinal plants, such as kales, onions, berries, apples, red grapes, broccoli, and cherries as well as tea and red wine [40]. However, the content differs for each medicinal plant. In addition, existing technology cannot make specific judgments about complex chemical reactions that occur simultaneously in different molecules in plants. After a Chinese medicine enters the body, there are also complicated processes of absorption, distribution, metabolism, and excretion. Therefore, we hypothesize that although different Chinese medicines may have some of the same molecules, based on the complex characteristics of multiple components and multiple targets of traditional Chinese medicine, it is still believed that each traditional Chinese medicine, and even each molecule, corresponds with a distinct biological process in the body.

### 3.5. KEGG Enrichment Analysis

**Figure 3:**
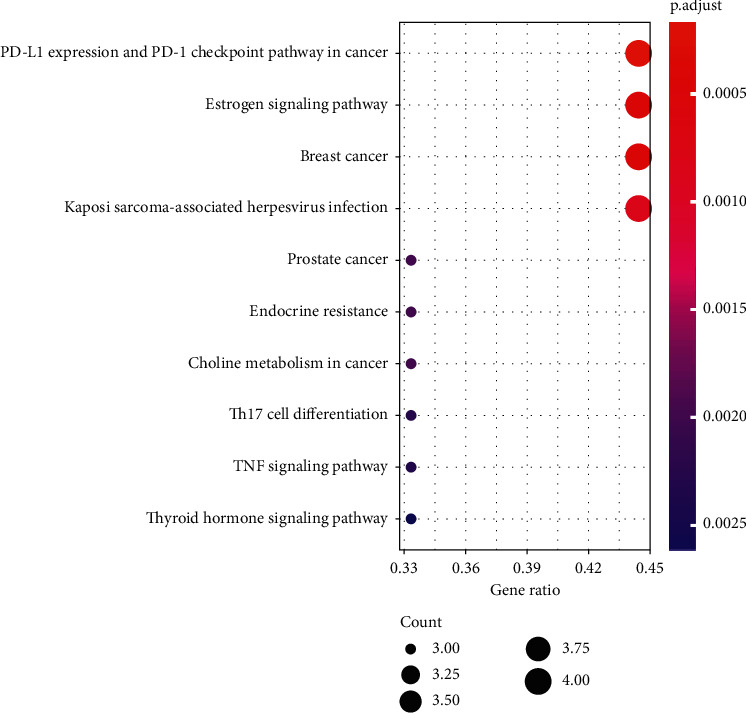
KEGG enrichment of CPB targets.

In the previous drug-component-target-disease network, we obtained 7 targets for possible treatments: EGFR, FOS, HIF1A, IRF1, NCOA1, NCOA2, and NFKBIA. However, the physiological and metabolic effects of CPB in the body were still unknown. Therefore, we performed KEGG enrichment analyses, as shown in Figure 3. Notably, in the KEGG enrichment analysis, we found that pathways related to PD-L1 expression and the PD-1 checkpoint pathway in cancer and estrogen signaling pathway were the most enriched.

The occurrence and development of tumors is accompanied by the formation of a special tumor immune microenvironment. Tumor cells can escape immune surveillance and destroy the host's immune checkpoints through various methods, thus avoiding elimination from the host's immune system [41]. Under normal physiological conditions, immune checkpoint molecules are in a balanced state such that the immune response of T cells maintains an appropriate intensity and scope in order to minimize damage to surrounding normal tissues and avoid autoimmune reactions [42]. However, cancer uses a variety of methods to upregulate negative signals through cell surface molecules, thereby inhibiting T cell activation or inducing apoptosis and promoting cancer progression and metastasis [43]. The immunotherapy method that uses antagonistic antibodies to block the immune checkpoint pathway can release cancer suppression and promote antitumor activity, thereby achieving the purpose of treating cancer. Programmed death 1 (PD-1) and its ligands PD-L1 (B7H1) and PD-L2 (B7-DC) represent the current main research directions with respect to immune checkpoint molecules [44]. The expression of PD-L1 is induced by a variety of proinflammatory factors (including type I and type II IFN-*γ*, IL-1*β*, TNF-*α*, LPS, and so on) [45, 46]. Proinflammatory factors are indispensable factors in the process of tumor formation and the main factors that cause fever in the human body. In conclusion, our research found that CPB may exert a certain regulatory effect on PD-L1 expression and the PD-1 checkpoint in cancer pathway.

Estrogen is a female steroid compound secreted by organs such as the ovaries and placenta. There are mainly three forms: estrone, estradiol, and estriol [47]. Previous studies have shown that estrogen can not only regulate reproductive behavior through estrogen receptors but also exert an important effect on the central nervous system [48]. Although the specific mechanism remains unclear, studies have shown that estrogen has a huge effect on body temperature regulation. The evidence includes the following: estrogen or related estrogen receptor ligand therapy can lower core temperature [49]; compared with ovariectomized rats that received estrogen, the temperature of skin vasodilation in ovariectomized rats was 4° lower [50]; the state of estrogen deficiency will increase the sensitivity of the thermal defense pathway, leading to the activation of heat loss effect factors at lower ambient temperatures [51].

### 3.6. PPI Network

**Figure 4:**
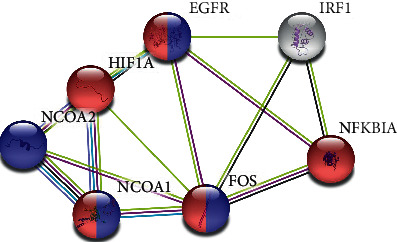
CPB target PPI network. Red indicates target proteins involved in cancer-related pathways; blue indicates target proteins involved in estrogen signaling.

As the agents of cell activity and function, proteins do not exist independently. The interactions between proteins play important roles in each stage of life and maintain the steady state of the internal environment. To analyze the interaction modes of the CPB target proteins and the targets of the two signaling pathways enriched by the KEGG analysis, the cancer and estrogen signaling pathways, a PPI network of the CPB target proteins was constructed, as shown in Figure 4.

Among them, EGFR, NCOA1, and FOS are involved in two pathways. Epidermal growth factor receptor (EGFR) is a transmembrane glycoprotein that is one of the four members of the ErbB family of tyrosine kinase receptors. The activation of EGFR leads to the autophosphorylation of receptor tyrosine kinase, which triggers a series of downstream signaling pathways and participates in the regulation of cell proliferation, differentiation, and survival. EGFR is abnormally activated through various mechanisms, such as receptor overexpression, mutation, ligand-dependent receptor dimerization, and ligand-independent activation, and is associated with the occurrence of various human cancers. EGFR inhibition is one of the key targets of cancer chemotherapy [52]. Nuclear receptor coactivators (NCOAs) are multifunctional transcriptional coregulators of a growing number of signal-activated transcription factors. The members of the p160 family (NCOA1/2) are increasingly recognized as essential and nonredundant players in a number of physiological processes [53]. This family has been shown to be related to cancer [54] and inflammation and metabolism [53]. FOS is a nuclear phosphoprotein encoded by mature mRNA transcribed from the c-fos gene and is closely related to seizures [55] and cancer [56]. Hypoxia-inducible factor 1-alpha (HIF1A) is an oxygen-dependent transcriptional activator that plays key roles in tumor angiogenesis and mammalian development [57]. Finally, NFKBIA encodes inhibitors of nuclear factor-*κ*B (NF-*κ*B) that regulate the translation of genes involved in inflammatory and immune reactions [58, 59]. In conclusion, our results demonstrate that EGFR, NCOA1, FOS, HIF1A, NFKBIA, and NCOA2 may be the main targets of CPB.

### 3.7. Molecular Docking Verification

**Figure 5:**
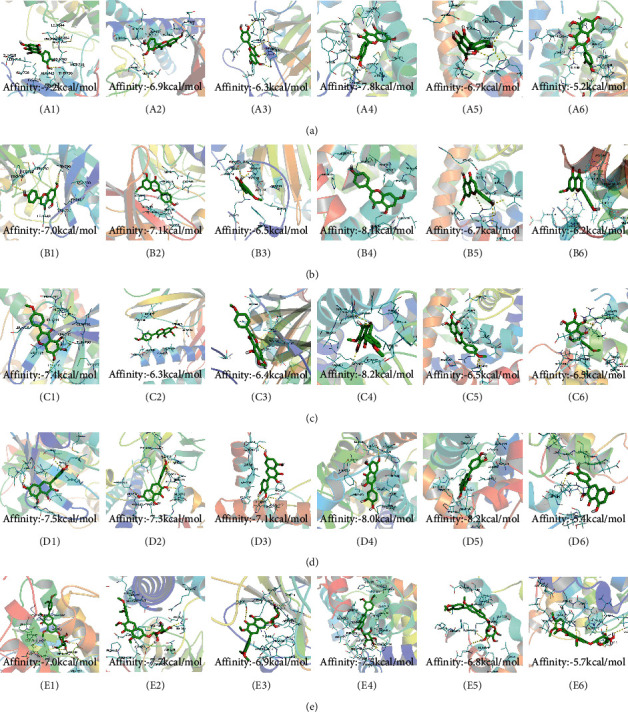
Docking results of CPB components and target molecules. (a) Quercetin. (b) Genkwanin. (c) Irisolidone. (d) Quercetin 7-O-*β*-D-glucoside. (e) 3,23-Dihydroxy-12-oleanen-28-oic acid. 1, EGFR; 2, FOS; 3, HIF1A; 4; NCOA1; 5, NCOA2; 6, NFKBIA. *A*1, *A*2, *A*3, *A*4, *A*5, *A*6, *B*1, *B*3, *B*5, *B*6, *C*1, *C*2, *C*3, *C*4, *C*5, *C*6, *D*1, *D*2, *D*3, *D*4, *D*6, *E*1, *E*2, *E*3, *E*4, *E*5, and *E*6 are hydrogen bond links and short-range van der Waals forces or *π* interaction forces. *B*2, *B*4, and *D*5 are short-range van der Waals forces or *π* interaction force connections.

In the above, we showed that quercetin, genkwanin, irisolidone, 3,23-dihydroxy-12-oleanen-28-oic acid, and quercetin 7-O-*β*-D-glucoside are the main components of CPB. EGFR, NCOA1, FOS, HIF1A, NFKBIA, and NCOA2 are the main target proteins, which we analyzed to more clearly verify whether the components and the targets can actually interact. We continued to use molecular docking technology for verification, and the results are shown in Figure 5. In short, the molecular docking verification of the 5 main components of CPB and the 6 main target proteins was successful.

### 3.8. Animal Experiment Verification

**Figure 6:**
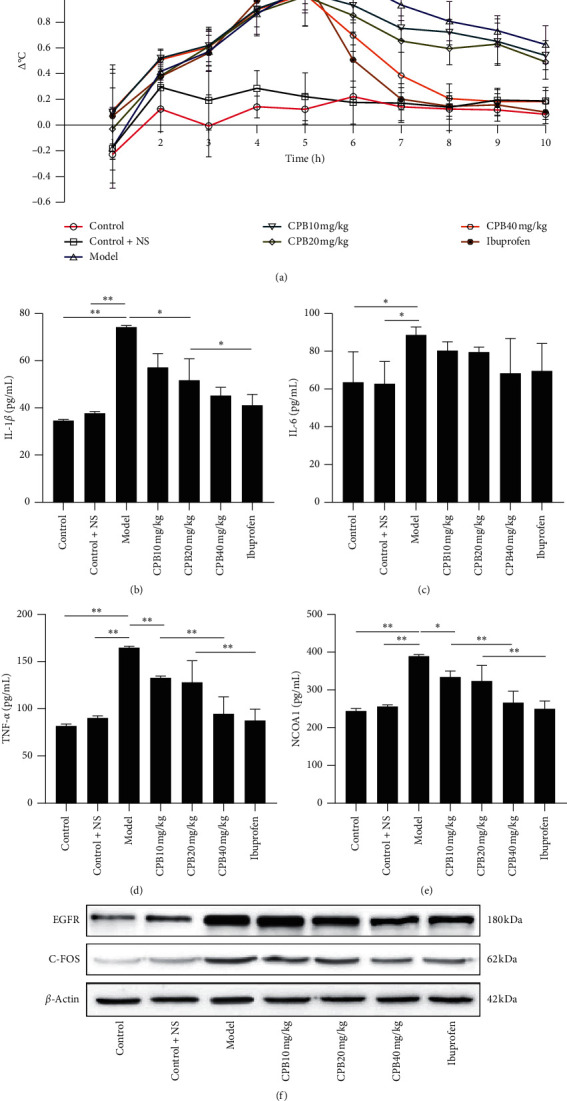
The effect of CPB on rats. (a) CPB on the body temperature of rats. (b) CPB on the amount of IL-1*β* in rat serum. (c) CPB on the amount of IL-6 in rat serum. (d) CPB on the amount of TNF-*α* in rat serum. (e) CPB on the amount of NCOAl in rat serum. (f) CPB on RGFR and C-FOS protein.

To further prove the availability of CPB, a rat model of fever was used to study CPB intragastric treatment. As shown in Figure 6(a), CPB at a concentration of 40 mg/kg showed a significant antipyretic effect after treatment. At the same time, we also measured the values of IL-1*β*, IL-6, TNF-*α*, and NCOA1 in the blood. As shown in Figures 6(b), 6(d), and 6(e), CPB can reduce the levels of IL-1*β*, TNF-*α*, and NCOA1 in rat serum. In Figure 6(c), the amount of IL-6 also seems to decrease, although it is not statistically significant. To further verify the conclusions we have reached, we used the protein expression in the rat hypothalamus to perform western blot verification. As shown in Figure 6(f), the expression of EGFR and C-FOS proteins in rats increased after modeling, but after CPB treatment, the expression of EGFR and C-FOS proteins decreased. In short, all animal experiments have successfully verified the conclusions we have reached.

## 4. Discussion

This study used network pharmacology combined with molecular docking. First, we collected the components and targets of CPB and screened them. Then, the ingredients were used for molecular docking with P-gp and CYP3A4 to initially verify that the active ingredients can be absorbed by the body. Then, we obtained genes which are differentially expressed during heat stress from the GEO database, constructed a drug-component-target-disease network, and further identified quercetin, genkwanin, irisolidone, 3,23-dihydroxy-12-oleanen-28-oic acid, and quercetin 7-O-*β*-D-glucoside as the main ingredients of CPB. We also constructed KEGG and PPI networks based on target information and proved that EGFR, NCOA1, FOS, HIF1A, NFKBIA, and NCOA2 are the main targets. Finally, the 5 active compounds were used to verify molecular docking with the 6 main targets.

**Figure 7:**
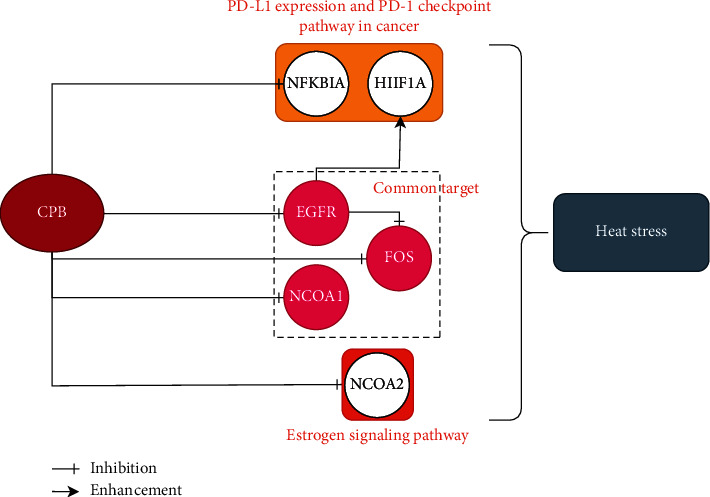
The target map of CPB for the treatment of heat stress.

To enhance the level of evidence for CPB in the treatment of heat stress, we continued to use febrile rats for experimental verification. We first proved that CPB can reduce the body temperature of febrile rats. The values of IL-1*β*, TNF-*α*, and NCOA1 in the blood of rats also decreased. Western blot detection also found that EGFR and C-FOS proteins in the hypothalamus were reduced. We have also produced a map of the targets of CPB, as shown in Figure 7.

In conclusion, our study shows that the main components of CPB as a treatment for heat stress are quercetin, genkwanin, irisolidone, 3,23-dihydroxy-12-oleanen-28-oic acid, and quercetin 7-O-*β*-D-glucoside. Its main targets are EGFR, NCOA1, FOS, HIF1A, NFKBIA, and NCOA2. Our study is the first network pharmacology study and experimental verification of the use of the traditional Chinese medicine CPB to treat heat stress; thus, this research has pioneering significance.

## Figures and Tables

**Table 1:** Physical and chemical properties of CPB components.

Index	Result
*n*	94
OB (mean (SD))	37.90 (20.22)
DL (mean (SD))	0.20 (0.22)
MW (mean (SD))	253.41 (118.93)
AlogP (mean (SD))	3.27 (1.77)
Hdon (mean (SD))	1.18 (2.12)
Hacc (mean (SD))	2.76 (3.82)

The values are expressed as the mean (SD).

**Table 2:** The 11 main components of CPB.

No.	MolID	MolName	OB	DL	Molecular structure	Reference
1	MOL002879	Diop	43.59	0.39	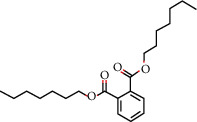	[27]
2	MOL005573	Genkwanin	37.13	0.24	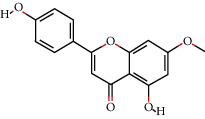	[27]
3	MOL005884	Patchoulan 1, 12-diol	38.17	0.25	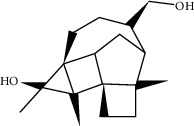	[27, 28]
4	MOL005890	Pachypodol	75.06	0.4	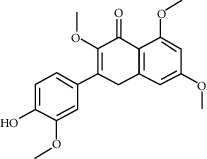	[29, 30]
5	MOL005911	5-Hydroxy-7, 4′-dimethoxyflavanone	51.54	0.27	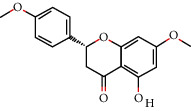	[31]
6	MOL005916	Irisolidone	37.78	0.3	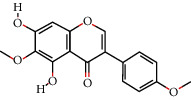	[27]
7	MOL005918	Phenanthrone	38.7	0.33	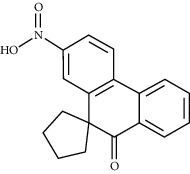	[27, 32]
8	MOL005921	Quercetin 7-O-β-D-glucoside	49.57	0.27	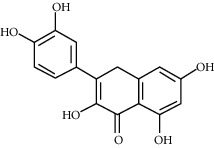	[31]
9	MOL005922	Acanthoside B	43.35	0.77	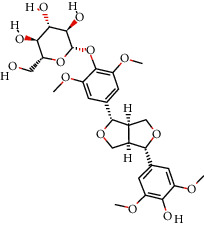	[27]
10	MOL005923	3, 23-Dihydroxy-12-oleanen-28-oic acid	30.86	0.86	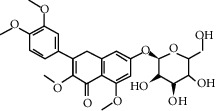	[27, 33]
11	MOL000098	Quercetin	46.43	0.28	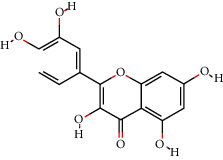	[31, 34]

## Data Availability

The research data used to support the findings of this study are included within the supplementary information files.

## Supplementary Materials

Supplementary Materials Supplementary Table 1: 1789 heat stress differentially expressed genes. Supplementary Table 2: the CPB components and targets after correction.
