# Supplementary material for: Virtual Screening of Cablin Patchouli Herb as a Treatment for Heat Stress: A Study Based on Network Pharmacology, Molecular Docking, and Experimental Verification
Source: Evid Based Complement Alternat Med. 2021 Mar 10;2021:8057587. doi: 10.1155/2021/8057587 (PMC7969090; doi:10.1155/2021/8057587)
Supplement: Supplementary Materials — Supplementary Table 1: 1789 heat stress differentially expressed genes. Supplementary Table 2: the CPB components and targets after correction. [file 8057587.f1.zip › 8057587.f1/Supplementary table 1.pdf]

ID	adj.P.Val	P.Value	logFC	Gene.symbol	Gene.title	Gene.ID
36829_at	0.0276	5.06E-07	-1.0241433	MIR6883///PER1	microRNA 6883///period circadian clock 1	102465532///5187
202861_at	0.0481	1.76E-06	-1.3599927	MIR6883///PER1	microRNA 6883///period circadian clock 1	102465532///5187
223507_at	0.0524	1.54E-05	1.0158453	CLPX	caseinolytic mitochondrial matrix peptidase chaperone subunit	10845
228173_at	0.0524	2.00E-05	-1.1601973	GNAS	GNAS complex locus	2778
214508_x_at	0.0524	2.05E-05	-1.2184273	CREM	cAMP responsive element modulator	1390
214982_at	0.0524	2.07E-05	-1.2221353	LOC101929240///SNRNP200	U5 small nuclear ribonucleoprotein 200 kDa helicase pseudogene///small nuclear ribonucleoprotein U5 subunit 200	101929240///23020

205239_at	0.0524	2.20E-05	-2.061636	AREG	amphiregulin	374
227160_s_at	0.0524	2.45E-05	-1.0008433	NDUFAF5	NADH:ubiquinone oxidoreductase complex assembly factor 5	79133
244677_at	0.0524	2.78E-05	-1.0866627	MIR6883///PER1	microRNA 6883///period circadian clock 1	102465532///5187
239616_at	0.0524	2.85E-05	1.1851993	REXO2	RNA exonuclease 2	25996
241762_at	0.0524	3.19E-05	-1.316064	FBXO32	F-box protein 32	114907
207630_s_at	0.0524	3.19E-05	-1.4566787	CREM	cAMP responsive element modulator	1390
1557278_s_at	0.0524	3.28E-05	-1.4739633	TNPO1	transportin 1	3842
214714_at	0.0524	3.47E-05	-1.253442	ZNF394	zinc finger protein 394	84124
231904_at	0.0524	3.50E-05	-1.310508	U2AF1	U2 small nuclear RNA auxiliary factor 1	7307
207078_at	0.0524	3.72E-05	-1.7349847	MED6	mediator complex subunit 6	10001

232931_at	0.0524	4.21E-05	-1.11469	SNRNP200	small nuclear ribonucleoprotein U5 subunit 200	23020
230229_at	0.0524	4.72E-05	-1.2755733	DLG1	discs large MAGUK scaffold protein 1	1739
232304_at	0.0524	4.81E-05	-1.6080533	PELI1	pellino E3 ubiquitin protein ligase 1	57162
233813_at	0.0524	5.69E-05	-1.3932873	PPP1R16B	protein phosphatase 1 regulatory subunit 16B	26051
1554309_at	0.0524	5.81E-05	-1.0469613	EIF4G3	eukaryotic translation initiation factor 4 gamma 3	8672
232213_at	0.0524	6.36E-05	-1.8652867	PELI1	pellino E3 ubiquitin protein ligase 1	57162
239042_at	0.0524	6.49E-05	-1.0579327	TSR1	TSR1, ribosome maturation factor	55720
213593_s_at	0.0524	6.50E-05	-1.452314	TRA2A	transformer 2 alpha homolog	29896

221942_s_at	0.0524	6.62E-05	1.0569013	GUCY1A3	guanylate cyclase 1 soluble subunit alpha	2982
225077_at	0.0524	7.00E-05	-1.078488	CHD2	chromodomain helicase DNA binding protein 2	1106
230742_at	0.0524	7.07E-05	-1.4283447	RBM5	RNA binding motif protein 5	10181
229193_at	0.0524	7.19E-05	-1.5700867	LUC7L3	LUC7 like 3 pre-mRNA splicing factor	51747
242549_at	0.0524	7.27E-05	-1.4112893	PRKD3	protein kinase D3	23683
1569320_at	0.0524	7.67E-05	-1.704848	GPBP1L1	GC-rich promoter binding protein 1 like 1	60313
241954_at	0.0524	7.85E-05	-1.2039233	FDFT1	farnesyl-diphosphate farnesyltransferase 1	2222
241905_at	0.0524	8.35E-05	-1.9841067	PIK3C2A	phosphatidylinositol-4-phosphate 3-kinase catalytic subunit type 2 alpha	5286
1559413_at	0.0524	8.64E-05	-1.350346	TCP11L2	t-complex 11 like 2	255394

230505_at	0.0524	9.02E-05	-1.4632667	LOC145474	uncharacterized LOC145474	145474
225768_at	0.0524	9.30E-05	-2.20912	NR1D2	nuclear receptor subfamily 1 group D member 2	9975
212240_s_at	0.0524	9.65E-05	-1.36726	PIK3R1	phosphoinositide-3-kinase regulatory subunit 1	5295
1556053_at	0.0524	9.71E-05	-1.23835	DNAJC7	DnaJ heat shock protein family (Hsp40) member C7	7266
1556007_s_at	0.0524	9.97E-05	-1.5810707	CSNK1A1	casein kinase 1 alpha 1	1452
239771_at	0.0524	1.00E-04	-1.2158773	CAND1	cullin associated and neddylation dissociated 1	55832
230885_at	0.0524	1.04E-04	-1.286692	LOC101930112///SPG7	uncharacterized LOC101930112///SPG7, paraplegin matrix AAA peptidase subunit	101930112///6687

214663_at	0.0524	1.04E-04	-1.189586	DSTYK	dual serine/threonine and tyrosine protein kinase	25778
1570415_at	0.0531	1.13E-04	-1.1884713	DDX52	DEAD-box helicase 52	11056
1560145_at	0.0531	1.19E-04	-1.4272613	MKLN1	muskelin 1	4289
1556277_a_at	0.0531	1.20E-04	-1.663182	PAPD4	poly(A) RNA polymerase D4, non-canonical	167153
236431_at	0.0531	1.23E-04	-1.1724007	U2SURP	U2 snRNP associated SURP domain containing	23350
232311_at	0.0531	1.26E-04	-1.3988127	B2M	beta-2-microglobulin	567
202757_at	0.0531	1.27E-04	-1.02485	NELFB	negative elongation factor complex member B	25920
1556323_at	0.0531	1.27E-04	-1.43535	CELF2	CUGBP, Elav-like family member 2	10659
242837_at	0.0531	1.30E-04	-1.8538967	SRSF4	serine and arginine rich splicing factor 4	6429

1559739_at	0.0531	1.36E-04	-1.540936	CHPT1	choline phosphotransferase 1	56994
63009_at	0.0531	1.37E-04	1.3652107	SHQ1	SHQ1, H/ACA ribonucleoprotein assembly factor	55164
226999_at	0.0531	1.40E-04	-1.037896	RNPC3	RNA binding region (RNP1, RRM) containing 3	55599
228962_at	0.0531	1.41E-04	-1.0358727	PDE4D	phosphodiesterase 4D	5144
224614_at	0.0531	1.44E-04	-1.000632	DYNC1LI2	dynein cytoplasmic 1 light intermediate chain 2	1783
229574_at	0.0531	1.45E-04	-1.4122507	TRA2A	transformer 2 alpha homolog	29896
215203_at	0.0531	1.53E-04	-1.39634	GOLGA4	golgin A4	2803
211833_s_at	0.0531	1.62E-04	-1.546598	BAX	BCL2 associated X, apoptosis regulator	581
219717_at	0.0531	1.67E-04	-1.1626487	DCAF16	DDB1 and CUL4 associated factor 16	54876

236754_at	0.0531	1.67E-04	-1.1226107	PPP1R2	protein phosphatase 1 regulatory inhibitor subunit 2	5504
228998_at	0.0531	1.75E-04	-1.187558	TNRC6B	trinucleotide repeat containing 6B	23112
232597_x_at	0.0531	1.77E-04	-1.127044	SCAF11	SR-related CTD associated factor 11	9169
201883_s_at	0.0531	1.82E-04	-1.4148047	B4GALT1	beta-1,4-galactosyltransferase 1	2683
232141_at	0.0531	1.84E-04	-1.5260607	U2AF1	U2 small nuclear RNA auxiliary factor 1	7307
215268_at	0.0531	1.90E-04	-1.2261087	KIAA0754	KIAA0754	643314
207492_at	0.0531	2.06E-04	-1.160164	NGLY1	N-glycanase 1	55768
1558088_a_at	0.0531	2.09E-04	-1.4907727	UBE2I	ubiquitin conjugating enzyme E2 I	7329
227817_at	0.0531	2.10E-04	-1.2640333	PRKCB	protein kinase C beta	5579
243683_at	0.0531	2.10E-04	-1.644622	MORF4L2	mortality factor 4 like 2	9643

230511_at	0.0531	2.12E-04	-1.1490933	CREM	cAMP responsive element modulator	1390
232441_at	0.0531	2.15E-04	-1.594792	KRR1	KRR1, small subunit processome component homolog	11103
1562255_at	0.0531	2.16E-04	-1.6221527	SYTL3	synaptotagmin like 3	94120
1563051_at	0.0531	2.21E-04	-1.392648	OSBP	oxysterol binding protein	5007
213089_at	0.0531	2.21E-04	-1.403306	LOC100272216	uncharacterized LOC100272216	100272216
235035_at	0.0531	2.21E-04	-1.0013473	SLC35E1	solute carrier family 35 member E1	79939
232371_at	0.0531	2.25E-04	-1.1086127	07-Mar	membrane associated ring-CH-type finger 7	64844
227891_s_at	0.0531	2.27E-04	-1.2684233	TAF15	TATA-box binding protein associated factor 15	8148
229718_at	0.0531	2.31E-04	-1.0917973	N4BP2L1	NEDD4 binding protein 2 like 1	90634
231911_at	0.0531	2.31E-04	-1.1182227	ERMN	ermin	57471

227223_at	0.0531	2.37E-04	-1.1077	RBM39	RNA binding motif protein 39	9584
236966_at	0.0531	2.38E-04	-1.1046593	ARMC8	armadillo repeat containing 8	25852
204610_s_at	0.0531	2.47E-04	-1.052198	CCDC85B	coiled-coil domain containing 85B	11007
236645_at	0.0531	2.52E-04	-1.6185353	HBP1	HMG-box transcription factor 1	26959
229765_at	0.0531	2.54E-04	-1.3594327	ZNF207	zinc finger protein 207	7756
243664_at	0.0531	2.56E-04	-1.362262	TXNL1	thioredoxin like 1	9352
1569450_at	0.0531	2.56E-04	-1.2243307	CAPZA2	capping actin protein of muscle Z-line alpha subunit 2	830
232722_at	0.0531	2.62E-04	-1.311108	RNASET2	ribonuclease T2	8635
202643_s_at	0.0531	2.62E-04	-1.369008	TNFAIP3	TNF alpha induced protein 3	7128
225170_at	0.0531	2.64E-04	-1.31864	WDR5	WD repeat domain 5	11091
201751_at	0.0531	2.67E-04	-1.133328	JOSD1	Josephin domain containing 1	9929

1554543_at	0.0544	2.75E-04	-1.316538	SPAG9	sperm associated antigen 9	9043
208478_s_at	0.0544	2.78E-04	-1.1530307	BAX	BCL2 associated X, apoptosis regulator	581
215599_at	0.0548	2.90E-04	-1.5096093	GUSBP9///GUSBP3	glucuronidase, beta pseudogene 9///glucuronidase, beta pseudogene 3	100049076///653188
201216_at	0.055	2.94E-04	-2.0987593	ERP29	endoplasmic reticulum protein 29	10961
232161_x_at	0.0553	2.97E-04	-1.1716187	PTPN4	protein tyrosine phosphatase, non-receptor type 4	5775
239948_at	0.0553	3.00E-04	-1.094846	NUP153	nucleoporin 153	9972
225995_x_at	0.0553	3.01E-04	-1.0273313	WASH1///WASH2P	WAS protein family homolog 1///WAS protein family homolog 2 pseudogene	100287171///375260

225549_at	0.0553	3.05E-04	-1.643618	DDX6	DEAD-box helicase 6	1656
227884_at	0.0553	3.06E-04	-1.2901967	TAF15	TATA-box binding protein associated factor 15	8148
396_f_at	0.0553	3.07E-04	1.007582	EPOR	erythropoietin receptor	2057
215210_s_at	0.0553	3.08E-04	-1.5170733	DLST	dihydrolipoamide S-succinyltransferase	1743
243751_at	0.0553	3.11E-04	-1.503986	CHD2	chromodomain helicase DNA binding protein 2	1106
233595_at	0.0557	3.20E-04	-1.3683553	USP34	ubiquitin specific peptidase 34	9736
211497_x_at	0.0558	3.23E-04	1.22004	NKX3-1	NK3 homeobox 1	4824
209739_s_at	0.0559	3.30E-04	1.038524	PNPLA4	patatin like phospholipase domain containing 4	8228
219235_s_at	0.0559	3.30E-04	-1.0964987	PHACTR4	phosphatase and actin regulator 4	65979

243514_at	0.056	3.38E-04	-1.3079667	WDFY2	WD repeat and FYVE domain containing 2	115825
1569126_at	0.056	3.41E-04	-1.1371027	CCNC	cyclin C	892
1560741_at	0.056	3.43E-04	-1.2639233	SNURF///SNRPN	SNRPN upstream reading frame///small nuclear ribonucleoprotein polypeptide N	8926///6638
204985_s_at	0.056	3.44E-04	-1.1691653	TRAPPC6A	trafficking protein particle complex 6A	79090
210799_at	0.056	3.44E-04	1.1375733	HTR1B	5-hydroxytryptamine receptor 1B	3351
209967_s_at	0.056	3.55E-04	-1.2595053	CREM	cAMP responsive element modulator	1390
215175_at	0.056	3.55E-04	-2.0356667	PCNX1	pecanex homolog 1 (Drosophila)	22990
228628_at	0.056	3.61E-04	-1.0902367	SRGAP2C	SLIT-ROBO Rho GTPase activating protein 2C	653464

213517_at	0.056	3.62E-04	-1.040004	PCBP2	poly(rC) binding protein 2	5094
208765_s_at	0.056	3.64E-04	-1.3665953	HNRNPR	heterogeneous nuclear ribonucleoprotein R	10236
1557207_s_at	0.0563	3.70E-04	1.0481933	LOC283177	uncharacterized LOC283177	283177
1557749_at	0.0563	3.73E-04	-1.2881673	EHBP1L1	EH domain binding protein 1 like 1	254102
1559975_at	0.0564	3.75E-04	-1.531442	BTG1	BTG anti-proliferation factor 1	694
225035_x_at	0.0566	3.90E-04	-1.5066453	LOC102723897///MIR6859-1///MIR6859-2///LOC101930154///LOC100288778///WASH1///WASH7P///WASH2P///WASH3P	WAS protein family homolog 2-like///microRNA 6859-1///microRNA 6859-2///WAS protein family homolog 6-like///WAS protein family homolog 1 pseudogene///WAS protein family homolog 1///WAS protein family	102723897///102466751///102465909///101930154///100288778///100287171///653635///375260///374666

223410_s_at	0.0566	3.91E-04	1.043498	FOXK2	forkhead box K2	3607
221755_at	0.0566	3.96E-04	-1.3158313	EHBP1L1	EH domain binding protein 1 like 1	254102
229966_at	0.0566	3.97E-04	-1.203022	EWSR1	EWS RNA binding protein 1	2130
218064_s_at	0.0566	4.02E-04	-1.1493133	AKAP8L	A-kinase anchoring protein 8 like	26993
1565717_s_at	0.0566	4.07E-04	-1.411228	FUS	FUS RNA binding protein	2521
210701_at	0.0566	4.13E-04	-1.3093887	CFDP1	craniofacial development protein 1	10428
229457_at	0.0566	4.27E-04	-1.225776	ANKHD1	ankyrin repeat and KH domain containing 1	54882
232323_s_at	0.0566	4.28E-04	-1.2405693	TTC17	tetratricopeptide repeat domain 17	55761
215213_at	0.0566	4.28E-04	-1.040752	NUP54	nucleoporin 54	53371
242020_s_at	0.0566	4.32E-04	-1.18441	ZBP1	Z-DNA binding protein 1	81030
1565483_at	0.0566	4.44E-04	2.527662	EGFR	epidermal growth factor receptor	1956
231440_at	0.0566	4.44E-04	1.042292	LINC00943	long intergenic non-protein coding RNA 943	100507206

211458_s_at	0.0566	4.46E-04	-1.9911653	GABARAPL3///GABARAPL1	GABA type A receptor associated protein like 3 pseudogene///GABA type A receptor associated protein like 1	23766///23710
228953_at	0.0566	4.50E-04	-1.087442	WHAMM	WAS protein homolog associated with actin, golgi membranes and microtubules	123720
217655_at	0.0566	4.66E-04	-1.205584	LOC100127972	uncharacterized LOC100127972	100127972
242903_at	0.0566	4.67E-04	-1.2081547	IFNGR1	interferon gamma receptor 1	3459
238079_at	0.0566	4.74E-04	-1.395356	TPM3	tropomyosin 3	7170
1558331_at	0.0566	4.78E-04	-1.071958	SIRT2	sirtuin 2	22933

1561937_x_at	0.0566	4.79E-04	1.2370727	IGHV4-31///IGHM///IGHG1///IGHA1	immunoglobulin heavy variable 4-31///immunoglobulin heavy constant mu///immunoglobulin heavy constant gamma 1 (G1m marker)///immunoglobulin heavy constant alpha 1	28396///3507///3500///3493
235213_at	0.0566	4.81E-04	-1.1330107	ITPKB	inositol-trisphosphate 3-kinase B	3707
1558136_s_at	0.0566	4.84E-04	-1.121234	TAF11	TATA-box binding protein associated factor 11	6882
211192_s_at	0.0566	4.86E-04	-1.0724447	CD84	CD84 molecule	8832
1562841_at	0.0566	4.87E-04	1.1238953	LOC339666	uncharacterized LOC339666	339666
223679_at	0.0566	4.94E-04	-1.3529613	CTNNB1	catenin beta 1	1499
208763_s_at	0.0566	5.01E-04	-1.2171713	TSC22D3	TSC22 domain family member 3	1831

226332_at	0.0566	5.10E-04	-1.5135227	FAM133DP///FAM133B	family with sequence similarity 133, member A pseudogene///family with sequence similarity 133 member B	728066///257415
215012_at	0.0566	5.11E-04	-1.2017907	ZNF451	zinc finger protein 451	26036
236241_at	0.0566	5.12E-04	-1.319214	MED31	mediator complex subunit 31	51003
232865_at	0.0566	5.12E-04	-1.3719447	AFF4	AF4/FMR2 family member 4	27125
225179_at	0.0566	5.19E-04	-1.266762	UBE2K	ubiquitin conjugating enzyme E2 K	3093
215201_at	0.0566	5.25E-04	-1.1058187	REPS1	RALBP1 associated Eps domain containing 1	85021
242946_at	0.0566	5.26E-04	-1.288834	CD53	CD53 molecule	963

213954_at	0.0566	5.26E-04	-1.1966493	FAM169A	family with sequence similarity 169 member A	26049
222045_s_at	0.0566	5.34E-04	-1.221848	PCIF1	PDX1 C-terminal inhibiting factor 1	63935
238534_at	0.0566	5.36E-04	-1.4740607	LRRFIP1	LRR binding FLII interacting protein 1	9208
214918_at	0.0566	5.41E-04	-1.739184	HNRNPM	heterogeneous nuclear ribonucleoprotein M	4670
225883_at	0.0566	5.48E-04	-1.585978	ATG16L2	autophagy related 16 like 2	89849
231182_at	0.0566	5.48E-04	-1.1160867	WIPF1	WAS/WASL interacting protein family member 1	7456
204197_s_at	0.0566	5.49E-04	-1.3990887	RUNX3	runt related transcription factor 3	864
220712_at	0.0566	5.50E-04	-1.004602	C8orf60	chromosome 8 open reading frame 60	619426
244801_at	0.0566	5.51E-04	-1.079358	PSMB7	proteasome subunit beta 7	5695

212239_at	0.0566	5.53E-04	-1.760332	PIK3R1	phosphoinositide-3-kinase regulatory subunit 1	5295
225137_at	0.0566	5.55E-04	-1.1576767	NFATC3	nuclear factor of activated T-cells 3	4775
233480_at	0.0566	5.56E-04	-1.0556247	TMEM43	transmembrane protein 43	79188
213637_at	0.0567	5.60E-04	-1.397272	DDX52	DEAD-box helicase 52	11056
217591_at	0.0568	5.72E-04	-1.3390367	SKIL	SKI-like proto-oncogene	6498
213015_at	0.0568	5.75E-04	-1.1219153	BBX	BBX, HMG-box containing	56987
216109_at	0.0568	5.83E-04	-1.5796407	MED13L	mediator complex subunit 13 like	23389
209953_s_at	0.0568	5.84E-04	-1.255368	CDC37	cell division cycle 37	11140
201641_at	0.0568	5.84E-04	-1.3563613	BST2	bone marrow stromal cell antigen 2	684

232991_at	0.0568	5.85E-04	-1.0631033	ARL17B///ARL17A	ADP ribosylation factor like GTPase 17B///ADP ribosylation factor like GTPase 17A	100506084///51326
238987_at	0.0568	5.88E-04	-1.4817833	B4GALT1	beta-1,4-galactosyltransferase 1	2683
236930_at	0.0568	5.95E-04	-1.203542	LOC101930388///LOC101928143///NUMB	uncharacterized LOC101930388///uncharacterized LOC101928143///NUMB, endocytic adaptor protein	101930388///101928143///8650
1560278_at	0.0568	6.00E-04	1.4034573	LOC221122	uncharacterized LOC221122	221122
91703_at	0.0568	6.00E-04	-1.0744227	EHBP1L1	EH domain binding protein 1 like 1	254102
228382_at	0.0569	6.09E-04	1.0575033	OTULIN	OTU deubiquitinase with linear linkage specificity	90268

1559039_at	0.0569	6.13E-04	-1.2141727	DHX36	DEAH-box helicase 36	170506
211930_at	0.0569	6.22E-04	-1.085824	HNRNPA3	heterogeneous nuclear ribonucleoprotein A3	220988
223584_s_at	0.057	6.26E-04	-1.170846	KBTBD2	kelch repeat and BTB domain containing 2	25948
226970_at	0.057	6.47E-04	-1.2030027	FBXO33	F-box protein 33	254170
1557384_at	0.057	6.51E-04	-1.184342	LOC100506639///ZNF131	uncharacterized LOC100506639///zinc finger protein 131	100506639///7690
229422_at	0.057	6.61E-04	-1.3976973	NRDC	nardilysin convertase	4898
208674_x_at	0.057	6.63E-04	-1.115004	DDOST	dolichyl-diphosphooligosaccharide--protein glycosyltransferase non-catalytic subunit	1650

242712_x_at	0.057	6.66E-04	-1.226448	RGPD2///RGPD6///RGPD8///RGPD3///RGPD1///RGPD4///RGPD5///RANBP2	RANBP2-like and GRIP domain containing 2///RANBP2-like and GRIP domain containing 6///RANBP2-like and GRIP domain containing 8///RANBP2-like and GRIP domain containing 3///RANBP2-like and GRIP domain containing 1///RANBP2-like and GRIP	729857///729540///727851///653489///400966///285190///84220///5903
1567277_at	0.057	6.69E-04	1.1807367	CTTN	cortactin	2017

1552622_s_at	0.057	6.77E-04	-1.4715687	UPK3BL///POLR2J3///LOC441259///POLR2J2///POLR2J	uroplakin 3B-like///RNA polymerase II subunit J3///PMS1 homolog 2, mismatch repair system component pseudogene///RNA polymerase II subunit J2///RNA polymerase II subunit J	100134938///548644///441259///246721///5439
221919_at	0.057	6.82E-04	-1.0484867	HNRNPA1	heterogeneous nuclear ribonucleoprotein A1	3178
223129_x_at	0.057	6.83E-04	-1.071026	MYLIP	myosin regulatory light chain interacting protein	29116
231848_x_at	0.057	6.85E-04	-1.139274	ZNF207	zinc finger protein 207	7756

228098_s_at	0.057	6.88E-04	-1.383612	MYLIP	myosin regulatory light chain interacting protein	29116
201055_s_at	0.057	6.89E-04	-1.021002	HNRNPA0	heterogeneous nuclear ribonucleoprotein A0	10949
220370_s_at	0.057	6.93E-04	-1.0905533	USP36	ubiquitin specific peptidase 36	57602
233914_s_at	0.057	6.94E-04	-1.2207233	SBF2	SET binding factor 2	81846
221959_at	0.057	7.02E-04	1.0175513	FAM110B	family with sequence similarity 110 member B	90362
1559126_at	0.057	7.04E-04	-1.3192787	RRP12	ribosomal RNA processing 12 homolog	23223
1556601_a_at	0.057	7.05E-04	-1.1980267	SPATA13	spermatogenesis associated 13	221178
225493_at	0.057	7.07E-04	-1.1117693	CCNT1	cyclin T1	904
230000_at	0.0571	7.19E-04	-1.1721553	RNF213	ring finger protein 213	57674
225262_at	0.0571	7.27E-04	-1.96296	FOSL2	FOS like 2, AP-1 transcription factor subunit	2355

239432_at	0.0571	7.28E-04	-1.2361313	PSMA3-AS1	PSMA3 antisense RNA 1	379025
244103_at	0.0571	7.28E-04	-1.3417287	SDE2	SDE2 telomere maintenance homolog	163859
240452_at	0.0575	7.36E-04	-1.0641407	GSPT1	G1 to S phase transition 1	2935
236621_at	0.0576	7.38E-04	-1.4415333	RPS27	ribosomal protein S27	6232
206965_at	0.058	7.62E-04	-1.124816	KLF12	Kruppel like factor 12	11278
242268_at	0.058	7.64E-04	-1.7171827	CELF2	CUGBP, Elav-like family member 2	10659
221267_s_at	0.058	7.66E-04	-1.0300153	ABHD17A	abhydrolase domain containing 17A	81926
36545_s_at	0.0585	7.86E-04	-1.1464293	SFI1	SFI1 centrin binding protein	9814
1565703_at	0.0588	7.95E-04	-1.1035247	SMAD4	SMAD family member 4	4089
242146_at	0.0589	7.97E-04	-1.2740693	SNRPA1	small nuclear ribonucleoprotein polypeptide A'	6627
1559127_x_at	0.0592	8.15E-04	-1.015498	RRP12	ribosomal RNA processing 12 homolog	23223

231403_at	0.0592	8.18E-04	-1.152434	TRIO	trio Rho guanine nucleotide exchange factor	7204
215567_at	0.0592	8.20E-04	-1.1297127	FCF1	FCF1 rRNA-processing protein	51077
60815_at	0.0592	8.20E-04	-1.002506	POLR2J4	RNA polymerase II subunit J4, pseudogene	84820
232216_at	0.0592	8.24E-04	-1.4186047	YME1L1	YME1 like 1 ATPase	10730
242492_at	0.0592	8.27E-04	-1.0251453	CLNS1A	chloride nucleotide-sensitive channel 1A	1207
242814_at	0.0592	8.28E-04	-1.140826	SERPINB9	serpin family B member 9	5272
214060_at	0.0596	8.33E-04	-1.0675013	SSBP1	single stranded DNA binding protein 1	6742
239188_at	0.0598	8.44E-04	-1.20576	PPP2R3C	protein phosphatase 2 regulatory subunit B''gamma	55012
239937_at	0.0598	8.48E-04	-1.1330467	ZNF207	zinc finger protein 207	7756

207535_s_at	0.0598	8.49E-04	-1.100824	NFKB2	nuclear factor kappa B subunit 2	4791
226663_at	0.0598	8.51E-04	-1.737706	ANKRD10-IT1	ANKRD10 intronic transcript 1	100505494
1554670_at	0.0599	8.55E-04	-1.25083	GGA1	golgi associated, gamma adaptin ear containing, ARF binding protein 1	26088
225239_at	0.0603	8.65E-04	-2.158112	MIR612///NEAT1	microRNA 612///nuclear paraspeckle assembly transcript 1 (non-protein coding)	693197///283131
208564_at	0.0603	8.65E-04	1.0790687	KCNA2	potassium voltage-gated channel subfamily A member 2	3737
224739_at	0.0603	8.65E-04	-1.1235087	PIM3	Pim-3 proto-oncogene, serine/threonine kinase	415116

233229_at	0.0606	8.85E-04	-1.1280753	SCFD1	sec1 family domain containing 1	23256
225405_at	0.0606	8.86E-04	-1.424336	HNRNPUL2	heterogeneous nuclear ribonucleoprotein U like 2	221092
209184_s_at	0.0606	8.93E-04	-1.115626	IRS2	insulin receptor substrate 2	8660
1569142_at	0.0606	9.05E-04	-1.1936287	TRIM13	tripartite motif containing 13	10206
1557360_at	0.0606	9.06E-04	-1.4084987	LRPPRC	leucine rich pentatricopeptide repeat containing	10128
1555241_at	0.0607	9.10E-04	-1.1451007	C8orf59	chromosome 8 open reading frame 59	401466
224852_at	0.0607	9.17E-04	-1.151948	TTC17	tetratricopeptide repeat domain 17	55761
201917_s_at	0.0607	9.18E-04	-1.2279107	SLC25A36	solute carrier family 25 member 36	55186

224566_at	0.0608	9.26E-04	-1.539016	MIR612///NEAT1	microRNA 612///nuclear paraspeckle assembly transcript 1 (non-protein coding)	693197///283131
210093_s_at	0.0609	9.32E-04	-1.5428573	MAGOHB///MAGOH	mago homolog B, exon junction complex core component///mago homolog, exon junction complex core component	55110///4116
225738_at	0.061	9.37E-04	-1.5220307	RAPGEF1	Rap guanine nucleotide exchange factor 1	2889
242261_at	0.0611	9.44E-04	-1.3914253	IREB2	iron responsive element binding protein 2	3658
242243_at	0.0612	9.61E-04	-1.163092	TMF1	TATA element modulatory factor 1	7110
244546_at	0.0616	9.73E-04	-1.51703	CYCS	cytochrome c, somatic	54205

208632_at	0.0616	9.77E-04	-1.200784	RNF10	ring finger protein 10	9921
1556914_at	0.0617	9.81E-04	1.2392033	LOC100652911	uncharacterized LOC100652911	100652911
204188_s_at	0.0623	1.00E-03	1.001108	RARG	retinoic acid receptor gamma	5916
1568780_at	0.0623	1.01E-03	-1.2310627	LOC729732	uncharacterized LOC729732	729732
239243_at	0.0623	1.01E-03	-1.5912727	ZNF638-IT1///ZNF638	ZNF638 intronic transcript 1///zinc finger protein 638	100507113///27332
1556202_at	0.0626	1.03E-03	-1.0413513	SRGAP2	SLIT-ROBO Rho GTPase activating protein 2	23380

233929_x_at	0.0626	1.03E-03	-1.4721613	MIR6859-1///MIR6859-2///LOC101930154///LOC100288778///WASH1///WASH7P///WASH2P///WASH3P	microRNA 6859-1///microRNA 6859-2///WAS protein family homolog 6-like///WAS protein family homolog 1 pseudogene///WAS protein family homolog 1///WAS protein family homolog 7 pseudogene///WAS protein family	102466751///102465909///101930154///100288778///100287171///653635///375260///374666
210240_s_at	0.0626	1.04E-03	-1.3484053	CDKN2D	cyclin dependent kinase inhibitor 2D	1032
1559826_a_at	0.0627	1.04E-03	1.2261807	LINC00960	long intergenic non-protein coding RNA 960	401074
217100_s_at	0.0627	1.05E-03	-1.2939367	UBXN7	UBX domain protein 7	26043
243006_at	0.0627	1.07E-03	-1.4994627	FYN	FYN proto-oncogene, Src family tyrosine kinase	2534
244804_at	0.0629	1.09E-03	-1.2310213	SQSTM1	sequestosome 1	8878

1570571_at	0.0629	1.09E-03	-1.5532787	CCDC91	coiled-coil domain containing 91	55297
223797_at	0.0629	1.09E-03	-1.0437693	LOC114224	uncharacterized LOC114224	114224
225551_at	0.0629	1.10E-03	-1.028406	CNST	consortin, connexin sorting protein	163882
227373_at	0.0629	1.10E-03	-1.632136	ATXN1L	ataxin 1 like	342371
243496_at	0.0629	1.10E-03	-1.149754	RAB18	RAB18, member RAS oncogene family	22931
1560112_at	0.0632	1.12E-03	-1.5185413	WDFY2	WD repeat and FYVE domain containing 2	115825
229106_at	0.0637	1.14E-03	-1.0507527	DYNLL2	dynein light chain LC8-type 2	140735
232365_at	0.0639	1.16E-03	-1.3538033	SIAH1	siah E3 ubiquitin protein ligase 1	6477
231864_at	0.0639	1.17E-03	-1.0738787	ZNF33A	zinc finger protein 33A	7581
200959_at	0.0639	1.17E-03	-1.5116667	FUS	FUS RNA binding protein	2521
224718_at	0.064	1.17E-03	-1.3829207	YY1	YY1 transcription factor	7528

222792_s_at	0.0641	1.18E-03	-1.0820653	CCDC59	coiled-coil domain containing 59	29080
223538_at	0.0641	1.18E-03	-1.049004	SERF1B///SERF1A	small EDRK-rich factor 1B///small EDRK-rich factor 1A	728492///8293
202887_s_at	0.0641	1.18E-03	-1.69538	DDIT4	DNA damage inducible transcript 4	54541
217957_at	0.0641	1.19E-03	-1.296496	CFAP20	cilia and flagella associated protein 20	29105
201101_s_at	0.0642	1.19E-03	-1.2109327	BCLAF1	BCL2 associated transcription factor 1	9774
228483_s_at	0.0647	1.21E-03	-1.0077247	TAF9B	TATA-box binding protein associated factor 9b	51616
201513_at	0.065	1.23E-03	-1.0453333	TSN	translin	7247
229694_at	0.0651	1.24E-03	-1.140132	WDR11	WD repeat domain 11	55717
214016_s_at	0.0652	1.24E-03	-1.119098	SFPQ	splicing factor proline and glutamine rich	6421

1559399_s_at	0.0653	1.26E-03	-1.220034	ZCCHC10	zinc finger CCHC-type containing 10	54819
240221_at	0.0653	1.28E-03	-1.1064933	CSNK1A1	casein kinase 1 alpha 1	1452
209006_s_at	0.0653	1.28E-03	-1.6813447	RSRP1	arginine and serine rich protein 1	57035
223746_at	0.0654	1.29E-03	-1.0928593	STK4	serine/threonine kinase 4	6789
244777_at	0.0654	1.30E-03	-1.351614	DCP2	decapping mRNA 2	167227
237919_at	0.0655	1.30E-03	1.1435833	RFFL	ring finger and FYVE-like domain containing E3 ubiquitin protein ligase	117584
202885_s_at	0.0655	1.31E-03	1.0671147	PPP2R1B	protein phosphatase 2 scaffold subunit Abeta	5519

213704_at	0.0655	1.31E-03	-1.0609667	SNORD45C///SNORD45A///SNORD45B///RABGGTB	small nucleolar RNA, C/D box 45C///small nucleolar RNA, C/D box 45A///small nucleolar RNA, C/D box 45B///Rab geranylgeranyltransferase beta subunit	692085///26805///26804///5876
239757_at	0.0656	1.33E-03	-1.063874	ZFAND6	zinc finger AN1-type containing 6	54469
234756_at	0.0658	1.35E-03	1.3353167	CACNG8	calcium voltage-gated channel auxiliary subunit gamma 8	59283

222669_s_at	0.0658	1.35E-03	-1.144466	SBDSP1///SBDS	Shwachman-Bodian-Diamond syndrome pseudogene 1///SBDS ribosome assembly guanine nucleotide exchange factor	155370///51119
1559993_at	0.0659	1.35E-03	-1.434776	SFXN3	sideroflexin 3	81855
1559964_at	0.0659	1.36E-03	-1.1222673	LOC401261	uncharacterized LOC401261	401261
243797_at	0.0659	1.36E-03	-1.1329547	STK17B	serine/threonine kinase 17b	9262
209657_s_at	0.0659	1.36E-03	-1.0210507	HSF2	heat shock transcription factor 2	3298
231108_at	0.0659	1.37E-03	-1.123588	FUS	FUS RNA binding protein	2521
225379_at	0.0659	1.38E-03	1.0074167	MAPT	microtubule associated protein tau	4137
36711_at	0.0659	1.38E-03	-1.9550467	MAFF	MAF bZIP transcription factor F	23764

202477_s_at	0.0659	1.38E-03	-1.1390373	TUBGCP2	tubulin gamma complex associated protein 2	10844
207073_at	0.0663	1.39E-03	1.0671787	CDKL2	cyclin dependent kinase like 2	8999
200815_s_at	0.0663	1.40E-03	-1.0667273	PAFAH1B1	platelet activating factor acetylhydrolase 1b regulatory subunit 1	5048
1558802_at	0.0665	1.42E-03	-1.0825507	TM2D1	TM2 domain containing 1	83941
221821_s_at	0.0665	1.42E-03	-1.1527313	KANSL2	KAT8 regulatory NSL complex subunit 2	54934
200810_s_at	0.0665	1.43E-03	-1.1712127	CIRBP	cold inducible RNA binding protein	1153

1560662_s_at	0.0665	1.44E-03	-1.1354253	WHAMMP2///WHAMMP3	WAS protein homolog associated with actin, golgi membranes and microtubules pseudogene 2///WAS protein homolog associated with actin, golgi membranes and microtubules pseudogene 3	440253///339005
220319_s_at	0.0665	1.48E-03	-1.176354	MYLIP	myosin regulatory light chain interacting protein	29116
227055_at	0.0665	1.53E-03	1.1523153	METTL7B	methyltransferase like 7B	196410

1564277_a_at	0.0665	1.53E-03	1.4309233	LOC101929583///LOC101928195///LOC100996643///LOC100133920///LOC286297	methylenetetrahydrofolate dehydrogenase (NADP+ dependent) 1-like pseudogene///methylenetetrahydrofolate dehydrogenase (NADP+ dependent) 1-like pseudogene///monofunctional C1-tetrahydrofolate synthase, mitochondrial-like///met	101929583///101928195///100996643///100133920///286297
202180_s_at	0.0665	1.53E-03	-1.0964373	MVP	major vault protein	9961
228284_at	0.0665	1.53E-03	-1.2079533	TLE1	transducin like enhancer of split 1	7088
235122_at	0.0665	1.54E-03	-1.0280827	HIVEP3	human immunodeficiency virus type I enhancer binding protein 3	59269
209545_s_at	0.0665	1.54E-03	-1.0208193	RIPK2	receptor interacting serine/threonine kinase 2	8767

224368_s_at	0.0665	1.55E-03	-1.2499447	NDRG3	NDRG family member 3	57446
222310_at	0.0665	1.55E-03	-1.1514807	SCAF4	SR-related CTD associated factor 4	57466
1556006_s_at	0.0665	1.56E-03	-1.410346	CSNK1A1	casein kinase 1 alpha 1	1452
230375_at	0.0665	1.56E-03	-1.1494607	PNISR	PNN interacting serine and arginine rich protein	25957
242349_at	0.0665	1.57E-03	-1.1480273	HECTD1	HECT domain E3 ubiquitin protein ligase 1	25831
208707_at	0.0665	1.58E-03	-1.091814	EIF5	eukaryotic translation initiation factor 5	1983
230998_at	0.0665	1.58E-03	-1.0162567	CBX3	chromobox 3	11335
1553703_at	0.0665	1.59E-03	-1.0920393	ZNF791	zinc finger protein 791	163049
1553349_at	0.0665	1.60E-03	-1.0671593	ARID2	AT-rich interaction domain 2	196528

222034_at	0.0665	1.60E-03	-1.0899447	SNORD96A///SNORD95///RACK1	small nucleolar RNA, C/D box 96A///small nucleolar RNA, C/D box 95///receptor for activated C kinase 1	619571///619570///10399
205596_s_at	0.0665	1.61E-03	-1.2413247	SMURF2	SMAD specific E3 ubiquitin protein ligase 2	64750
71933_at	0.0666	1.61E-03	1.0127847	WNT6	Wnt family member 6	7475
226840_at	0.0668	1.63E-03	-1.1513587	H2AFY	H2A histone family member Y	9555
1553878_at	0.0668	1.64E-03	1.6738333	GOT1L1	glutamic-oxaloacetic transaminase 1-like 1	137362
226620_x_at	0.0668	1.64E-03	-1.19838	DAZAP1	DAZ associated protein 1	26528
1569110_x_at	0.0669	1.65E-03	-1.4342847	LOC728613	programmed cell death 6 pseudogene	728613

226340_x_at	0.0679	1.71E-03	-1.2258247	MIR6859-1///MIR6859-2///LOC101930154///LOC100288778///WASH1///WASH7P///WASH2P///WASH3P	microRNA 6859-1///microRNA 6859-2///WAS protein family homolog 6-like///WAS protein family homolog 1 pseudogene///WAS protein family homolog 1///WAS protein family homolog 7 pseudogene///WAS protein family	102466751///102465909///101930154///100288778///100287171///653635///375260///374666
226732_at	0.0679	1.72E-03	-1.501026	RBM33	RNA binding motif protein 33	155435
218733_at	0.0679	1.73E-03	-1.36241	MSL2	male-specific lethal 2 homolog (Drosophila)	55167
207339_s_at	0.0679	1.73E-03	-1.0492073	LTB	lymphotoxin beta	4050
1553663_a_at	0.0679	1.73E-03	1.1976087	NPB	neuropeptide B	256933
239533_at	0.0679	1.73E-03	-1.211504	GPR155	G protein-coupled receptor 155	151556

240383_at	0.068	1.75E-03	-1.9274067	UBE2D3	ubiquitin conjugating enzyme E2 D3	7323
202880_s_at	0.068	1.75E-03	-1.0880993	CYTH1	cytohesin 1	9267
235421_at	0.0681	1.76E-03	-1.2456247	MAP3K8	mitogen-activated protein kinase kinase kinase 8	1326
222044_at	0.0683	1.78E-03	-1.2785953	LOC643549///PCIF1	uncharacterized LOC643549///PDX1 C-terminal inhibiting factor 1	643549///63935
222633_at	0.0683	1.78E-03	-1.2836147	TBL1XR1	transducin (beta)-like 1 X-linked receptor 1	79718
202250_s_at	0.0684	1.79E-03	-1.0735227	DCAF8	DDB1 and CUL4 associated factor 8	50717
232030_at	0.0684	1.80E-03	-1.1928487	EPG5	ectopic P-granules autophagy protein 5 homolog	57724
228801_at	0.0684	1.82E-03	-1.1697447	ORMDL1	ORMDL sphingolipid biosynthesis regulator 1	94101

1557736_at	0.0684	1.82E-03	-1.005576	NKTR	natural killer cell triggering receptor	4820
231956_at	0.0684	1.82E-03	-1.07314	RNF213	ring finger protein 213	57674
208705_s_at	0.0685	1.83E-03	-1.2332387	EIF5	eukaryotic translation initiation factor 5	1983
223494_at	0.0685	1.84E-03	-1.1254607	MGEA5	meningioma expressed antigen 5 (hyaluronidase)	10724
229519_at	0.0685	1.85E-03	-1.0023933	FXR1	FMR1 autosomal homolog 1	8087
214917_at	0.0686	1.86E-03	-1.2995687	PRKAA1	protein kinase AMP-activated catalytic subunit alpha 1	5562
226952_at	0.0686	1.88E-03	-1.5636393	EAF1	ELL associated factor 1	85403
1554961_at	0.0686	1.88E-03	1.11608	FGFR4	fibroblast growth factor receptor 4	2264
212413_at	0.0686	1.90E-03	-1.120598	SEPT6	septin 6	23157

201329_s_at	0.0688	1.93E-03	-1.035054	ETS2	ETS proto-oncogene 2, transcription factor	2114
208720_s_at	0.0688	1.95E-03	-1.4102487	RBM39	RNA binding motif protein 39	9584
221313_at	0.0688	1.96E-03	1.058076	GPR52	G protein-coupled receptor 52	9293
228318_s_at	0.0689	1.98E-03	-1.2405867	CRIPAK	cysteine rich PAK1 inhibitor	285464
210046_s_at	0.0689	1.99E-03	-1.0767747	IDH2	isocitrate dehydrogenase (NADP(+)) 2, mitochondrial	3418
228196_s_at	0.0689	2.00E-03	1.011806	LARP4B	La ribonucleoprotein domain family member 4B	23185
211386_at	0.0689	2.00E-03	-1.044804	MGC12488	uncharacterized protein MGC12488	84786
1553704_x_at	0.0689	2.00E-03	-1.042702	ZNF791	zinc finger protein 791	163049
210321_at	0.0691	2.01E-03	-1.5112873	GZMH	granzyme H	2999

1554250_s_at	0.0691	2.02E-03	-1.2016227	STAG3L2///STAG3L3///TRIM73	stromal antigen 3-like 2 (pseudogene)///stromal antigen 3-like 3 (pseudogene)///tripartite motif containing 73	442582///442578///375593
234773_x_at	0.0691	2.03E-03	1.129668	LOC101928288	uncharacterized LOC101928288	101928288
1561393_at	0.0691	2.03E-03	1.1289693	KIAA1755	KIAA1755	85449
204028_s_at	0.0691	2.03E-03	-1.0497767	RABGAP1	RAB GTPase activating protein 1	23637
214693_x_at	0.0691	2.04E-03	-1.0352253	NBPF26///NBPF10///NBPF9///NBPF14	neuroblastoma breakpoint family member 26///neuroblastoma breakpoint family member 10///neuroblastoma breakpoint family member 9///neuroblastoma breakpoint family member 14	101060684///100132406///400818///25832

237741_at	0.0691	2.04E-03	-1.0163833	SLC25A36	solute carrier family 25 member 36	55186
203659_s_at	0.0691	2.06E-03	-1.2023213	TRIM13	tripartite motif containing 13	10206
225724_at	0.0691	2.07E-03	-1.3887787	PSMA3-AS1	PSMA3 antisense RNA 1	379025
214446_at	0.0691	2.07E-03	1.0472053	ELL2	elongation factor for RNA polymerase II 2	22936
221768_at	0.0691	2.07E-03	-1.0268247	SFPQ	splicing factor proline and glutamine rich	6421
214753_at	0.0691	2.07E-03	-1.322226	N4BP2L2	NEDD4 binding protein 2 like 2	10443
214683_s_at	0.0691	2.07E-03	-1.108936	CLK1	CDC like kinase 1	1195
207760_s_at	0.0691	2.10E-03	-1.1877687	NCOR2	nuclear receptor corepressor 2	9612
228662_at	0.0691	2.10E-03	-1.0288687	SOCS7	suppressor of cytokine signaling 7	30837
223598_at	0.0691	2.10E-03	-1.1556833	RAD23B	RAD23 homolog B, nucleotide excision repair protein	5887

74694_s_at	0.0691	2.11E-03	-1.2725787	RABEP2	rabaptin, RAB GTPase binding effector protein 2	79874
1562826_at	0.0691	2.11E-03	1.0993827	PLCE1-AS2	PLCE1 antisense RNA 2	101927049
220580_at	0.0691	2.12E-03	1.02422	BICC1	BicC family RNA binding protein 1	80114
1561624_at	0.0691	2.12E-03	1.1109327	LOC101927537	uncharacterized LOC101927537	101927537
225740_x_at	0.0692	2.13E-03	-1.2473933	MDM4	MDM4, p53 regulator	4194
229017_s_at	0.0693	2.14E-03	-1.023998	DSTYK	dual serine/threonine and tyrosine protein kinase	25778
226505_x_at	0.0694	2.15E-03	-1.3628327	USP32	ubiquitin specific peptidase 32	84669
239163_at	0.0695	2.17E-03	-1.0818493	UBE2B	ubiquitin conjugating enzyme E2 B	7320
208869_s_at	0.0698	2.19E-03	-1.0601453	GABARAPL1	GABA type A receptor associated protein like 1	23710

229686_at	0.0698	2.19E-03	-1.1850067	P2RY8	purinergic receptor P2Y8	286530
225929_s_at	0.0699	2.20E-03	-1.1421813	RNF213	ring finger protein 213	57674
212420_at	0.0699	2.21E-03	-1.014964	ELF1	E74 like ETS transcription factor 1	1997
203542_s_at	0.0699	2.21E-03	-1.1600213	KLF9	Kruppel like factor 9	687
243857_at	0.0699	2.21E-03	-1.241432	MORF4L2	mortality factor 4 like 2	9643
230270_at	0.0699	2.21E-03	-1.4877393	PRPF38B	pre-mRNA processing factor 38B	55119
232521_at	0.07	2.22E-03	-1.163608	PCSK7	proprotein convertase subtilisin/kexin type 7	9159
225640_at	0.07	2.24E-03	-1.1446227	EBLN3P	endogenous Bornavirus-like nucleoprotein 3, pseudogene	100506710
201804_x_at	0.0703	2.26E-03	-1.0945607	TBCB	tubulin folding cofactor B	1155
226304_at	0.0703	2.26E-03	1.0003487	HSPB6	heat shock protein family B (small) member 6	126393

239486_at	0.0703	2.27E-03	-1.26548	REL	REL proto-oncogene, NF-kB subunit	5966
232145_at	0.0703	2.27E-03	-1.1837713	C2orf68	chromosome 2 open reading frame 68	388969
225552_x_at	0.0703	2.28E-03	-1.0369907	AURKAIP1	aurora kinase A interacting protein 1	54998
214783_s_at	0.0705	2.29E-03	-1.1053153	ANXA11	annexin A11	311
218206_x_at	0.0705	2.29E-03	-1.214744	SCAND1	SCAN domain containing 1	51282
215275_at	0.0705	2.30E-03	-1.023502	TRAF3IP3	TRAF3 interacting protein 3	80342
243648_at	0.0705	2.31E-03	-1.0169153	ZBED6	zinc finger BED-type containing 6	100381270
203742_s_at	0.0709	2.40E-03	-1.00454	LOC732360///TDG	G/T mismatch-specific thymine DNA glycosylase-like///thymine DNA glycosylase	732360///6996

212665_at	0.0709	2.40E-03	-1.0881327	TIPARP	TCDD inducible poly(ADP-ribose) polymerase	25976
213156_at	0.0709	2.41E-03	-1.1803393	ZBTB20	zinc finger and BTB domain containing 20	26137
1554240_a_at	0.0711	2.42E-03	-1.1697993	ITGAL	integrin subunit alpha L	3683
213475_s_at	0.0711	2.43E-03	-1.0136567	ITGAL	integrin subunit alpha L	3683
1559018_at	0.0711	2.43E-03	-1.0282693	PTPRE	protein tyrosine phosphatase, receptor type E	5791
227588_s_at	0.0713	2.46E-03	1.0035993	GET4	golgi to ER traffic protein 4	51608
229060_at	0.0714	2.47E-03	-1.0118747	YPEL2	yippee like 2	388403
218496_at	0.0714	2.47E-03	-1.1413993	RNASEH1	ribonuclease H1	246243
212697_at	0.0718	2.49E-03	-1.643342	FAM134C	family with sequence similarity 134 member C	162427
202596_at	0.0718	2.49E-03	-1.431036	ENSA	endosulfine alpha	2029

212506_at	0.0719	2.51E-03	-1.813328	PICALM	phosphatidylinositol binding clathrin assembly protein	8301
238121_at	0.0719	2.51E-03	-1.1355227	GK5	glycerol kinase 5 (putative)	256356
1554251_at	0.0721	2.53E-03	-1.111156	HP1BP3	heterochromatin protein 1 binding protein 3	50809
233647_s_at	0.0721	2.53E-03	-1.0693567	CDADC1	cytidine and dCMP deaminase domain containing 1	81602
218034_at	0.0722	2.54E-03	-1.0034193	FIS1	fission, mitochondrial 1	51024
228562_at	0.0723	2.55E-03	-1.16628	ZBTB10	zinc finger and BTB domain containing 10	65986
1556797_at	0.0723	2.55E-03	1.1341767	RNF144A-AS1	RNF144A antisense RNA 1	386597
208078_s_at	0.0723	2.55E-03	-1.0670713	SIK1	salt inducible kinase 1	150094
232431_at	0.0723	2.56E-03	-1.762888	NR3C1	nuclear receptor subfamily 3 group C member 1	2908

225565_at	0.0723	2.57E-03	-1.05462	CREB1	cAMP responsive element binding protein 1	1385
235023_at	0.0723	2.58E-03	-1.07756	VPS13C	vacuolar protein sorting 13 homolog C	54832
1554986_a_at	0.0723	2.59E-03	1.734726	SNX19	sorting nexin 19	399979
209076_s_at	0.0723	2.59E-03	-1.0336853	WDR45B	WD repeat domain 45B	56270
210825_s_at	0.0725	2.61E-03	-1.0205513	PEBP1	phosphatidylethanolamine binding protein 1	5037
204621_s_at	0.0726	2.63E-03	-1.1248093	NR4A2	nuclear receptor subfamily 4 group A member 2	4929
210178_x_at	0.0726	2.63E-03	-1.0821153	SRSF10	serine and arginine rich splicing factor 10	10772
223130_s_at	0.0727	2.66E-03	-1.2381633	MYLIP	myosin regulatory light chain interacting protein	29116

242550_at	0.0727	2.66E-03	-1.0797393	EIF3B	eukaryotic translation initiation factor 3 subunit B	8662
202464_s_at	0.0728	2.69E-03	-1.5303153	PFKFB3	6-phosphofructo-2-kinase/fructose-2,6-biphosphatase 3	5209
210054_at	0.0728	2.69E-03	-1.0136773	HAUS3	HAUS augmin like complex subunit 3	79441
224925_at	0.0729	2.74E-03	-1.2039107	PREX1	phosphatidylinositol-3,4,5-trisphosphate dependent Rac exchange factor 1	57580

1554089_s_at	0.0731	2.76E-03	-1.1974573	SBDSP1///SBDS	Shwachman-Bodian-Diamond syndrome pseudogene 1///SBDS ribosome assembly guanine nucleotide exchange factor	155370///51119
208714_at	0.0732	2.77E-03	-1.1052627	NDUFV1	NADH:ubiquinone oxidoreductase core subunit V1	4723
240337_at	0.0734	2.78E-03	1.0246147	RHOA	ras homolog family member A	387
204622_x_at	0.0735	2.79E-03	-1.045676	NR4A2	nuclear receptor subfamily 4 group A member 2	4929
223411_at	0.0738	2.82E-03	-1.0041487	MIF4GD	MIF4G domain containing	57409
214722_at	0.0739	2.84E-03	-1.0231673	NOTCH2NL	notch 2 N-terminal like	388677
210312_s_at	0.0739	2.84E-03	-1.235818	IFT20	intraflagellar transport 20	90410

221989_at	0.074	2.86E-03	-1.11307	SNORA70///RPL10	small nucleolar RNA, H/ACA box 70///ribosomal protein L10	26778///6134
221918_at	0.074	2.86E-03	-1.1060813	CDK17	cyclin dependent kinase 17	5128
201527_at	0.0743	2.89E-03	-1.0628607	LOC101927180///ATP6V1F	V-type proton ATPase subunit F pseudogene///ATPase H+ transporting V1 subunit F	101927180///9296
212847_at	0.0744	2.90E-03	-1.2379707	FUBP1	far upstream element binding protein 1	8880
213857_s_at	0.0744	2.90E-03	-1.130866	CD47	CD47 molecule	961
1559882_at	0.0745	2.91E-03	-1.314536	SAMHD1	SAM and HD domain containing deoxynucleoside triphosphate triphosphohydrolase 1	25939
228291_s_at	0.0746	2.92E-03	-1.065038	KIZ	kizuna centrosomal protein	55857

1569428_at	0.0746	2.93E-03	1.1149553	PYM1	PYM homolog 1, exon junction complex associated factor	84305
204731_at	0.0746	2.93E-03	-1.5576513	TGFBR3	transforming growth factor beta receptor 3	7049
217965_s_at	0.0747	2.95E-03	-1.0330787	SAP30BP	SAP30 binding protein	29115
204710_s_at	0.0747	2.95E-03	-1.0852067	WIPI2	WD repeat domain, phosphoinositide interacting 2	26100
228713_s_at	0.0748	2.97E-03	1.0666253	HSD17B14	hydroxysteroid 17-beta dehydrogenase 14	51171
202191_s_at	0.0748	2.98E-03	-1.1567573	GAS7	growth arrest specific 7	8522
238736_at	0.0749	3.00E-03	-1.1741713	REV3L	REV3 like, DNA directed polymerase zeta catalytic subunit	5980
235242_at	0.0749	3.00E-03	-1.319224	REL	REL proto-oncogene, NF-kB subunit	5966

201954_at	0.0749	3.01E-03	-1.7133147	ARPC1B	actin related protein 2/3 complex subunit 1B	10095
214369_s_at	0.0749	3.02E-03	-1.0167013	RASGRP2	RAS guanyl releasing protein 2	10235
225199_at	0.0749	3.02E-03	-1.2278333	C16orf72	chromosome 16 open reading frame 72	29035
228725_x_at	0.0749	3.02E-03	-1.0093753	PRMT2	protein arginine methyltransferase 2	3275
200058_s_at	0.075	3.03E-03	-1.0913967	LOC101929240///SNRNP200	U5 small nuclear ribonucleoprotein 200 kDa helicase pseudogene///small nuclear ribonucleoprotein U5 subunit 200	101929240///23020
220704_at	0.0751	3.04E-03	-1.3090373	IKZF1	IKAROS family zinc finger 1	10320
240381_at	0.0753	3.06E-03	1.002004	ZBED5	zinc finger BED-type containing 5	58486

200811_at	0.0753	3.06E-03	-1.473454	CIRBP	cold inducible RNA binding protein	1153
239106_at	0.0753	3.07E-03	-1.238348	CA5BP1	carbonic anhydrase 5B pseudogene 1	340591
213326_at	0.0756	3.09E-03	-1.1421333	VAMP1	vesicle associated membrane protein 1	6843
203332_s_at	0.0756	3.10E-03	-1.0059593	INPP5D	inositol polyphosphate-5-phosphatase D	3635
217860_at	0.0757	3.12E-03	-1.2899067	NDUFA10	NADH:ubiquinone oxidoreductase subunit A10	4705
212723_at	0.0757	3.12E-03	-1.1609247	JMJD6	arginine demethylase and lysine hydroxylase	23210
1564333_a_at	0.0758	3.13E-03	1.082476	PSAPL1	prosaposin-like 1 (gene/pseudogene)	768239
229813_x_at	0.0758	3.13E-03	-1.146712	DAZAP1	DAZ associated protein 1	26528
212119_at	0.0759	3.14E-03	-1.2418047	RHOQ	ras homolog family member Q	23433

219117_s_at	0.0763	3.21E-03	-1.2186407	FKBP11	FK506 binding protein 11	51303
227979_at	0.0765	3.23E-03	-1.0993167	RBM4	RNA binding motif protein 4	5936
214894_x_at	0.0767	3.26E-03	-1.0329913	MACF1	microtubule-actin crosslinking factor 1	23499
213594_x_at	0.0768	3.27E-03	-1.0902613	SRSF10	serine and arginine rich splicing factor 10	10772
236033_at	0.077	3.30E-03	1.2582787	ASB12	ankyrin repeat and SOCS box containing 12	142689
205022_s_at	0.077	3.31E-03	-1.1775793	FOXN3	forkhead box N3	1112
201587_s_at	0.077	3.31E-03	-1.1331847	IRAK1	interleukin 1 receptor associated kinase 1	3654
227932_at	0.077	3.32E-03	-1.111356	ARIH2	ariadne RBR E3 ubiquitin protein ligase 2	10425
229032_at	0.0773	3.34E-03	1.0766947	WSCD2	WSC domain containing 2	9671
209682_at	0.0773	3.36E-03	-1.0396073	CBLB	Cbl proto-oncogene B	868

212886_at	0.0774	3.36E-03	-1.347022	CCDC69	coiled-coil domain containing 69	26112
200892_s_at	0.0774	3.38E-03	-1.185392	TRA2B	transformer 2 beta homolog (Drosophila)	6434
1565484_x_at	0.0774	3.41E-03	1.8142707	EGFR	epidermal growth factor receptor	1956
217529_at	0.0774	3.41E-03	-1.2823413	ORAI2	ORAI calcium release-activated calcium modulator 2	80228
225955_at	0.0774	3.41E-03	-1.172444	METRNL	meteorin like, glial cell differentiation regulator	284207
1554036_at	0.0774	3.43E-03	-1.421812	ZBTB24	zinc finger and BTB domain containing 24	9841
213703_at	0.0774	3.44E-03	-1.0680413	LINC00342	long intergenic non-protein coding RNA 342	150759
205511_at	0.0776	3.47E-03	-1.3888313	FLJ10038	uncharacterized protein FLJ10038	55056

208442_s_at	0.0777	3.49E-03	-1.3062113	ATM	ATM serine/threonine kinase	472
236814_at	0.078	3.54E-03	-1.0586913	MDM4	MDM4, p53 regulator	4194
228991_at	0.0781	3.54E-03	-1.001126	CDK13	cyclin dependent kinase 13	8621
221778_at	0.0781	3.55E-03	-1.30026	KDM7A	lysine demethylase 7A	80853
221870_at	0.0781	3.56E-03	1.004954	EHD2	EH domain containing 2	30846
1562719_at	0.0781	3.57E-03	1.1014147	LOC101928476	uncharacterized LOC101928476	101928476
214499_s_at	0.0781	3.58E-03	-1.1198553	BCLAF1	BCL2 associated transcription factor 1	9774
232652_x_at	0.0782	3.63E-03	-1.2353047	SCAND1	SCAN domain containing 1	51282
201232_s_at	0.0785	3.67E-03	-1.4928713	PSMD13	proteasome 26S subunit, non-ATPase 13	5719
212852_s_at	0.0787	3.69E-03	-1.2213973	TROVE2	TROVE domain family member 2	6738
201394_s_at	0.0787	3.70E-03	-1.24361	RBM5	RNA binding motif protein 5	10181

221972_s_at	0.0788	3.75E-03	-1.4422953	SDF4	stromal cell derived factor 4	51150
1565743_at	0.0788	3.76E-03	-1.05475	N4BP2L2	NEDD4 binding protein 2 like 2	10443
219256_s_at	0.0789	3.79E-03	-1.310112	SH3TC1	SH3 domain and tetratricopeptide repeats 1	54436
221899_at	0.0789	3.80E-03	-1.065254	N4BP2L2	NEDD4 binding protein 2 like 2	10443
218286_s_at	0.0792	3.83E-03	-1.1084133	RNF7	ring finger protein 7	9616
214280_x_at	0.0794	3.85E-03	-1.1241427	HNRNPA1	heterogeneous nuclear ribonucleoprotein A1	3178
230180_at	0.0795	3.86E-03	-1.0348147	DDX17	DEAD-box helicase 17	10521
210092_at	0.0795	3.86E-03	-1.270044	MAGOHB///MAGOH	mago homolog B, exon junction complex core component///mago homolog, exon junction complex core component	55110///4116

216194_s_at	0.0795	3.86E-03	-1.2965987	TBCB	tubulin folding cofactor B	1155
203822_s_at	0.0796	3.91E-03	-1.0823267	ELF2	E74 like ETS transcription factor 2	1998
241774_at	0.0799	3.94E-03	-1.084864	PSMA3-AS1	PSMA3 antisense RNA 1	379025
221829_s_at	0.08	3.95E-03	-1.1349367	TNPO1	transportin 1	3842
243361_at	0.08	3.95E-03	-1.0922927	SREK1	splicing regulatory glutamic acid and lysine rich protein 1	140890
216248_s_at	0.08	3.96E-03	-1.102532	NR4A2	nuclear receptor subfamily 4 group A member 2	4929
1557828_a_at	0.08	3.96E-03	-1.1083413	TMEM267	transmembrane protein 267	64417
218723_s_at	0.08	3.97E-03	-1.1795787	RGCC	regulator of cell cycle	28984
232909_s_at	0.08	3.97E-03	-1.222172	BPTF	bromodomain PHD finger transcription factor	2186
225698_at	0.0805	4.06E-03	-1.229068	EPB41L4A-AS1	EPB41L4A antisense RNA 1	114915

225827_at	0.0805	4.07E-03	-1.258786	AGO2	argonaute 2, RISC catalytic component	27161
207460_at	0.0805	4.07E-03	-1.2487427	GZMM	granzyme M	3004
208686_s_at	0.0805	4.07E-03	-1.11403	BRD2	bromodomain containing 2	6046
224375_at	0.0806	4.08E-03	1.1085933	LOC102724870	uncharacterized LOC102724870	102724870
1565947_a_at	0.0806	4.08E-03	1.1471027	CHML	CHM like, Rab escort protein 2	1122
1553133_at	0.0806	4.09E-03	-1.4145273	C9orf72	chromosome 9 open reading frame 72	203228
244654_at	0.0806	4.10E-03	-1.0703633	MYO1G	myosin IG	64005
222018_at	0.0808	4.11E-03	-1.2782553	NACA	nascent polypeptide-associated complex alpha subunit	4666
205281_s_at	0.0809	4.14E-03	-1.036586	PIGA	phosphatidylinositol glycan anchor biosynthesis class A	5277
214348_at	0.081	4.16E-03	1.0177127	TACR2	tachykinin receptor 2	6865
217783_s_at	0.0813	4.19E-03	-1.0864013	YPEL5	yippee like 5	51646

207001_x_at	0.0815	4.21E-03	-1.185886	TSC22D3	TSC22 domain family member 3	1831
233167_at	0.0815	4.23E-03	-1.016836	SELO	selenoprotein O	83642
202761_s_at	0.0816	4.24E-03	-1.129892	SYNE2	spectrin repeat containing nuclear envelope protein 2	23224
212826_s_at	0.0819	4.30E-03	-1.357894	SLC25A6	solute carrier family 25 member 6	293
232312_at	0.0819	4.30E-03	-1.1444527	PPP6R3	protein phosphatase 6 regulatory subunit 3	55291
1554889_at	0.0819	4.30E-03	1.117876	TIA1	TIA1 cytotoxic granule-associated RNA binding protein	7072
243981_at	0.082	4.32E-03	-1.1523447	STK4	serine/threonine kinase 4	6789
1569583_at	0.082	4.32E-03	-1.029812	EREG	epiregulin	2069
213280_at	0.0822	4.36E-03	-1.0478573	RAP1GAP2	RAP1 GTPase activating protein 2	23108
236019_at	0.0825	4.41E-03	-1.1596273	RAB12	RAB12, member RAS oncogene family	201475

1555847_a_at	0.0825	4.42E-03	-1.196416	MIR24-2///MIR23A///LOC284454	microRNA 24-2///microRNA 23a///uncharacterized LOC284454	407013///407010///284454
207079_s_at	0.0826	4.44E-03	-1.0580033	MED6	mediator complex subunit 6	10001
212775_at	0.0827	4.46E-03	1.238108	OBSL1	obscurin like 1	23363
204698_at	0.0827	4.47E-03	-1.078228	ISG20	interferon stimulated exonuclease gene 20	3669
227082_at	0.0827	4.48E-03	-1.2254987	ZBTB20	zinc finger and BTB domain containing 20	26137
202206_at	0.0827	4.49E-03	-1.2562133	ARL4C	ADP ribosylation factor like GTPase 4C	10123
212225_at	0.0828	4.54E-03	-1.3592173	EIF1	eukaryotic translation initiation factor 1	10209
215322_at	0.0828	4.54E-03	-1.1616387	LONRF1	LON peptidase N-terminal domain and ring finger 1	91694

212566_at	0.0828	4.55E-03	-1.073186	MAP4	microtubule associated protein 4	4134
218940_at	0.0828	4.59E-03	-1.2291473	VCPKMT	valosin containing protein lysine methyltransferase	79609
201745_at	0.0829	4.61E-03	-1.3502713	TWF1	twinfilin actin binding protein 1	5756
233819_s_at	0.0831	4.64E-03	-1.2259907	LTN1	listerin E3 ubiquitin protein ligase 1	26046
215123_at	0.0832	4.67E-03	-1.0721667	NPIPA5///NPIPB5///LOC613037///NPIPB4///NPIPB3	nuclear pore complex interacting protein family member A5///nuclear pore complex interacting protein family member B5///nuclear pore complex interacting protein member///nuclear pore complex interacting protein family member B4///nucl	100288332///100132247///613037///440345///23117

214163_at	0.0833	4.68E-03	-1.175428	HSPB11	heat shock protein family B (small) member 11	51668
226352_at	0.0833	4.68E-03	-1.434834	JMY	junction mediating and regulatory protein, p53 cofactor	133746
204897_at	0.0834	4.69E-03	-1.0208607	PTGER4	prostaglandin E receptor 4	5734
200043_at	0.0834	4.73E-03	-1.6301587	ERH	enhancer of rudimentary homolog (Drosophila)	2079
235199_at	0.0834	4.73E-03	-1.2105313	RNF125	ring finger protein 125	54941
240592_at	0.0836	4.76E-03	-1.4115533	LCORL	ligand dependent nuclear receptor corepressor like	254251
210616_s_at	0.0836	4.77E-03	-1.091	SEC31A	SEC31 homolog A, COPII coat complex component	22872
212256_at	0.0837	4.79E-03	-1.0946213	GALNT10	polypeptide N-acetylgalactosaminyltransferase 10	55568

230532_at	0.0842	4.86E-03	-1.186958	CXorf38	chromosome X open reading frame 38	159013
221495_s_at	0.0843	4.86E-03	-1.0215667	TCF25	transcription factor 25	22980
209300_s_at	0.0843	4.87E-03	-1.1292433	NECAP1	NECAP endocytosis associated 1	25977
225899_x_at	0.0843	4.89E-03	-1.409268	LOC101929819///LOC100133331///LOC100133182///LINC01001///LOC100132062///LOC729737///LINC01000///LINC01002	uncharacterized LOC101929819///uncharacterized LOC100133331///putative uncharacterized protein FLJ44672///long intergenic non-protein coding RNA 1001///uncharacterized LOC100132062///uncharacterized LOC729737///long	101929819///100133331///100133182///100133161///100132062///729737///402483///399844
235461_at	0.0845	4.92E-03	1.2680967	TET2	tet methylcytosine dioxygenase 2	54790
228135_at	0.0845	4.96E-03	-1.2561313	C1orf52	chromosome 1 open reading frame 52	148423

1555355_a_at	0.0846	4.97E-03	-1.0673487	ETS1	ETS proto-oncogene 1, transcription factor	2113
233019_at	0.0847	5.04E-03	-1.2131967	CNOT7	CCR4-NOT transcription complex subunit 7	29883
226110_at	0.0847	5.05E-03	-1.1522193	PTAR1	protein prenyltransferase alpha subunit repeat containing 1	375743
202839_s_at	0.0848	5.06E-03	-1.12033	NDUFB7	NADH:ubiquinone oxidoreductase subunit B7	4713
203543_s_at	0.0848	5.06E-03	-1.4038567	KLF9	Kruppel like factor 9	687
205070_at	0.0848	5.08E-03	-1.116364	ING3	inhibitor of growth family member 3	54556
231863_at	0.0848	5.09E-03	-1.130996	ING3	inhibitor of growth family member 3	54556
227726_at	0.0848	5.12E-03	-1.425242	RNF166	ring finger protein 166	115992

222688_at	0.0848	5.14E-03	-1.2185913	ACER3	alkaline ceramidase 3	55331
227931_at	0.0848	5.15E-03	-1.2082387	INO80D	INO80 complex subunit D	54891
209265_s_at	0.0849	5.19E-03	-1.1179033	METTL3	methyltransferase like 3	56339
228153_at	0.085	5.21E-03	-1.4534133	RNF144B	ring finger protein 144B	255488
1555920_at	0.085	5.21E-03	-1.150402	CBX3	chromobox 3	11335
219384_s_at	0.085	5.23E-03	-1.0235567	ADAT1	adenosine deaminase, tRNA specific 1	23536
208840_s_at	0.085	5.24E-03	-1.059874	G3BP2	G3BP stress granule assembly factor 2	9908
228053_s_at	0.085	5.27E-03	-1.3313513	TOMM5	translocase of outer mitochondrial membrane 5	401505
208821_at	0.085	5.27E-03	-1.055998	SNRPB	small nuclear ribonucleoprotein polypeptides B and B1	6628
1565849_a_at	0.085	5.28E-03	1.0124147	SRRM5	serine/arginine repetitive matrix 5	100170229

225951_s_at	0.085	5.29E-03	-1.0153413	LINC01578	long intergenic non-protein coding RNA 1578	100507217
222494_at	0.0851	5.30E-03	-1.1895147	FOXN3	forkhead box N3	1112
223085_at	0.0852	5.31E-03	-1.0938113	RNF19A	ring finger protein 19A, RBR E3 ubiquitin protein ligase	25897
53071_s_at	0.0855	5.36E-03	-1.2808633	OGFOD3	2-oxoglutarate and iron dependent oxygenase domain containing 3	79701
208961_s_at	0.0855	5.36E-03	-1.339194	KLF6	Kruppel like factor 6	1316
1553974_at	0.0859	5.43E-03	-1.117424	C22orf39	chromosome 22 open reading frame 39	128977
1553102_a_at	0.0859	5.45E-03	-1.3887307	CCDC69	coiled-coil domain containing 69	26112
229501_s_at	0.0861	5.47E-03	-1.060318	USP8	ubiquitin specific peptidase 8	9101

222808_at	0.0862	5.52E-03	-1.1560147	ALG13	ALG13, UDP-N-acetylglucosaminyltransferase subunit	79868
230416_at	0.0863	5.56E-03	-1.0064513	PDZD8	PDZ domain containing 8	118987
203884_s_at	0.0866	5.64E-03	-1.0491093	RAB11FIP2	RAB11 family interacting protein 2	22841
214790_at	0.0867	5.71E-03	-1.0243973	SENP6	SUMO1/sentrin specific peptidase 6	26054
221499_s_at	0.0867	5.72E-03	-1.3031073	STX16	syntaxin 16	8675
225461_at	0.0867	5.76E-03	-1.010746	EHMT1	euchromatic histone lysine methyltransferase 1	79813
203723_at	0.0867	5.77E-03	-1.1039827	ITPKB	inositol-trisphosphate 3-kinase B	3707
202314_at	0.0868	5.79E-03	-1.1673153	LRRD1///CYP51A1	leucine rich repeats and death domain containing 1///cytochrome P450 family 51 subfamily A member 1	401387///1595

1568249_at	0.0868	5.79E-03	1.33907	SNHG17///SNORA71B	small nucleolar RNA host gene 17///small nucleolar RNA, H/ACA box 71B	388796///26776
222088_s_at	0.0868	5.80E-03	-1.3591013	SLC2A14///SLC2A3	solute carrier family 2 member 14///solute carrier family 2 member 3	144195///6515
223271_s_at	0.0872	5.90E-03	-1.0212433	CTDSPL2	CTD small phosphatase like 2	51496

228370_at	0.0872	5.90E-03	-1.020058	LOC101930404///SNORD116-28///SNORD115-26///SNORD115-13///SNORD115-7///SNORD116-22///SNORD116-4///PWARSN///SNORD107///IPW	uncharacterized LOC101930404///small nucleolar RNA, C/D box 116-28///small nucleolar RNA, C/D box 115-26///small nucleolar RNA, C/D box 115-13///small nucleolar RNA, C/D box 115-7///small nucleolar RNA, C/D box 116-22///small nucleolar RNA, C/D box 116-	101930404///100033820///100033802///100033450///100033444///100033433///100033416///347746///91380///3653
201553_s_at	0.0872	5.91E-03	-1.1668233	LAMP1	lysosomal associated membrane protein 1	3916
225372_at	0.0877	6.00E-03	-1.3520507	C10orf54	chromosome 10 open reading frame 54	64115
226914_at	0.0877	6.04E-03	-1.148858	ARPC5L	actin related protein 2/3 complex subunit 5 like	81873
208691_at	0.0877	6.04E-03	-1.3524267	TFRC	transferrin receptor	7037

227708_at	0.0877	6.04E-03	-1.3394993	EEF1A1	eukaryotic translation elongation factor 1 alpha 1	1915
227129_x_at	0.0877	6.05E-03	-1.172078	LINC01000	long intergenic non-protein coding RNA 1000	402483
213019_at	0.0877	6.05E-03	-1.2777907	RANBP6	RAN binding protein 6	26953
211474_s_at	0.0877	6.07E-03	-1.051444	SERPINB6	serpin family B member 6	5269
203508_at	0.0877	6.08E-03	-1.5656427	TNFRSF1B	TNF receptor superfamily member 1B	7133
204285_s_at	0.0877	6.09E-03	-1.3199453	PMAIP1	phorbol-12-myristate-13-acetate-induced protein 1	5366
204299_at	0.0877	6.10E-03	-1.3213987	SRSF10	serine and arginine rich splicing factor 10	10772
224922_at	0.0879	6.13E-03	-1.2259933	CSNK2A2	casein kinase 2 alpha 2	1459
201871_s_at	0.0879	6.15E-03	-1.12209	UBXN1	UBX domain protein 1	51035

208930_s_at	0.088	6.17E-03	-1.0513747	ILF3	interleukin enhancer binding factor 3	3609
1569206_at	0.0884	6.25E-03	1.317988	TCP11L2	t-complex 11 like 2	255394
226399_at	0.0884	6.25E-03	-1.585884	DNAJB14	DnaJ heat shock protein family (Hsp40) member B14	79982
218381_s_at	0.0884	6.26E-03	-1.014716	U2AF2	U2 small nuclear RNA auxiliary factor 2	11338
239629_at	0.0884	6.26E-03	-1.3756047	CFLAR	CASP8 and FADD like apoptosis regulator	8837

214805_at	0.0884	6.26E-03	-1.0926573	SNORD10///SNORA48///SNORA67///EIF4A1	small nucleolar RNA, C/D box 10///small nucleolar RNA, H/ACA box 48///small nucleolar RNA, H/ACA box 67///eukaryotic translation initiation factor 4A1	652966///652965///26781///1973
243109_at	0.0885	6.30E-03	-1.0696873	MCTP2	multiple C2 and transmembrane domain containing 2	55784
235067_at	0.0885	6.31E-03	-1.239724	MKLN1	muskelin 1	4289
203190_at	0.0886	6.33E-03	-1.137926	MIR7113///MIR4691///NDUFS8	microRNA 7113///microRNA 4691///NADH:ubiquinone oxidoreductase core subunit S8	102465669///100616403///4728
223134_at	0.0888	6.34E-03	-1.25127	BBX	BBX, HMG-box containing	56987

225219_at	0.0888	6.35E-03	-1.016094	SMAD5	SMAD family member 5	4090
201999_s_at	0.0888	6.35E-03	-1.1634787	DYNLT1	dynein light chain Tctex-type 1	6993
208615_s_at	0.089	6.39E-03	-1.37654	PTP4A2	protein tyrosine phosphatase type IVA, member 2	8073
1561093_at	0.0892	6.43E-03	1.073338	SLC22A25	solute carrier family 22 member 25	387601
1554638_at	0.0892	6.43E-03	-1.1657873	ZFYVE16	zinc finger FYVE-type containing 16	9765
222408_s_at	0.0892	6.44E-03	-1.045888	YPEL5	yippee like 5	51646
212462_at	0.0892	6.44E-03	-1.0917987	KAT6B	lysine acetyltransferase 6B	23522

1568609_s_at	0.0892	6.44E-03	-1.6187793	LOC103091866///LINC00623///LINC01138///LINC00869	uncharacterized LOC103091866///long intergenic non-protein coding RNA 623///long intergenic non-protein coding RNA 1138///long intergenic non-protein coding RNA 869	103091866///728855///388685///57234
226080_at	0.0893	6.50E-03	-1.2327053	SSH2	slingshot protein phosphatase 2	85464
211501_s_at	0.0896	6.53E-03	-1.18524	EIF3B	eukaryotic translation initiation factor 3 subunit B	8662
226422_at	0.0896	6.54E-03	-1.2759853	ERGIC2	ERGIC and golgi 2	51290
202416_at	0.0898	6.56E-03	-1.0753667	DNAJC7	DnaJ heat shock protein family (Hsp40) member C7	7266

222309_at	0.0901	6.61E-03	-1.2234213	C6orf62	chromosome 6 open reading frame 62	81688
200654_at	0.0901	6.61E-03	-1.217014	P4HB	prolyl 4-hydroxylase subunit beta	5034
226561_at	0.0902	6.62E-03	-1.3234327	AGFG1	ArfGAP with FG repeats 1	3267
230917_at	0.0903	6.69E-03	-1.1513427	PLCG2	phospholipase C gamma 2	5336
219229_at	0.0904	6.70E-03	-1.0777033	SLCO3A1	solute carrier organic anion transporter family member 3A1	28232
229908_s_at	0.0904	6.70E-03	-1.1585527	UNKL	unkempt family like zinc finger	64718
200656_s_at	0.0904	6.72E-03	-1.087986	P4HB	prolyl 4-hydroxylase subunit beta	5034
205594_at	0.0904	6.72E-03	-1.1732087	ZNF652	zinc finger protein 652	22834
224985_at	0.0904	6.72E-03	-1.0220127	NRAS	neuroblastoma RAS viral oncogene homolog	4893
224711_at	0.0906	6.81E-03	-1.2044313	YY1	YY1 transcription factor	7528

204891_s_at	0.0906	6.83E-03	-1.009488	LCK	LCK proto-oncogene, Src family tyrosine kinase	3932
225957_at	0.0906	6.83E-03	-1.24188	CREBRF	CREB3 regulatory factor	153222
209042_s_at	0.0906	6.86E-03	-1.15859	UBE2G2	ubiquitin conjugating enzyme E2 G2	7327
202531_at	0.0906	6.88E-03	-1.0080967	IRF1	interferon regulatory factor 1	3659
214274_s_at	0.0907	6.94E-03	-1.1477733	ACAA1	acetyl-CoA acyltransferase 1	30
227435_at	0.0907	6.94E-03	-1.0750133	USF3	upstream transcription factor family member 3	205717
224890_s_at	0.0907	6.96E-03	-1.3537793	LAMTOR4	late endosomal/lysosomal adaptor, MAPK and MTOR activator 4	389541
219402_s_at	0.0907	6.96E-03	-1.1289927	DERL1	derlin 1	79139

202207_at	0.0907	6.96E-03	-1.1262347	ARL4C	ADP ribosylation factor like GTPase 4C	10123
218348_s_at	0.0907	6.97E-03	-1.0872853	ZC3H7A	zinc finger CCCH-type containing 7A	29066
201953_at	0.0912	7.04E-03	-1.0572187	CIB1	calcium and integrin binding 1	10519
210916_s_at	0.0913	7.05E-03	-1.053944	CD44	CD44 molecule (Indian blood group)	960
225609_at	0.0914	7.07E-03	-1.1379567	GSR	glutathione-disulfide reductase	2936
213491_x_at	0.0916	7.10E-03	-1.095048	RPN2	ribophorin II	6185
202978_s_at	0.0918	7.12E-03	-1.1062047	CREBZF	CREB/ATF bZIP transcription factor	58487
203538_at	0.0919	7.15E-03	-1.0237813	CAMLG	calcium modulating ligand	819
225164_s_at	0.0922	7.21E-03	-1.3781447	EIF2AK4	eukaryotic translation initiation factor 2 alpha kinase 4	440275

217898_at	0.0923	7.23E-03	-1.4532413	EMC7	ER membrane protein complex subunit 7	56851
226650_at	0.0924	7.27E-03	-1.01854	ZFAND2A	zinc finger AN1-type containing 2A	90637
202156_s_at	0.0924	7.27E-03	-1.0104653	CELF2	CUGBP, Elav-like family member 2	10659
200751_s_at	0.0924	7.30E-03	-2.0248733	HNRNPC	heterogeneous nuclear ribonucleoprotein C (C1/C2)	3183
208095_s_at	0.0924	7.30E-03	-1.304442	SRP72	signal recognition particle 72	6731
210845_s_at	0.0925	7.32E-03	-1.302602	PLAUR	plasminogen activator, urokinase receptor	5329
226452_at	0.0925	7.34E-03	-1.1602353	PDK1	pyruvate dehydrogenase kinase 1	5163
205361_s_at	0.0925	7.34E-03	-1.5674153	PFDN4	prefoldin subunit 4	5203
222619_at	0.0925	7.36E-03	-1.3797067	ZNF281	zinc finger protein 281	23528

206656_s_at	0.0925	7.38E-03	-1.298668	APMAP	adipocyte plasma membrane associated protein	57136
211727_s_at	0.0925	7.38E-03	-1.134734	COX11	COX11, cytochrome c oxidase copper chaperone	1353
202824_s_at	0.0925	7.38E-03	-1.5420007	TCEB1	transcription elongation factor B subunit 1	6921
227973_at	0.0925	7.40E-03	-1.1644653	C2orf69	chromosome 2 open reading frame 69	205327
229993_at	0.0925	7.40E-03	1.1648113	ZCCHC3	zinc finger CCHC-type containing 3	85364
213026_at	0.0925	7.40E-03	-1.3687547	ATG12	autophagy related 12	9140
200007_at	0.0926	7.42E-03	-1.3601607	SRP14	signal recognition particle 14	6727

202822_at	0.0927	7.46E-03	-1.1100107	LPP	LIM domain containing preferred translocation partner in lipoma	4026
206059_at	0.0927	7.46E-03	-1.1170993	ZNF91	zinc finger protein 91	7644
202165_at	0.0929	7.51E-03	-1.3090493	PPP1R2	protein phosphatase 1 regulatory inhibitor subunit 2	5504
208810_at	0.0929	7.52E-03	-1.3980867	TMEM135///DNAJB6	transmembrane protein 135///DnaJ heat shock protein family (Hsp40) member B6	65084///10049
226154_at	0.0933	7.58E-03	-1.0399033	DNM1L	dynamin 1 like	10059
230734_x_at	0.0933	7.60E-03	-1.3107867	STRN	striatin	6801
211934_x_at	0.0933	7.61E-03	-1.0070173	GANAB	glucosidase II alpha subunit	23193
221704_s_at	0.0934	7.62E-03	-1.0010927	VPS37B	VPS37B, ESCRT-I subunit	79720
225390_s_at	0.0936	7.65E-03	-1.493086	KLF13	Kruppel like factor 13	51621

205027_s_at	0.0936	7.66E-03	-1.0951127	MAP3K8	mitogen-activated protein kinase kinase kinase 8	1326
213922_at	0.0937	7.68E-03	-1.081282	TTBK2	tau tubulin kinase 2	146057
202595_s_at	0.0937	7.68E-03	-1.1607113	LEPROTL1	leptin receptor overlapping transcript-like 1	23484
209258_s_at	0.0937	7.69E-03	-1.0000547	SMC3	structural maintenance of chromosomes 3	9126
203312_x_at	0.0937	7.70E-03	-1.196122	ARF6	ADP ribosylation factor 6	382
218669_at	0.0938	7.73E-03	-1.0250273	RAP2C	RAP2C, member of RAS oncogene family	57826
218217_at	0.0939	7.75E-03	-1.350252	SCPEP1	serine carboxypeptidase 1	59342
238725_at	0.0939	7.75E-03	-1.0208813	IRF1	interferon regulatory factor 1	3659
206055_s_at	0.0942	7.86E-03	-1.09283	SNRPA1	small nuclear ribonucleoprotein polypeptide A'	6627

222177_s_at	0.0942	7.88E-03	1.0198827	SCAND2P	SCAN domain containing 2 pseudogene	54581
203136_at	0.0942	7.88E-03	-1.304598	RABAC1	Rab acceptor 1	10567
218671_s_at	0.0944	7.91E-03	-1.2363907	ATPIF1	ATPase inhibitory factor 1	93974
226979_at	0.0944	7.94E-03	-1.0403053	MAP3K2	mitogen-activated protein kinase kinase kinase 2	10746
222495_at	0.0944	7.94E-03	-1.06614	TMEM167B	transmembrane protein 167B	56900
201960_s_at	0.0944	7.95E-03	-1.0681293	MYCBP2	MYC binding protein 2, E3 ubiquitin protein ligase	23077
235061_at	0.0944	7.95E-03	-1.2878027	PPM1K	protein phosphatase, Mg2+/Mn2+ dependent 1K	152926
227510_x_at	0.0944	7.96E-03	2.24587	MALAT1	metastasis associated lung adenocarcinoma transcript 1 (non-protein coding)	378938

207983_s_at	0.0944	7.97E-03	-1.31234	STAG2	stromal antigen 2	10735
212428_at	0.0944	7.99E-03	-1.4171027	KIAA0368	KIAA0368	23392
203095_at	0.0945	8.03E-03	-1.2056007	MTIF2	mitochondrial translational initiation factor 2	4528
223213_s_at	0.0945	8.04E-03	-1.0694513	ZHX1	zinc fingers and homeoboxes 1	11244
1556346_at	0.0948	8.12E-03	-1.037894	COTL1	coactosin like F-actin binding protein 1	23406
226267_at	0.0948	8.13E-03	-1.107358	JDP2	Jun dimerization protein 2	122953
229543_at	0.0948	8.13E-03	-1.11382	FAM26F	family with sequence similarity 26 member F	441168
228670_at	0.0949	8.16E-03	-1.108524	TEP1	telomerase associated protein 1	7011
210282_at	0.0949	8.17E-03	-1.1460867	ZMYM2	zinc finger MYM-type containing 2	7750

213305_s_at	0.0949	8.18E-03	-1.0350007	PPP2R5C	protein phosphatase 2 regulatory subunit B'gamma	5527
200797_s_at	0.095	8.20E-03	-1.2536327	MCL1	BCL2 family apoptosis regulator	4170
200929_at	0.0952	8.23E-03	-1.0938893	TMED10	transmembrane p24 trafficking protein 10	10972
209224_s_at	0.0952	8.23E-03	-1.2210387	NDUFA2	NADH:ubiquinone oxidoreductase subunit A2	4695
221802_s_at	0.0958	8.36E-03	-1.3870133	SHTN1	shootin 1	57698
1569136_at	0.0958	8.36E-03	-1.3869067	MGAT4A	mannosyl (alpha-1,3-)-glycoprotein beta-1,4-N-acetylglucosaminyltransferase, isozyme A	11320
32541_at	0.0958	8.37E-03	-1.0641167	PPP3CC	protein phosphatase 3 catalytic subunit gamma	5533

218570_at	0.0958	8.37E-03	-1.0246253	PTPMT1///KBTBD4	protein tyrosine phosphatase, mitochondrial 1///kelch repeat and BTB domain containing 4	114971///55709
202160_at	0.0958	8.37E-03	-1.20699	CREBBP	CREB binding protein	1387
225229_at	0.0959	8.39E-03	-1.2329733	AFF4	AF4/FMR2 family member 4	27125
228106_at	0.0962	8.46E-03	-1.2730567	DCAF16	DDB1 and CUL4 associated factor 16	54876
204258_at	0.0962	8.46E-03	-1.1668193	CHD1	chromodomain helicase DNA binding protein 1	1105
238761_at	0.0962	8.47E-03	-1.192216	ELK4	ELK4, ETS transcription factor	2005
63825_at	0.0962	8.49E-03	-1.157016	ABHD2	abhydrolase domain containing 2	11057
210438_x_at	0.0962	8.50E-03	-1.0518167	TROVE2	TROVE domain family member 2	6738
224885_s_at	0.0964	8.54E-03	-1.2793947	KRTCAP2	keratinocyte associated protein 2	200185

34031_i_at	0.0966	8.58E-03	-1.285034	KRIT1	KRIT1, ankyrin repeat containing	889
204690_at	0.0966	8.59E-03	-1.3014193	STX8	syntaxin 8	9482
208960_s_at	0.0966	8.60E-03	-1.3927147	KLF6	Kruppel like factor 6	1316
1558569_at	0.0967	8.64E-03	-1.036946	LOC100131541	uncharacterized LOC100131541	100131541
202671_s_at	0.0967	8.65E-03	-1.0053893	PDXK	pyridoxal (pyridoxine, vitamin B6) kinase	8566
226318_at	0.0968	8.69E-03	-1.1356693	TBRG1	transforming growth factor beta regulator 1	84897
1555349_a_at	0.0969	8.72E-03	-1.5519073	ITGB2	integrin subunit beta 2	3689
1555832_s_at	0.0971	8.74E-03	-1.2446787	KLF6	Kruppel like factor 6	1316
222407_s_at	0.0974	8.82E-03	-1.0302247	ZNF106	zinc finger protein 106	64397
221264_s_at	0.0974	8.82E-03	-1.0015773	TARDBP	TAR DNA binding protein	23435
1553658_at	0.0974	8.82E-03	1.0154207	LINC00896	long intergenic non-protein coding RNA 896	150197

213158_at	0.0975	8.85E-03	-1.0596067	ZBTB20	zinc finger and BTB domain containing 20	26137
208325_s_at	0.0976	8.88E-03	-1.0480093	AKAP13	A-kinase anchoring protein 13	11214
1555844_s_at	0.0977	8.89E-03	-1.343228	HNRNPM	heterogeneous nuclear ribonucleoprotein M	4670
227305_s_at	0.0977	8.90E-03	-1.0136473	SMCR8	Smith-Magenis syndrome chromosome region, candidate 8	140775
203743_s_at	0.0977	8.91E-03	-1.324242	TDG	thymine DNA glycosylase	6996
225119_at	0.0977	8.93E-03	-1.0180407	CHMP4B	charged multivesicular body protein 4B	128866
221230_s_at	0.0978	8.99E-03	-1.1152693	ARID4B	AT-rich interaction domain 4B	51742
243463_s_at	0.0979	9.01E-03	-1.0918827	RIT1	Ras like without CAAX 1	6016
217427_s_at	0.098	9.08E-03	-1.10002	HIRA	histone cell cycle regulator	7290

225260_s_at	0.0981	9.15E-03	-1.1682427	MRPL32	mitochondrial ribosomal protein L32	64983
201846_s_at	0.0981	9.15E-03	-1.001556	RYBP	RING1 and YY1 binding protein	23429
200989_at	0.0981	9.16E-03	-1.4601087	HIF1A	hypoxia inducible factor 1 alpha subunit	3091
217769_s_at	0.0981	9.18E-03	-1.103822	POMP	proteasome maturation protein	51371
203259_s_at	0.0982	9.19E-03	-1.1875253	HDDC2	HD domain containing 2	51020
223492_s_at	0.0982	9.20E-03	-1.0089527	LRRFIP1	LRR binding FLII interacting protein 1	9208
1553987_at	0.0986	9.31E-03	-1.294786	MAPKAPK5-AS1	MAPKAPK5 antisense RNA 1	51275
201586_s_at	0.0986	9.32E-03	-1.2581573	SFPQ	splicing factor proline and glutamine rich	6421
212716_s_at	0.0986	9.33E-03	-1.5714647	EIF3K	eukaryotic translation initiation factor 3 subunit K	27335

201963_at	0.0986	9.34E-03	-1.51	ACSL1	acyl-CoA synthetase long-chain family member 1	2180
202605_at	0.0986	9.37E-03	-1.1011133	GUSB	glucuronidase beta	2990
228283_at	0.0988	9.42E-03	-1.3875247	CMC1	C-X9-C motif containing 1	152100
203462_x_at	0.0988	9.48E-03	-1.11441	EIF3B	eukaryotic translation initiation factor 3 subunit B	8662
202739_s_at	0.0988	9.49E-03	-1.0406647	PHKB	phosphorylase kinase regulatory subunit beta	5257
215088_s_at	0.0988	9.50E-03	-1.2312007	SDHC	succinate dehydrogenase complex subunit C	6391
201084_s_at	0.0989	9.52E-03	-1.242666	BCLAF1	BCL2 associated transcription factor 1	9774
225365_at	0.0989	9.53E-03	-1.2634253	ZDHHC20	zinc finger DHHC-type containing 20	253832

209295_at	0.0989	9.54E-03	-1.1297213	TNFRSF10B	TNF receptor superfamily member 10b	8795
224698_at	0.0989	9.55E-03	-1.206862	ESYT2	extended synaptotagmin 2	57488
200664_s_at	0.0989	9.55E-03	-1.009248	DNAJB1	DnaJ heat shock protein family (Hsp40) member B1	3337
223012_at	0.099	9.57E-03	-1.230034	MIR4746///UBXN6	microRNA 4746///UBX domain protein 6	100616371///80700
225852_at	0.0991	9.60E-03	-1.0564367	ANKRD17	ankyrin repeat domain 17	26057
212373_at	0.0991	9.60E-03	-1.1928727	FEM1B	fem-1 homolog B	10116
208774_at	0.0991	9.64E-03	-1.001658	CSNK1D	casein kinase 1 delta	1453
221596_s_at	0.0991	9.67E-03	-1.0632773	RBM48	RNA binding motif protein 48	84060
207723_s_at	0.0991	9.71E-03	-1.162008	KLRC3	killer cell lectin like receptor C3	3823

211317_s_at	0.0991	9.76E-03	-1.164484	CFLAR	CASP8 and FADD like apoptosis regulator	8837
213906_at	0.0991	9.76E-03	-1.203666	MYBL1	MYB proto-oncogene like 1	4603
222621_at	0.0991	9.76E-03	-1.0366613	DNAJC1	DnaJ heat shock protein family (Hsp40) member C1	64215
226425_at	0.0991	9.77E-03	-1.239366	CLIP4	CAP-Gly domain containing linker protein family member 4	79745
204516_at	0.0993	9.83E-03	-1.202056	ATXN7	ataxin 7	6314

209181_s_at	0.0995	9.87E-03	-1.2056107	SNORD45C///SNORD45A///SNORD45B///RABGGTB	small nucleolar RNA, C/D box 45C///small nucleolar RNA, C/D box 45A///small nucleolar RNA, C/D box 45B///Rab geranylgeranyltransferase beta subunit	692085///26805///26804///5876
229711_s_at	0.0995	9.87E-03	-1.0427013	MDM2	MDM2 proto-oncogene	4193
225310_at	0.0996	9.92E-03	-1.3441287	LOC101928747///RBMX///SNORD61	uncharacterized LOC101928747///RNA binding motif protein, X-linked///small nucleolar RNA, C/D box 61	101928747///27316///26787
217923_at	0.0996	9.92E-03	-1.0130087	PEF1	penta-EF-hand domain containing 1	553115

201227_s_at	0.0998	9.98E-03	-1.017352	NDUFB8	NADH:ubiquinone oxidoreductase subunit B8	4714
204805_s_at	0.0998	9.98E-03	-1.274532	H1FX	H1 histone family member X	8971
203704_s_at	0.0998	9.99E-03	-1.0925907	RREB1	ras responsive element binding protein 1	6239
200075_s_at	0.0999	1.00E-02	-1.431804	GUK1	guanylate kinase 1	2987
217168_s_at	0.0999	1.00E-02	-1.4039827	HERPUD1	homocysteine inducible ER protein with ubiquitin like domain 1	9709
223465_at	0.0999	1.00E-02	-1.1091	COL4A3BP	collagen type IV alpha 3 binding protein	10087
211752_s_at	0.1001	1.01E-02	-1.113258	NDUFS7	NADH:ubiquinone oxidoreductase core subunit S7	374291
210164_at	0.1001	1.01E-02	-1.3316973	GZMB	granzyme B	3002

208706_s_at	0.1002	1.01E-02	-1.08421	EIF5	eukaryotic translation initiation factor 5	1983
227110_at	0.1002	1.01E-02	-1.037366	HNRNPC	heterogeneous nuclear ribonucleoprotein C (C1/C2)	3183
235384_at	0.1003	1.02E-02	-1.1773947	NUDT19	nudix hydrolase 19	390916
218319_at	0.1003	1.02E-02	-1.244342	PELI1	pellino E3 ubiquitin protein ligase 1	57162
223294_at	0.1003	1.02E-02	-1.1373027	PBDC1	polysaccharide biosynthesis domain containing 1	51260
218929_at	0.1003	1.02E-02	-1.0048693	CDKN2AIP	CDKN2A interacting protein	55602
223043_at	0.1003	1.02E-02	-1.496244	EMC4	ER membrane protein complex subunit 4	51234
1560276_at	0.1003	1.02E-02	1.09703	C12orf80	chromosome 12 open reading frame 80	283403

201268_at	0.1003	1.02E-02	-1.394296	NME1-NME2///NME2	NME1-NME2 readthrough///NME/NM23 nucleoside diphosphate kinase 2	654364///4831
231973_s_at	0.1003	1.02E-02	-1.035128	LOC101930107///LOC730268///LOC285074///ANAPC1	uncharacterized LOC101930107///anaphase-promoting complex subunit 1-like///anaphase promoting complex subunit 1 pseudogene///anaphase promoting complex subunit 1	101930107///730268///285074///64682
221763_at	0.1004	1.02E-02	-1.2422467	JMJD1C	jumonji domain containing 1C	221037
211759_x_at	0.1005	1.03E-02	-1.203738	TBCB	tubulin folding cofactor B	1155
200921_s_at	0.1005	1.03E-02	-1.0133073	BTG1	BTG anti-proliferation factor 1	694

212036_s_at	0.1006	1.04E-02	-1.0159527	PNN	pinin, desmosome associated protein	5411
223218_s_at	0.1006	1.04E-02	-1.2457227	NFKBIZ	NFKB inhibitor zeta	64332
219759_at	0.1009	1.04E-02	-1.1888827	ERAP2	endoplasmic reticulum aminopeptidase 2	64167
224512_s_at	0.1009	1.04E-02	-1.1466367	NAA38	N(alpha)-acetyltransferase 38, NatC auxiliary subunit	84316
224665_at	0.1009	1.04E-02	-1.3732873	ANAPC16	anaphase promoting complex subunit 16	119504
205488_at	0.1009	1.05E-02	-1.7099853	GZMA	granzyme A	3001
209620_s_at	0.1009	1.05E-02	-1.06651	ABCB7	ATP binding cassette subfamily B member 7	22
205812_s_at	0.101	1.05E-02	-1.369346	TMED9	transmembrane p24 trafficking protein 9	54732

228582_x_at	0.1011	1.05E-02	2.6594953	MALAT1	metastasis associated lung adenocarcinoma transcript 1 (non-protein coding)	378938
213656_s_at	0.1011	1.06E-02	-1.0407133	KLC1	kinesin light chain 1	3831
219681_s_at	0.1011	1.06E-02	-1.393616	RAB11FIP1	RAB11 family interacting protein 1	80223
200837_at	0.1011	1.06E-02	-1.083392	BCAP31	B-cell receptor-associated protein 31	10134
221751_at	0.1011	1.06E-02	-1.1816693	PANK3	pantothenate kinase 3	79646
206015_s_at	0.1011	1.06E-02	-1.0666727	FOXJ3	forkhead box J3	22887
226771_at	0.1012	1.06E-02	-1.058754	ATP8B2	ATPase phospholipid transporting 8B2	57198
203380_x_at	0.1012	1.06E-02	-1.1220807	SRSF5	serine and arginine rich splicing factor 5	6430
212917_x_at	0.1012	1.06E-02	-1.059278	RECQL	RecQ like helicase	5965

208764_s_at	0.1014	1.07E-02	-1.285906	ATP5G2	ATP synthase, H+ transporting, mitochondrial Fo complex subunit C2 (subunit 9)	517
239014_at	0.1015	1.07E-02	-1.03274	CCAR1	cell division cycle and apoptosis regulator 1	55749
227020_at	0.1015	1.07E-02	-1.2127627	YPEL2	yippee like 2	388403
226965_at	0.1015	1.07E-02	-1.1138873	DENND6A	DENN domain containing 6A	201627
226296_s_at	0.1015	1.07E-02	-1.1100207	MRPS15	mitochondrial ribosomal protein S15	64960
202083_s_at	0.1017	1.08E-02	-1.0719293	SEC14L1	SEC14 like lipid binding 1	6397
206621_s_at	0.1017	1.08E-02	-1.1700373	EIF4H	eukaryotic translation initiation factor 4H	7458

227517_s_at	0.1017	1.08E-02	-1.1607247	SNORD77///SNORD76///SNORD74///GAS5///SNORD44///SNORD47///SNORD80///SNORD79///SNORD81	small nucleolar RNA, C/D box 77///small nucleolar RNA, C/D box 76///small nucleolar RNA, C/D box 74///growth arrest specific 5 (non-protein coding)///small nucleolar RNA, C/D box 44///small nucleolar RNA, C/D box 47///small nucleolar	692197///692196///619498///60674///26806///26802///26774///26770///26769
244008_at	0.1017	1.08E-02	-1.0024187	PARP8	poly(ADP-ribose) polymerase family member 8	79668
222423_at	0.1018	1.08E-02	-1.2204133	NDFIP1	Nedd4 family interacting protein 1	80762
225498_at	0.1018	1.08E-02	-1.3824067	CHMP4B	charged multivesicular body protein 4B	128866
211986_at	0.1018	1.08E-02	-1.1060927	AHNAK	AHNAK nucleoprotein	79026

206785_s_at	0.1018	1.09E-02	-1.643244	KLRC2///KLRC1	killer cell lectin like receptor C2///killer cell lectin like receptor C1	3822///3821
226336_at	0.1018	1.09E-02	-1.102256	LOC101060363///PPIA	peptidyl-prolyl cis-trans isomerase A pseudogene///peptidylprolyl isomerase A	101060363///5478
227062_at	0.1018	1.09E-02	-1.300864	MIR612///NEAT1	microRNA 612///nuclear paraspeckle assembly transcript 1 (non-protein coding)	693197///283131
222420_s_at	0.1018	1.09E-02	-1.2770073	UBE2H	ubiquitin conjugating enzyme E2 H	7328
201502_s_at	0.1018	1.09E-02	-1.03562	NFKBIA	NFKB inhibitor alpha	4792
215440_s_at	0.1018	1.09E-02	-1.2381227	BEX4	brain expressed X-linked 4	56271

203509_at	0.102	1.10E-02	-1.0214087	SORL1	sortilin related receptor 1	6653
202626_s_at	0.1023	1.11E-02	-1.3288033	LYN	LYN proto-oncogene, Src family tyrosine kinase	4067
228590_at	0.1023	1.11E-02	-1.0021333	PTCD3	pentatricopeptide repeat domain 3	55037
64432_at	0.1023	1.12E-02	-1.1341213	MAPKAPK5-AS1	MAPKAPK5 antisense RNA 1	51275
226625_at	0.1023	1.12E-02	-1.0592587	TGFBR3	transforming growth factor beta receptor 3	7049
225326_at	0.1023	1.12E-02	-1.0605947	RBM27	RNA binding motif protein 27	54439
228652_at	0.1023	1.12E-02	-1.0520127	ZNF776	zinc finger protein 776	284309
213399_x_at	0.1024	1.12E-02	-1.07116	RPN2	ribophorin II	6185
202929_s_at	0.1024	1.12E-02	-1.2645093	DDTL///DDT	D-dopachrome tautomerase-like///D-dopachrome tautomerase	100037417///1652

223105_s_at	0.1025	1.13E-02	-1.1748047	TMEM14B///TMEM14C	transmembrane protein 14B///transmembrane protein 14C	81853///51522
200936_at	0.1025	1.13E-02	-1.4348873	RPL8	ribosomal protein L8	6132
211936_at	0.1025	1.13E-02	-1.1851013	HSPA5	heat shock protein family A (Hsp70) member 5	3309
238035_at	0.1025	1.13E-02	-1.1067093	SP3	Sp3 transcription factor	6670
214709_s_at	0.1025	1.13E-02	-1.0805133	KTN1	kinectin 1	3895
202425_x_at	0.1029	1.15E-02	-1.0678153	PPP3CA	protein phosphatase 3 catalytic subunit alpha	5530
208319_s_at	0.1029	1.15E-02	-1.665166	RBM3	RNA binding motif (RNP1, RRM) protein 3	5935
202951_at	0.103	1.15E-02	-1.1770573	STK38	serine/threonine kinase 38	11329
232520_s_at	0.1031	1.15E-02	-1.0065707	NSFL1C	NSFL1 cofactor	55968

222826_at	0.1031	1.15E-02	-1.3768227	BLOC1S6	biogenesis of lysosomal organelles complex 1 subunit 6	26258
201916_s_at	0.1031	1.15E-02	-1.1646753	SEC63	SEC63 homolog, protein translocation regulator	11231
208306_x_at	0.1032	1.16E-02	-1.005286	HLA-DRB1	major histocompatibility complex, class II, DR beta 1	3123
218389_s_at	0.1032	1.16E-02	-1.0572393	APH1A	aph-1 homolog A, gamma-secretase subunit	51107
204093_at	0.1032	1.16E-02	-1.0542853	CCNH	cyclin H	902
228415_at	0.1032	1.16E-02	-1.23319	AP1S2	adaptor related protein complex 1 sigma 2 subunit	8905
235791_x_at	0.1033	1.17E-02	-1.350152	CHD1	chromodomain helicase DNA binding protein 1	1105

226290_at	0.1034	1.17E-02	-1.2611813	BDP1	B double prime 1, subunit of RNA polymerase III transcription initiation factor IIIB	55814
215498_s_at	0.1034	1.17E-02	-1.2125553	LOC100996792///MAP2K3	dual specificity mitogen-activated protein kinase kinase 3///mitogen-activated protein kinase kinase 3	100996792///5606
211931_s_at	0.1036	1.17E-02	-1.352692	HNRNPA3///HNRNPA3P1	heterogeneous nuclear ribonucleoprotein A3///heterogeneous nuclear ribonucleoprotein A3 pseudogene 1	220988///10151
201137_s_at	0.1036	1.17E-02	-1.254864	HLA-DPB1	major histocompatibility complex, class II, DP beta 1	3115

209472_at	0.1036	1.18E-02	-1.0735567	KYAT3	kynurenine aminotransferase 3	56267
203234_at	0.1036	1.18E-02	-1.047216	UPP1	uridine phosphorylase 1	7378
224502_s_at	0.1037	1.18E-02	-1.2833747	KIAA1191	KIAA1191	57179
217743_s_at	0.1038	1.18E-02	-1.053386	TMEM30A	transmembrane protein 30A	55754
226148_at	0.1039	1.19E-02	-1.113372	ZBTB44	zinc finger and BTB domain containing 44	29068
202171_at	0.1039	1.19E-02	-1.3739213	VEZF1	vascular endothelial zinc finger 1	7716
223006_s_at	0.1039	1.19E-02	-1.1056233	TMEM245	transmembrane protein 245	23731
213016_at	0.104	1.19E-02	-1.0506467	BBX	BBX, HMG-box containing	56987
218645_at	0.104	1.19E-02	-1.1630427	ZNF277	zinc finger protein 277	11179
219065_s_at	0.104	1.19E-02	-1.2774787	DPY30///MEMO1	dpy-30, histone methyltransferase complex regulatory subunit///mediator of cell motility 1	84661///51072

220939_s_at	0.104	1.19E-02	-1.0819067	DPP8	dipeptidyl peptidase 8	54878
217437_s_at	0.104	1.19E-02	-1.0435773	TACC1	transforming acidic coiled-coil containing protein 1	6867
221984_s_at	0.104	1.19E-02	-1.00023	FAM134A	family with sequence similarity 134 member A	79137
204061_at	0.1041	1.20E-02	-1.114236	PRKX	protein kinase, X-linked	5613
213824_at	0.1041	1.20E-02	1.0453727	OLIG2	oligodendrocyte lineage transcription factor 2	10215
222389_s_at	0.1042	1.20E-02	-1.24316	WAC	WW domain containing adaptor with coiled-coil	51322
222140_s_at	0.1046	1.21E-02	-1.031886	GPR89A///GPR89B	G protein-coupled receptor 89A///G protein-coupled receptor 89B	653519///51463

215091_s_at	0.1046	1.21E-02	-1.081228	GTF3A	general transcription factor IIIA	2971
212605_s_at	0.1046	1.22E-02	-1.4315573	NUDT3	nudix hydrolase 3	11165
218518_at	0.1046	1.22E-02	-1.3067273	FAM13B	family with sequence similarity 13 member B	51306
201092_at	0.1047	1.22E-02	-1.139692	RBBP7	RB binding protein 7, chromatin remodeling factor	5931
227577_at	0.105	1.23E-02	-1.184578	EXOC8	exocyst complex component 8	149371
1552621_at	0.105	1.23E-02	-1.150426	UPK3BL///POLR2J3///POLR2J2///POLR2J	uroplakin 3B-like///RNA polymerase II subunit J3///RNA polymerase II subunit J2///RNA polymerase II subunit J	100134938///548644///246721///5439
205214_at	0.105	1.23E-02	-1.3524113	STK17B	serine/threonine kinase 17b	9262

238974_at	0.1051	1.23E-02	-1.0310773	C2orf69	chromosome 2 open reading frame 69	205327
209025_s_at	0.1051	1.23E-02	-1.155892	SYNCRIP	synaptotagmin binding cytoplasmic RNA interacting protein	10492
238041_at	0.1051	1.23E-02	-1.0179673	TCF12	transcription factor 12	6938
219599_at	0.1052	1.24E-02	1.1635827	EIF4B	eukaryotic translation initiation factor 4B	1975
225222_at	0.1053	1.24E-02	-1.1182947	MFSD14A	major facilitator superfamily domain containing 14A	64645
218610_s_at	0.1053	1.24E-02	-1.5269813	CPPED1	calcineurin like phosphoesterase domain containing 1	55313
231870_s_at	0.1054	1.24E-02	-1.0593213	NMD3	NMD3 ribosome export adaptor	51068
200914_x_at	0.1054	1.25E-02	-1.073472	KTN1	kinectin 1	3895

218618_s_at	0.1054	1.25E-02	-1.072836	LOC101928615///FNDC3B	uncharacterized LOC101928615///fibronectin type III domain containing 3B	101928615///64778
200663_at	0.1055	1.25E-02	-1.2010353	CD63	CD63 molecule	967
1556035_s_at	0.1056	1.26E-02	-1.0468773	ZNF207	zinc finger protein 207	7756
222623_s_at	0.1058	1.26E-02	-1.1223633	ZNF639	zinc finger protein 639	51193
238013_at	0.1058	1.26E-02	-1.05715	PLEKHA2	pleckstrin homology domain containing A2	59339
227983_at	0.1059	1.27E-02	-1.038552	RILPL2	Rab interacting lysosomal protein like 2	196383
226006_at	0.106	1.27E-02	-1.372604	PET100	PET100 homolog	100131801
201565_s_at	0.1061	1.27E-02	-1.5733007	ID2	inhibitor of DNA binding 2, HLH protein	3398
230779_at	0.1061	1.27E-02	-1.3247587	TNRC6B	trinucleotide repeat containing 6B	23112
202863_at	0.1063	1.29E-02	-1.06863	SP100	SP100 nuclear antigen	6672

210840_s_at	0.1064	1.29E-02	-1.2020293	IQGAP1	IQ motif containing GTPase activating protein 1	8826
200662_s_at	0.1065	1.30E-02	-1.2045493	TOMM20	translocase of outer mitochondrial membrane 20	9804
227433_at	0.1065	1.30E-02	-1.148246	USF3	upstream transcription factor family member 3	205717
200904_at	0.1065	1.30E-02	-1.1399967	HLA-E	major histocompatibility complex, class I, E	3133
215223_s_at	0.1065	1.30E-02	-1.147042	LOC100129518///SOD2	uncharacterized LOC100129518///superoxide dismutase 2, mitochondrial	100129518///6648
207474_at	0.1066	1.30E-02	-1.0132613	SNRK	SNF related kinase	54861
218167_at	0.1066	1.30E-02	-1.201088	AMZ2	archaelysin family metallopeptidase 2	51321

202343_x_at	0.1066	1.30E-02	-1.3551027	COX5B	cytochrome c oxidase subunit 5B	1329
224573_at	0.1066	1.30E-02	-1.329122	RNASEK-C17orf49///RNASEK///C17orf49	RNASEK-C17orf49 readthrough///ribonuclease K///chromosome 17 open reading frame 49	100529209///440400///124944
209318_x_at	0.1068	1.31E-02	-1.0262153	PLAGL1	PLAG1 like zinc finger 1	5325
226320_at	0.1068	1.31E-02	-1.0709427	ALYREF	Aly/REF export factor	10189
208933_s_at	0.1068	1.31E-02	-1.0898627	LGALS8	galectin 8	3964
218100_s_at	0.1071	1.32E-02	-1.091346	IFT57	intraflagellar transport 57	55081
209606_at	0.1071	1.32E-02	-1.0977833	CYTIP	cytohesin 1 interacting protein	9595
208836_at	0.1071	1.33E-02	-1.135138	ATP1B3	ATPase Na+/K+ transporting subunit beta 3	483
218668_s_at	0.1071	1.33E-02	-1.2056627	RAP2C	RAP2C, member of RAS oncogene family	57826
200977_s_at	0.1071	1.33E-02	-1.2793267	TAX1BP1	Tax1 binding protein 1	8887

227068_at	0.1071	1.33E-02	-1.2234173	PGK1	phosphoglycerate kinase 1	5230
228745_at	0.1071	1.33E-02	-1.1900933	SGTB	small glutamine rich tetratricopeptide repeat containing beta	54557
222488_s_at	0.1071	1.33E-02	-1.244062	DCTN4	dynactin subunit 4	51164
225213_at	0.1071	1.33E-02	-1.199372	PPTC7	PTC7 protein phosphatase homolog	160760
224811_at	0.1071	1.33E-02	-1.022842	LPP	LIM domain containing preferred translocation partner in lipoma	4026
202803_s_at	0.1072	1.34E-02	-1.2828407	ITGB2	integrin subunit beta 2	3689
228543_at	0.1073	1.34E-02	-1.047172	PET117	PET117 homolog	100303755
217870_s_at	0.1073	1.35E-02	-1.22304	CMPK1	cytidine/uridine monophosphate kinase 1	51727
1567214_a_at	0.1073	1.35E-02	-1.0655473	PNN	pinin, desmosome associated protein	5411
205081_at	0.1073	1.35E-02	-1.2152607	CRIP1	cysteine rich protein 1	1396

212265_at	0.1074	1.35E-02	-1.25956	QKI	QKI, KH domain containing RNA binding	9444
217871_s_at	0.1075	1.35E-02	-1.467408	MIF	macrophage migration inhibitory factor (glycosylation-inhibiting factor)	4282
211929_at	0.1076	1.35E-02	-1.0441207	HNRNPA3	heterogeneous nuclear ribonucleoprotein A3	220988
201595_s_at	0.1076	1.35E-02	-1.2234473	ZC3H15	zinc finger CCCH-type containing 15	55854
228097_at	0.1076	1.36E-02	-1.38681	MYLIP	myosin regulatory light chain interacting protein	29116
213376_at	0.1076	1.36E-02	-1.29119	ZBTB1	zinc finger and BTB domain containing 1	22890

210891_s_at	0.1077	1.37E-02	-1.112992	GTF2IP4///GTF2IP1///GTF2I	general transcription factor IIi pseudogene 4///general transcription factor IIi pseudogene 1///general transcription factor IIi	100093631///2970///2969
222163_s_at	0.1078	1.37E-02	-1.03412	SPATA5L1	spermatogenesis associated 5 like 1	79029
228793_at	0.1078	1.37E-02	-1.1065053	JMJD1C	jumonji domain containing 1C	221037
217840_at	0.1078	1.37E-02	-1.096786	DDX41	DEAD-box helicase 41	51428
211998_at	0.108	1.37E-02	-1.1509507	MIR4738///H3F3B///H3F3A	microRNA 4738///H3 histone, family 3B///H3 histone, family 3A	100616282///3021///3020
208647_at	0.1081	1.37E-02	-1.0433587	FDFT1	farnesyl-diphosphate farnesyltransferase 1	2222
227478_at	0.1081	1.38E-02	-1.0413553	SETBP1	SET binding protein 1	26040

216237_s_at	0.1081	1.38E-02	-1.2115593	MCM5	minichromosome maintenance complex component 5	4174
201083_s_at	0.1082	1.38E-02	-1.2204707	BCLAF1	BCL2 associated transcription factor 1	9774
200600_at	0.1082	1.38E-02	-1.4501267	MSN	moesin	4478
205241_at	0.1082	1.38E-02	-1.139898	SCO2	SCO2 cytochrome c oxidase assembly protein	9997
204912_at	0.1082	1.39E-02	-1.0469793	IL10RA	interleukin 10 receptor subunit alpha	3587
213846_at	0.1083	1.39E-02	-1.160908	COX7C	cytochrome c oxidase subunit 7C	1350

221580_s_at	0.1085	1.39E-02	-1.0375567	MIR1304///SNORD5///SNORA32///SNORA40///SNORA18///SNORA1///SNORA8///TAF1D	microRNA 1304///small nucleolar RNA, C/D box 5///small nucleolar RNA, H/ACA box 32///small nucleolar RNA, H/ACA box 40///small nucleolar RNA, H/ACA box 18///small nucleolar RNA, H/ACA box 1///small nucleolar	100302240///692072///692063///677822///677805///677792///654320///79101
200900_s_at	0.1085	1.39E-02	-1.2065647	M6PR	mannose-6-phosphate receptor, cation dependent	4074
227095_at	0.1085	1.39E-02	-1.324072	LEPROT	leptin receptor overlapping transcript	54741
226134_s_at	0.1087	1.40E-02	-1.125694	MSI2	musashi RNA binding protein 2	124540

220330_s_at	0.1087	1.40E-02	-1.5084187	SAMSN1	SAM domain, SH3 domain and nuclear localization signals 1	64092
203113_s_at	0.1087	1.40E-02	-1.1797107	EEF1D	eukaryotic translation elongation factor 1 delta	1936
207571_x_at	0.1087	1.40E-02	-1.2718067	THEMIS2	thymocyte selection associated family member 2	9473
212708_at	0.1087	1.40E-02	-1.1202147	MSL1	male specific lethal 1 homolog	339287
223051_at	0.1088	1.41E-02	-1.0588713	SSU72	SSU72 homolog, RNA polymerase II CTD phosphatase	29101
209323_at	0.1088	1.41E-02	-1.2531607	THAP12	THAP domain containing 12	5612
223416_at	0.1088	1.41E-02	-1.0976087	SF3B6	splicing factor 3b subunit 6	51639

215171_s_at	0.1088	1.41E-02	-1.1365913	TIMM17A	translocase of inner mitochondrial membrane 17 homolog A (yeast)	10440
224981_at	0.109	1.42E-02	-1.2350127	TMEM219	transmembrane protein 219	124446
219293_s_at	0.1093	1.43E-02	-1.0208913	OLA1	Obg-like ATPase 1	29789
201464_x_at	0.1093	1.43E-02	-1.1483453	JUN	Jun proto-oncogene, AP-1 transcription factor subunit	3725
213359_at	0.1093	1.43E-02	-1.10567	HNRNPD	heterogeneous nuclear ribonucleoprotein D	3184
202333_s_at	0.1093	1.43E-02	-1.2057767	UBE2B	ubiquitin conjugating enzyme E2 B	7320
225073_at	0.1093	1.43E-02	-1.2289813	PPHLN1	periphilin 1	51535
208612_at	0.1095	1.43E-02	-1.3184873	PDIA3	protein disulfide isomerase family A member 3	2923

212766_s_at	0.1097	1.44E-02	-1.0707407	ISG20L2	interferon stimulated exonuclease gene 20 like 2	81875
226496_at	0.1097	1.44E-02	-1.139596	ZCCHC7	zinc finger CCHC-type containing 7	84186
1555526_a_at	0.1097	1.45E-02	-1.158676	SEPT6	septin 6	23157
211698_at	0.1098	1.45E-02	-1.2431793	EID1	EP300 interacting inhibitor of differentiation 1	23741
203531_at	0.1098	1.45E-02	-1.033496	CUL5	cullin 5	8065
225932_s_at	0.1099	1.46E-02	-1.0795213	HNRNPA2B1	heterogeneous nuclear ribonucleoprotein A2/B1	3181
208652_at	0.1099	1.46E-02	-1.3308747	PPP2CA	protein phosphatase 2 catalytic subunit alpha	5515
213549_at	0.11	1.47E-02	-1.1120213	PDZD8	PDZ domain containing 8	118987
224606_at	0.11	1.47E-02	-1.11634	KLF6	Kruppel like factor 6	1316
212209_at	0.1101	1.47E-02	-1.0261147	MED13L	mediator complex subunit 13 like	23389

224934_at	0.1102	1.47E-02	-1.006644	YIPF5	Yip1 domain family member 5	81555
204198_s_at	0.1102	1.47E-02	-1.0348453	RUNX3	runt related transcription factor 3	864
223176_at	0.1102	1.48E-02	-1.3112673	KCTD20	potassium channel tetramerization domain containing 20	222658
201285_at	0.1102	1.48E-02	-1.0605153	MKRN1	makorin ring finger protein 1	23608
204279_at	0.1102	1.48E-02	-1.4253	PSMB9	proteasome subunit beta 9	5698
201300_s_at	0.1102	1.48E-02	-1.33758	PRNP	prion protein	5621
1559067_a_at	0.1102	1.48E-02	-1.1967353	LOC158402	uncharacterized LOC158402	158402
200005_at	0.1104	1.49E-02	-1.206706	EIF3D	eukaryotic translation initiation factor 3 subunit D	8664
224579_at	0.1104	1.49E-02	-1.1093793	SLC38A1	solute carrier family 38 member 1	81539

200087_s_at	0.1104	1.49E-02	-1.1797513	TMED2	transmembrane p24 trafficking protein 2	10959
225547_at	0.1104	1.49E-02	-1.072334	SNORD87///SNHG6	small nucleolar RNA, C/D box 87///small nucleolar RNA host gene 6	641648///641638
212927_at	0.1104	1.49E-02	-1.101426	SMC5	structural maintenance of chromosomes 5	23137
224800_at	0.1105	1.50E-02	-1.08825	WDFY1	WD repeat and FYVE domain containing 1	57590
201077_s_at	0.1106	1.50E-02	-1.109448	SNU13///ANXA2	SNU13 homolog, small nuclear ribonucleoprotein (U4/U6.U5)///annexin A2	4809///302
212266_s_at	0.1108	1.51E-02	-1.1979613	SRSF5	serine and arginine rich splicing factor 5	6430
201197_at	0.1108	1.51E-02	-1.0441647	AMD1	adenosylmethionine decarboxylase 1	262

217497_at	0.1108	1.52E-02	-1.2649927	TYMP	thymidine phosphorylase	1890
203232_s_at	0.1108	1.52E-02	-1.0213647	ATXN1	ataxin 1	6310
201466_s_at	0.1112	1.53E-02	-1.343972	JUN	Jun proto-oncogene, AP-1 transcription factor subunit	3725
224761_at	0.1113	1.53E-02	-1.0976813	GNA13	G protein subunit alpha 13	10672
225413_at	0.1113	1.53E-02	-1.3578173	USMG5	up-regulated during skeletal muscle growth 5 homolog (mouse)	84833
223070_at	0.1114	1.54E-02	-1.035832	SELK	selenoprotein K	58515
200814_at	0.1114	1.54E-02	-1.2352047	PSME1	proteasome activator subunit 1	5720
201925_s_at	0.1114	1.55E-02	-1.20574	CD55	CD55 molecule (Cromer blood group)	1604
225814_at	0.1116	1.55E-02	-1.0965633	XRN1	5'-3' exoribonuclease 1	54464
211678_s_at	0.1118	1.56E-02	-1.0868853	RNF114	ring finger protein 114	55905

215838_at	0.1118	1.56E-02	-1.3682687	LILRA5	leukocyte immunoglobulin like receptor A5	353514
225797_at	0.1118	1.56E-02	-1.0416133	MRPL54	mitochondrial ribosomal protein L54	116541
226370_at	0.1118	1.57E-02	-1.1294853	KLHL15	kelch like family member 15	80311
218053_at	0.1119	1.57E-02	-1.1984327	PRPF40A	pre-mRNA processing factor 40 homolog A	55660
228569_at	0.1119	1.57E-02	-1.1743487	PAPOLA	poly(A) polymerase alpha	10914
201552_at	0.112	1.57E-02	-1.658514	LAMP1	lysosomal associated membrane protein 1	3916
209083_at	0.1121	1.58E-02	-1.2363447	CORO1A	coronin 1A	11151
203166_at	0.1121	1.58E-02	-1.1169787	CFDP1	craniofacial development protein 1	10428
210908_s_at	0.1122	1.58E-02	-1.3095987	PFDN5	prefoldin subunit 5	5204

206542_s_at	0.1123	1.59E-02	-1.318386	SMARCA2	SWI/SNF related, matrix associated, actin dependent regulator of chromatin, subfamily a, member 2	6595
212103_at	0.1123	1.59E-02	-1.1511393	KPNA6	karyopherin subunit alpha 6	23633
212519_at	0.1123	1.59E-02	-1.5545573	UBE2E1	ubiquitin conjugating enzyme E2 E1	7324
210785_s_at	0.1124	1.59E-02	-1.2733213	THEMIS2	thymocyte selection associated family member 2	9473
212959_s_at	0.1124	1.59E-02	-1.160364	GNPTAB	N-acetylglucosamine-1-phosphate transferase alpha and beta subunits	79158

204286_s_at	0.1124	1.60E-02	-1.11612	PMAIP1	phorbol-12-myristate-13-acetate-induced protein 1	5366
200629_at	0.1127	1.61E-02	-1.0036587	WARS	tryptophanyl-tRNA synthetase	7453
208737_at	0.1129	1.62E-02	-1.237008	ATP6V1G1	ATPase H+ transporting V1 subunit G1	9550
210460_s_at	0.1129	1.62E-02	-1.151292	PSMD4	proteasome 26S subunit, non-ATPase 4	5710
212982_at	0.1129	1.62E-02	-1.2922173	ZDHHC17	zinc finger DHHC-type containing 17	23390
224939_at	0.1129	1.62E-02	-1.0081547	NUFIP2	NUFIP2, FMR1 interacting protein 2	57532
224593_at	0.1129	1.62E-02	-1.161446	ZNF664	zinc finger protein 664	144348
229460_at	0.1129	1.62E-02	-1.3043487	FAM126B	family with sequence similarity 126 member B	285172

200598_s_at	0.113	1.63E-02	-1.231312	MIR3652///HSP90B1	microRNA 3652///heat shock protein 90 beta family member 1	100500842///7184
213579_s_at	0.113	1.63E-02	-1.1447593	EP300	E1A binding protein p300	2033
223322_at	0.1131	1.63E-02	-1.0882953	RASSF5	Ras association domain family member 5	83593
226165_at	0.1131	1.63E-02	-1.2724067	C8orf59	chromosome 8 open reading frame 59	401466
235086_at	0.1131	1.64E-02	-1.1009253	THBS1	thrombospondin 1	7057
200945_s_at	0.1131	1.64E-02	-1.1802907	SEC31A	SEC31 homolog A, COPII coat complex component	22872
200642_at	0.1131	1.64E-02	-1.164272	SOD1	superoxide dismutase 1, soluble	6647
228697_at	0.1131	1.64E-02	-1.189422	HINT3	histidine triad nucleotide binding protein 3	135114

206036_s_at	0.1131	1.64E-02	-1.1525587	REL	REL proto-oncogene, NF-kB subunit	5966
212426_s_at	0.1133	1.64E-02	-1.224064	YWHAQ	tyrosine 3-monooxygenase/tryptophan 5-monooxygenase activation protein theta	10971
225105_at	0.1134	1.65E-02	-1.1519027	C12orf75	chromosome 12 open reading frame 75	387882
204417_at	0.1134	1.65E-02	-1.1923107	GALC	galactosylceramidase	2581
201392_s_at	0.1134	1.65E-02	-1.2643407	IGF2R	insulin like growth factor 2 receptor	3482
228056_s_at	0.1136	1.66E-02	-1.0664133	NAPSB	napsin B aspartic peptidase, pseudogene	256236
229390_at	0.1137	1.67E-02	-1.4123653	FAM26F	family with sequence similarity 26 member F	441168
209510_at	0.1138	1.67E-02	-1.3028547	RNF139	ring finger protein 139	11236

204070_at	0.1139	1.68E-02	-1.1181047	RARRES3	retinoic acid receptor responder 3	5920
241370_at	0.1139	1.68E-02	-1.0302753	LOC286052	uncharacterized LOC286052	286052
207002_s_at	0.1141	1.69E-02	-1.0425487	PLAGL1	PLAG1 like zinc finger 1	5325
215332_s_at	0.1141	1.69E-02	-1.0263667	LOC100996919///CD8B	putative T-cell surface glycoprotein CD8 beta-2 chain-like///CD8b molecule	100996919///926
208066_s_at	0.1142	1.70E-02	-1.0892153	GTF2B	general transcription factor IIB	2959
202971_s_at	0.1142	1.70E-02	-1.182426	DYRK2	dual specificity tyrosine phosphorylation regulated kinase 2	8445
230748_at	0.1144	1.71E-02	-1.1550833	SLC16A6	solute carrier family 16 member 6	9120
235919_at	0.1144	1.71E-02	-1.0697007	CEP78	centrosomal protein 78	84131

201757_at	0.1144	1.71E-02	-1.1282713	RPL10///NDUFS5	ribosomal protein L10///NADH:ubiquinone oxidoreductase subunit S5	6134///4725
221727_at	0.1145	1.72E-02	-1.01667	SUB1	SUB1 homolog, transcriptional regulator	10923
221263_s_at	0.1146	1.72E-02	-1.3094953	SF3B5	splicing factor 3b subunit 5	83443
1554676_at	0.1146	1.72E-02	-1.2305047	SRGN	serglycin	5552
211623_s_at	0.1146	1.72E-02	-1.3026333	FBL	fibrillarin	2091
202001_s_at	0.1148	1.73E-02	-1.025602	NDUFA6	NADH:ubiquinone oxidoreductase subunit A6	4700
229043_at	0.1149	1.73E-02	-1.2093227	PAPD5	poly(A) RNA polymerase D5, non-canonical	64282
218082_s_at	0.1151	1.74E-02	-1.0443667	UBP1	upstream binding protein 1 (LBP-1a)	7342
200728_at	0.1151	1.74E-02	-1.3183953	ACTR2	ARP2 actin related protein 2 homolog	10097

213416_at	0.1151	1.74E-02	-1.1471	ITGA4	integrin subunit alpha 4	3676
207081_s_at	0.1151	1.74E-02	-1.078712	PI4KA	phosphatidylinositol 4-kinase alpha	5297
218101_s_at	0.1151	1.75E-02	-1.335606	NDUFC2-KCTD14///NDUFC2	NDUFC2-KCTD14 readthrough///NADH:ubiquinone oxidoreductase subunit C2	100532726///4718
209377_s_at	0.1151	1.75E-02	-1.2102773	HMGN3	high mobility group nucleosomal binding domain 3	9324
219449_s_at	0.1151	1.75E-02	-1.1790507	TMEM70	transmembrane protein 70	54968
200748_s_at	0.1153	1.76E-02	-1.0498947	FTH1	ferritin heavy chain 1	2495
215633_x_at	0.1153	1.76E-02	-1.2147147	LST1	leukocyte specific transcript 1	7940
227309_at	0.1153	1.76E-02	-1.143308	YOD1	YOD1 deubiquitinase	55432
211932_at	0.1154	1.76E-02	-1.2499147	HNRNPA3	heterogeneous nuclear ribonucleoprotein A3	220988

235798_at	0.1154	1.77E-02	-1.3333087	TMEM170B	transmembrane protein 170B	100113407
225519_at	0.1154	1.77E-02	-1.0426713	PPP4R2	protein phosphatase 4 regulatory subunit 2	151987
209059_s_at	0.1154	1.77E-02	-1.3944747	EDF1	endothelial differentiation related factor 1	8721
217941_s_at	0.1154	1.77E-02	-1.2941553	ERBIN	erbb2 interacting protein	55914
208949_s_at	0.1155	1.77E-02	-1.244074	LGALS3	lectin, galactoside binding soluble 3	3958
221494_x_at	0.1156	1.78E-02	-1.1086187	EIF3K	eukaryotic translation initiation factor 3 subunit K	27335
212085_at	0.1156	1.78E-02	-1.7692333	SLC25A6	solute carrier family 25 member 6	293
214501_s_at	0.1157	1.79E-02	-1.0781733	H2AFY	H2A histone family member Y	9555
221675_s_at	0.1157	1.79E-02	-1.1792387	CHPT1	choline phosphotransferase 1	56994

204490_s_at	0.1157	1.79E-02	-1.3031927	CD44	CD44 molecule (Indian blood group)	960
208092_s_at	0.1158	1.79E-02	-1.0453353	FAM49A	family with sequence similarity 49 member A	81553
217900_at	0.1158	1.79E-02	-1.07437	IARS2	isoleucyl-tRNA synthetase 2, mitochondrial	55699
212508_at	0.1158	1.80E-02	-1.209512	MOAP1	modulator of apoptosis 1	64112
212070_at	0.1159	1.80E-02	-1.140766	ADGRG1	adhesion G protein-coupled receptor G1	9289
223067_at	0.1159	1.80E-02	-1.045794	CWC15	CWC15 spliceosome-associated protein	51503
213225_at	0.1159	1.80E-02	-1.044778	PPM1B	protein phosphatase, Mg2+/Mn2+ dependent 1B	5495
201105_at	0.116	1.81E-02	-1.449132	LGALS1	galectin 1	3956

222467_s_at	0.116	1.81E-02	-1.2400973	PPP6R3	protein phosphatase 6 regulatory subunit 3	55291
212706_at	0.1161	1.81E-02	-1.2489787	LOC102724229///RASA4B///RASA4	uncharacterized LOC102724229///RAS p21 protein activator 4B///RAS p21 protein activator 4	102724229///100271927///10156
200046_at	0.1162	1.81E-02	-1.3524233	DAD1	defender against cell death 1	1603
200658_s_at	0.1163	1.82E-02	-1.1603833	PHB	prohibitin	5245
200915_x_at	0.1163	1.82E-02	-1.0013727	KTN1	kinectin 1	3895
1552354_at	0.1164	1.82E-02	1.077272	CBARP	CACN beta subunit associated regulatory protein	255057
225284_at	0.1167	1.84E-02	-1.316572	DNAJC3	DnaJ heat shock protein family (Hsp40) member C3	5611

226753_at	0.1169	1.85E-02	-1.11015	FAM76B	family with sequence similarity 76 member B	143684
1555948_s_at	0.117	1.85E-02	-1.2088313	FAM120A	family with sequence similarity 120A	23196
212513_s_at	0.117	1.86E-02	-1.1222427	USP33	ubiquitin specific peptidase 33	23032
205711_x_at	0.117	1.86E-02	-1.054794	ATP5C1	ATP synthase, H+ transporting, mitochondrial F1 complex, gamma polypeptide 1	509
227964_at	0.1171	1.86E-02	-1.003176	FRMD8	FERM domain containing 8	83786
224651_at	0.1172	1.86E-02	-1.288064	CCNY	cyclin Y	219771
218924_s_at	0.1172	1.87E-02	-1.0692193	CTBS	chitobiase	1486
202731_at	0.1173	1.87E-02	-1.2161833	MIR4680///PDCD4	microRNA 4680///programmed cell death 4 (neoplastic transformation inhibitor)	100616113///27250

202396_at	0.1173	1.87E-02	-1.0534687	TCERG1	transcription elongation regulator 1	10915
209969_s_at	0.1174	1.88E-02	-1.0125453	STAT1	signal transducer and activator of transcription 1	6772
222412_s_at	0.1174	1.88E-02	-1.1975907	SSR3	signal sequence receptor subunit 3	6747
208905_at	0.1174	1.88E-02	-1.1854053	CYCS	cytochrome c, somatic	54205
230179_at	0.1174	1.88E-02	-1.0138927	LOC285812	uncharacterized LOC285812	285812
229287_at	0.1175	1.88E-02	-1.01738	PCNX1	pecanex homolog 1 (Drosophila)	22990
202442_at	0.1176	1.89E-02	-1.1136333	AP3S1	adaptor related protein complex 3 sigma 1 subunit	1176
207957_s_at	0.1176	1.89E-02	-1.0077673	PRKCB	protein kinase C beta	5579
200072_s_at	0.1176	1.90E-02	-1.0516167	HNRNPM	heterogeneous nuclear ribonucleoprotein M	4670

204006_s_at	0.1176	1.90E-02	-1.3453547	FCGR3B///FCGR3A	Fc fragment of IgG receptor IIIb///Fc fragment of IgG receptor IIIa	2215///2214
238668_at	0.1176	1.90E-02	-1.087528	NCKAP1L	NCK associated protein 1 like	3071
202817_s_at	0.1176	1.90E-02	-1.1895613	SS18	SS18, nBAF chromatin remodeling complex subunit	6760
225541_at	0.1176	1.90E-02	-1.262882	RPL22L1	ribosomal protein L22 like 1	200916
201488_x_at	0.1176	1.90E-02	-1.0879653	KHDRBS1	KH RNA binding domain containing, signal transduction associated 1	10657
200733_s_at	0.1179	1.91E-02	-1.1067113	PTP4A1	protein tyrosine phosphatase type IVA, member 1	7803
201887_at	0.1179	1.91E-02	-1.202828	IL13RA1	interleukin 13 receptor subunit alpha 1	3597

242131_at	0.1179	1.91E-02	1.0971553	ATP6	ATP synthase F0 subunit 6	4508
235276_at	0.118	1.92E-02	-1.0354133	EPSTI1	epithelial stromal interaction 1 (breast)	94240
208959_s_at	0.118	1.92E-02	-1.104264	ERP44	endoplasmic reticulum protein 44	23071
201393_s_at	0.118	1.92E-02	-1.1949327	IGF2R	insulin like growth factor 2 receptor	3482
231718_at	0.118	1.92E-02	-1.0697687	SLU7	SLU7 homolog, splicing factor	10569
217983_s_at	0.1181	1.92E-02	-1.0558193	RNASET2	ribonuclease T2	8635
212082_s_at	0.1181	1.93E-02	-1.0983367	MYL6	myosin light chain 6	4637
1558699_a_at	0.1182	1.93E-02	-1.3571133	HERPUD2	HERPUD family member 2	64224
211009_s_at	0.1182	1.93E-02	-1.0489653	ZNF271P	zinc finger protein 271, pseudogene	10778
202130_at	0.1182	1.93E-02	-1.127078	RIOK3	RIO kinase 3	8780
223049_at	0.1184	1.94E-02	-1.1495313	GRB2	growth factor receptor bound protein 2	2885

200052_s_at	0.1185	1.95E-02	-1.186946	ILF2	interleukin enhancer binding factor 2	3608
200071_at	0.1185	1.95E-02	-1.0467207	SMNDC1	survival motor neuron domain containing 1	10285
223370_at	0.1185	1.95E-02	-1.1949733	PLEKHA3	pleckstrin homology domain containing A3	65977
211582_x_at	0.1186	1.95E-02	-1.3990687	LST1	leukocyte specific transcript 1	7940
224575_at	0.1186	1.96E-02	-1.2483347	BRK1	BRICK1, SCAR/WAVE actin nucleating complex subunit	55845
209340_at	0.1187	1.96E-02	-1.12564	UAP1	UDP-N-acetylglucosamine pyrophosphorylase 1	6675
204640_s_at	0.1188	1.96E-02	-1.11491	SPOP	speckle type BTB/POZ protein	8405
212248_at	0.1188	1.97E-02	-1.1213547	MTDH	metadherin	92140
218428_s_at	0.1188	1.97E-02	-1.011468	REV1	REV1, DNA directed polymerase	51455

208675_s_at	0.1188	1.97E-02	-1.1811287	DDOST	dolichyl-diphosphooligosaccharide--protein glycosyltransferase non-catalytic subunit	1650
224810_s_at	0.1188	1.97E-02	-1.020168	ANKRD13A	ankyrin repeat domain 13A	88455
210835_s_at	0.1188	1.97E-02	-1.244108	CTBP2	C-terminal binding protein 2	1488
223982_s_at	0.1188	1.97E-02	-1.2622087	PNPLA8	patatin like phospholipase domain containing 8	50640
222975_s_at	0.1188	1.97E-02	-1.3495633	CSDE1	cold shock domain containing E1	7812
221760_at	0.1188	1.97E-02	-1.2668827	MAN1A1	mannosidase alpha class 1A member 1	4121
213902_at	0.1188	1.97E-02	-1.0834667	ASAH1	N-acylsphingosine amidohydrolase 1	427

235102_x_at	0.1188	1.98E-02	-1.1438807	SNORD3D///SNORD3C///SNORD3B-2///SNORD3A///SNORD3B-1	small nucleolar RNA, C/D box 3D///small nucleolar RNA, C/D box 3C///small nucleolar RNA, C/D box 3B-2///small nucleolar RNA, C/D box 3A///small nucleolar RNA, C/D box 3B-1	780854///780853///780852///780851///26851
219099_at	0.1189	1.98E-02	-1.0167293	TIGAR	TP53 induced glycolysis regulatory phosphatase	57103
201091_s_at	0.119	1.99E-02	-1.16717	CBX3	chromobox 3	11335
224777_s_at	0.1191	2.00E-02	-1.0239227	PAFAH1B2	platelet activating factor acetylhydrolase 1b catalytic subunit 2	5049

202469_s_at	0.1191	2.00E-02	-1.2848993	CPSF6	cleavage and polyadenylation specific factor 6	11052
200028_s_at	0.1191	2.00E-02	-1.00947	STARD7	StAR related lipid transfer domain containing 7	56910
225665_at	0.1191	2.00E-02	-1.0644087	ZAK	sterile alpha motif and leucine zipper containing kinase AZK	51776
202623_at	0.1191	2.00E-02	-1.3171933	EAPP	E2F associated phosphoprotein	55837
208877_at	0.1192	2.00E-02	-1.255512	PAK2	p21 (RAC1) activated kinase 2	5062
1560587_s_at	0.1192	2.01E-02	-1.8578193	PRDX5	peroxiredoxin 5	25824
225523_at	0.1193	2.01E-02	-1.0581733	MRPL53///CCDC142	mitochondrial ribosomal protein L53///coiled-coil domain containing 142	116540///84865

202541_at	0.1193	2.01E-02	-1.0402873	AIMP1	aminoacyl tRNA synthetase complex interacting multifunctional protein 1	9255
218679_s_at	0.1193	2.01E-02	-1.0790087	VPS28	VPS28, ESCRT-I subunit	51160
204224_s_at	0.1193	2.01E-02	-1.4214133	GCH1	GTP cyclohydrolase 1	2643
211503_s_at	0.1193	2.01E-02	-1.0756647	RAB14	RAB14, member RAS oncogene family	51552
202295_s_at	0.1194	2.02E-02	-1.0140927	CTSH	cathepsin H	1512
224374_s_at	0.1194	2.02E-02	-1.1312433	EMILIN2	elastin microfibril interfacer 2	84034
200675_at	0.1194	2.02E-02	-1.243644	CD81	CD81 molecule	975
213160_at	0.1195	2.02E-02	-1.0741553	DOCK2	dedicator of cytokinesis 2	1794
223577_x_at	0.1195	2.02E-02	1.3229333	MALAT1	metastasis associated lung adenocarcinoma transcript 1 (non-protein coding)	378938

220646_s_at	0.1195	2.03E-02	-1.5280707	KLRF1	killer cell lectin like receptor F1	51348
201862_s_at	0.1196	2.03E-02	-1.565352	LRRFIP1	LRR binding FLII interacting protein 1	9208
208734_x_at	0.1197	2.03E-02	-1.311468	RAB2A	RAB2A, member RAS oncogene family	5862
201304_at	0.1197	2.03E-02	-1.1580973	NDUFA5	NADH:ubiquinone oxidoreductase subunit A5	4698
218050_at	0.1198	2.04E-02	-1.026766	UFM1	ubiquitin fold modifier 1	51569
202233_s_at	0.1201	2.05E-02	-1.272504	UQCRHL///UQCRH	ubiquinol-cytochrome c reductase hinge protein like///ubiquinol-cytochrome c reductase hinge protein	440567///7388
201738_at	0.1201	2.06E-02	-1.1005933	EIF1B	eukaryotic translation initiation factor 1B	10289

212014_x_at	0.1203	2.06E-02	-1.026658	CD44	CD44 molecule (Indian blood group)	960
212893_at	0.1203	2.07E-02	-1.0277753	ZZZ3	zinc finger ZZ-type containing 3	26009
226177_at	0.1203	2.07E-02	-1.0831313	GLTP	glycolipid transfer protein	51228
203973_s_at	0.1204	2.07E-02	-1.3068313	CEBPD	CCAAT/enhancer binding protein delta	1052
200040_at	0.1205	2.08E-02	-1.0315233	KHDRBS1	KH RNA binding domain containing, signal transduction associated 1	10657
225318_at	0.1205	2.08E-02	-1.0110273	WHSC1L1	Wolf-Hirschhorn syndrome candidate 1-like 1	54904
212160_at	0.1205	2.08E-02	-1.1331727	XPOT	exportin for tRNA	11260
217738_at	0.1205	2.08E-02	-1.4169927	NAMPT	nicotinamide phosphoribosyltransferase	10135
214765_s_at	0.1205	2.08E-02	-1.119636	NAAA	N-acylethanolamine acid amidase	27163

207573_x_at	0.1207	2.09E-02	-1.266104	ATP5L	ATP synthase, H+ transporting, mitochondrial Fo complex subunit G	10632
202798_at	0.1207	2.09E-02	-1.117248	SEC24B	SEC24 homolog B, COPII coat complex component	10427
201231_s_at	0.1208	2.09E-02	-1.0666407	ENO1	enolase 1	2023
201160_s_at	0.1208	2.09E-02	-1.228852	YBX3	Y-box binding protein 3	8531
218150_at	0.1208	2.09E-02	-1.3705227	LOC101929356///ARL5A	uncharacterized LOC101929356///ADP ribosylation factor like GTPase 5A	101929356///26225
222552_at	0.1209	2.10E-02	-1.0912747	GOLT1B	golgi transport 1B	51026
37145_at	0.1212	2.11E-02	-1.3219387	GNLY	granulysin	10578
204658_at	0.1213	2.12E-02	-1.053302	TRA2A	transformer 2 alpha homolog	29896
201721_s_at	0.1214	2.12E-02	-1.113978	LAPTM5	lysosomal protein transmembrane 5	7805

205644_s_at	0.1214	2.12E-02	-1.2751273	SNRPG	small nuclear ribonucleoprotein polypeptide G	6637
214363_s_at	0.1215	2.12E-02	-1.0031973	SNHG4///MATR3	small nucleolar RNA host gene 4///matrin 3	724102///9782
212406_s_at	0.1215	2.12E-02	-1.061346	PCMTD2	protein-L-isoaspartate (D-aspartate) O-methyltransferase domain containing 2	55251
217828_at	0.1215	2.12E-02	-1.1111193	SLTM	SAFB like transcription modulator	79811
208617_s_at	0.1215	2.12E-02	-1.1642853	PTP4A2	protein tyrosine phosphatase type IVA, member 2	8073
217747_s_at	0.1215	2.12E-02	-1.395674	RPS9	ribosomal protein S9	6203
201581_at	0.1216	2.13E-02	-1.389212	TMX4	thioredoxin related transmembrane protein 4	56255

223836_at	0.1216	2.13E-02	-1.4716353	FGFBP2	fibroblast growth factor binding protein 2	83888
209027_s_at	0.1216	2.13E-02	-1.1175287	ABI1	abl interactor 1	10006
202506_at	0.1217	2.13E-02	-1.0524967	SSFA2	sperm specific antigen 2	6744
202158_s_at	0.1217	2.13E-02	-1.023806	CELF2	CUGBP, Elav-like family member 2	10659
202720_at	0.1217	2.14E-02	-1.0124167	TES	testin LIM domain protein	26136
214574_x_at	0.1217	2.14E-02	-1.1556967	LST1	leukocyte specific transcript 1	7940
222976_s_at	0.1218	2.14E-02	-1.0587853	TPM3	tropomyosin 3	7170
201011_at	0.1218	2.14E-02	-1.0604233	RPN1	ribophorin I	6184
212301_at	0.1219	2.15E-02	-1.0507347	RTF1	RTF1 homolog, Paf1/RNA polymerase II complex component	23168

225220_at	0.1219	2.15E-02	-1.1486993	SNHG8///SNORA24	small nucleolar RNA host gene 8///small nucleolar RNA, H/ACA box 24	100093630///677809
238034_at	0.1219	2.15E-02	-1.093942	CANX	calnexin	821
201597_at	0.1222	2.16E-02	-1.261106	COX7A2	cytochrome c oxidase subunit 7A2	1347
201008_s_at	0.1222	2.16E-02	-1.3658173	TXNIP	thioredoxin interacting protein	10628
224867_at	0.1222	2.16E-02	-1.3649087	MINOS1	mitochondrial inner membrane organizing system 1	440574
235556_at	0.1222	2.16E-02	-1.076398	CREBRF	CREB3 regulatory factor	153222
206380_s_at	0.1223	2.17E-02	-1.08494	CFP	complement factor properdin	5199
225663_at	0.1224	2.17E-02	-1.1370133	ACBD5	acyl-CoA binding domain containing 5	91452
202471_s_at	0.1224	2.17E-02	-1.012998	IDH3G	isocitrate dehydrogenase 3 (NAD(+)) gamma	3421

224964_s_at	0.1227	2.18E-02	-1.265488	GNG2	G protein subunit gamma 2	54331
218277_s_at	0.1227	2.19E-02	-1.1284	DHX40	DEAH-box helicase 40	79665
200079_s_at	0.1227	2.19E-02	-1.1204307	KARS	lysyl-tRNA synthetase	3735
217762_s_at	0.1228	2.19E-02	-1.0201047	RAB31	RAB31, member RAS oncogene family	11031
226430_at	0.1228	2.19E-02	-1.2537827	RELL1	RELT like 1	768211
224806_at	0.1228	2.19E-02	-1.0243867	TRIM25	tripartite motif containing 25	7706
209814_at	0.1228	2.19E-02	-1.1178673	ZNF330	zinc finger protein 330	27309
209028_s_at	0.1228	2.20E-02	-1.2726973	ABI1	abl interactor 1	10006
208746_x_at	0.1228	2.20E-02	-1.1964753	ATP5L	ATP synthase, H+ transporting, mitochondrial Fo complex subunit G	10632
200009_at	0.123	2.20E-02	-1.1963233	GDI2	GDP dissociation inhibitor 2	2665

209630_s_at	0.1231	2.22E-02	-1.1272147	FBXW2	F-box and WD repeat domain containing 2	26190
212993_at	0.1231	2.22E-02	-1.1696867	NACC2	NACC family member 2	138151
201177_s_at	0.1231	2.22E-02	-1.232522	UBA2	ubiquitin like modifier activating enzyme 2	10054
201133_s_at	0.1231	2.23E-02	-1.4486587	PJA2	praja ring finger ubiquitin ligase 2	9867
210606_x_at	0.1231	2.23E-02	-1.3592667	KLRD1	killer cell lectin like receptor D1	3824
202557_at	0.1231	2.23E-02	-1.0479187	HSPA13	heat shock protein family A (Hsp70) member 13	6782
203028_s_at	0.1233	2.23E-02	-1.2241533	CYBA	cytochrome b-245 alpha chain	1535

213251_at	0.1233	2.24E-02	-1.027726	SMARCA5	SWI/SNF related, matrix associated, actin dependent regulator of chromatin, subfamily a, member 5	8467
226414_s_at	0.1235	2.25E-02	-1.0552033	ANAPC11	anaphase promoting complex subunit 11	51529
215193_x_at	0.1235	2.25E-02	-1.6830913	LOC101060835///LOC100996809///HLA-DRB4///HLA-DRB3///HLA-DRB1///HLA-DQB1	HLA class II histocompatibility antigen, DQ beta 1 chain-like///HLA class II histocompatibility antigen, DRB1-10 beta chain-like///major histocompatibility complex, class II, DR beta 4///major histocompatibility complex, class II, DR beta 3///major	101060835///100996809///3126///3125///3123///3119

212646_at	0.1235	2.25E-02	-1.05469	RFTN1	raftlin, lipid raft linker 1	23180
218396_at	0.1235	2.25E-02	-1.1103607	VPS13C	vacuolar protein sorting 13 homolog C	54832
202499_s_at	0.1237	2.25E-02	-1.041182	SLC2A3	solute carrier family 2 member 3	6515
223210_at	0.1237	2.26E-02	-1.3433133	CHURC1	churchill domain containing 1	91612
215096_s_at	0.1238	2.26E-02	-1.003834	ESD	esterase D	2098
209422_at	0.1239	2.26E-02	-1.011508	PHF20	PHD finger protein 20	51230
215884_s_at	0.1239	2.27E-02	-1.1629607	UBQLN2	ubiquilin 2	29978
200968_s_at	0.124	2.27E-02	-1.2866467	PPIB	peptidylprolyl isomerase B	5479
211025_x_at	0.124	2.27E-02	-1.3600247	COX5B	cytochrome c oxidase subunit 5B	1329
212640_at	0.1241	2.28E-02	-1.046838	HACD2	3-hydroxyacyl-CoA dehydratase 2	201562
226538_at	0.1242	2.28E-02	-1.007248	MAN2A1	mannosidase alpha class 2A member 1	4124
219598_s_at	0.1242	2.28E-02	-1.08579	RWDD1	RWD domain containing 1	51389

207132_x_at	0.1242	2.28E-02	-1.21176	PFDN5	prefoldin subunit 5	5204
225844_at	0.1243	2.29E-02	-1.2424873	POLE4	DNA polymerase epsilon 4, accessory subunit	56655
201410_at	0.1244	2.29E-02	-1.0800933	PLEKHB2	pleckstrin homology domain containing B2	55041
205786_s_at	0.1244	2.29E-02	-1.4813813	ITGAM	integrin subunit alpha M	3684
222000_at	0.1246	2.30E-02	-1.1128827	C1orf174	chromosome 1 open reading frame 174	339448
217915_s_at	0.1247	2.32E-02	-1.190308	RSL24D1	ribosomal L24 domain containing 1	51187
201855_s_at	0.1248	2.32E-02	-1.07366	ATMIN	ATM interactor	23300
213939_s_at	0.1249	2.32E-02	-1.0869953	RUFY3	RUN and FYVE domain containing 3	22902

201973_s_at	0.125	2.33E-02	-1.1196773	CCZ1B///CCZ1	CCZ1 homolog B, vacuolar protein trafficking and biogenesis associated///CCZ1 homolog, vacuolar protein trafficking and biogenesis associated	221960///51622
216088_s_at	0.125	2.33E-02	-1.3208213	PSMA7	proteasome subunit alpha 7	5688
225564_at	0.125	2.34E-02	-1.0706027	SPATA13	spermatogenesis associated 13	221178
201022_s_at	0.1252	2.35E-02	-1.0624193	DSTN	destrin, actin depolymerizing factor	11034
201352_at	0.1252	2.35E-02	-1.070132	YME1L1	YME1 like 1 ATPase	10730
224664_at	0.1253	2.35E-02	-1.1454627	ANAPC16	anaphase promoting complex subunit 16	119504

201411_s_at	0.1253	2.35E-02	-1.1432273	PLEKHB2	pleckstrin homology domain containing B2	55041
202704_at	0.1253	2.35E-02	-1.212268	TOB1	transducer of ERBB2, 1	10140
225282_at	0.1253	2.36E-02	-1.0399987	SMAP2	small ArfGAP2	64744
209023_s_at	0.1254	2.36E-02	-1.2589253	STAG2	stromal antigen 2	10735
217758_s_at	0.1254	2.36E-02	-1.04846	TM9SF3	transmembrane 9 superfamily member 3	56889
218486_at	0.1255	2.36E-02	-1.128588	KLF11	Kruppel like factor 11	8462
201533_at	0.1255	2.36E-02	-1.1236753	CTNNB1	catenin beta 1	1499
222396_at	0.1255	2.37E-02	-1.119482	HN1	hematological and neurological expressed 1	51155
224597_at	0.1256	2.37E-02	-1.23485	NORAD	non-coding RNA activated by DNA damage	647979
200967_at	0.1257	2.37E-02	-1.1737227	PPIB	peptidylprolyl isomerase B	5479
200056_s_at	0.1257	2.38E-02	-1.2839567	C1D	C1D nuclear receptor corepressor	10438

217985_s_at	0.1257	2.38E-02	-1.0199847	BAZ1A	bromodomain adjacent to zinc finger domain 1A	11177
217836_s_at	0.1258	2.38E-02	-1.108088	YY1AP1	YY1 associated protein 1	55249
201699_at	0.1259	2.39E-02	-1.124078	PSMC6	proteasome 26S subunit, ATPase 6	5706
212263_at	0.1259	2.39E-02	-1.2244533	QKI	QKI, KH domain containing RNA binding	9444
209669_s_at	0.1259	2.39E-02	-1.0202447	SERBP1	SERPINE1 mRNA binding protein 1	26135
201812_s_at	0.126	2.39E-02	-1.012046	C4orf46///TOMM7	chromosome 4 open reading frame 46///translocase of outer mitochondrial membrane 7	201725///54543
203186_s_at	0.126	2.39E-02	-1.0332947	S100A4	S100 calcium binding protein A4	6275
223059_s_at	0.1261	2.40E-02	-1.0532067	FAM107B	family with sequence similarity 107 member B	83641

205668_at	0.1261	2.41E-02	-1.0941087	LY75	lymphocyte antigen 75	4065
226091_s_at	0.1261	2.41E-02	-1.106824	MRFAP1	Morf4 family associated protein 1	93621
210501_x_at	0.1261	2.41E-02	-1.081136	EIF3K	eukaryotic translation initiation factor 3 subunit K	27335
208729_x_at	0.1261	2.41E-02	-1.1098833	HLA-B	major histocompatibility complex, class I, B	3106
208909_at	0.1262	2.41E-02	-1.1603307	UQCRFS1	ubiquinol-cytochrome c reductase, Rieske iron-sulfur polypeptide 1	7386
228690_s_at	0.1262	2.41E-02	-1.009892	NDUFA11	NADH:ubiquinone oxidoreductase subunit A11	126328
202467_s_at	0.1263	2.42E-02	-1.2791473	COPS2	COP9 signalosome subunit 2	9318

209846_s_at	0.1263	2.42E-02	-1.37438	BTN3A2	butyrophilin subfamily 3 member A2	11118
203177_x_at	0.1263	2.42E-02	-1.1515407	TFAM	transcription factor A, mitochondrial	7019
203148_s_at	0.1265	2.43E-02	-1.0774907	TRIM14	tripartite motif containing 14	9830
208801_at	0.1265	2.43E-02	-1.1435187	SRP72	signal recognition particle 72	6731
200078_s_at	0.1265	2.43E-02	-1.095712	ATP6V0B	ATPase H+ transporting V0 subunit b	533
208669_s_at	0.1265	2.43E-02	-1.2725673	EID1	EP300 interacting inhibitor of differentiation 1	23741
224719_s_at	0.1265	2.43E-02	-1.309054	C12orf57	chromosome 12 open reading frame 57	113246
226694_at	0.1265	2.44E-02	-1.1223793	PALM2-AKAP2///AKAP2	PALM2-AKAP2 readthrough///A-kinase anchoring protein 2	445815///11217

214214_s_at	0.1266	2.44E-02	-1.3694413	C1QBP	complement C1q binding protein	708
1552628_a_at	0.1267	2.44E-02	-1.1564547	HERPUD2	HERPUD family member 2	64224
221771_s_at	0.1268	2.44E-02	-1.1003487	MPHOSPH8	M-phase phosphoprotein 8	54737
223178_s_at	0.1268	2.44E-02	-1.02465	NT5DC1	5'-nucleotidase domain containing 1	221294
200823_x_at	0.1269	2.45E-02	-1.1385633	RPL29	ribosomal protein L29	6159
200992_at	0.1269	2.45E-02	-1.0409067	IPO7	importin 7	10527
212043_at	0.127	2.45E-02	-1.0026787	TGOLN2	trans-golgi network protein 2	10618
209033_s_at	0.1271	2.46E-02	-1.012388	DYRK1A	dual specificity tyrosine phosphorylation regulated kinase 1A	1859
31845_at	0.1272	2.46E-02	-1.0271913	ELF4	E74 like ETS transcription factor 4	2000
211762_s_at	0.1272	2.47E-02	-1.075998	KPNA2	karyopherin subunit alpha 2	3838
221012_s_at	0.1272	2.47E-02	-1.1201353	TRIM8	tripartite motif containing 8	81603

203137_at	0.1273	2.47E-02	-1.1024987	WTAP	Wilms tumor 1 associated protein	9589
225312_at	0.1274	2.47E-02	-1.1558713	COMMD6	COMM domain containing 6	170622
217801_at	0.1274	2.48E-02	-1.0057967	ATP5E	ATP synthase, H+ transporting, mitochondrial F1 complex, epsilon subunit	514
217478_s_at	0.1274	2.48E-02	-1.4417893	HLA-DMA	major histocompatibility complex, class II, DM alpha	3108
213735_s_at	0.1275	2.49E-02	-1.0171273	COX5B	cytochrome c oxidase subunit 5B	1329
202318_s_at	0.1276	2.49E-02	-1.0151913	SENP6	SUMO1/sentrin specific peptidase 6	26054
223640_at	0.1276	2.50E-02	-1.23292	HCST	hematopoietic cell signal transducer	10870
218258_at	0.1278	2.51E-02	-1.2535433	POLR1D	RNA polymerase I subunit D	51082

231735_s_at	0.128	2.51E-02	1.0309027	MALAT1	metastasis associated lung adenocarcinoma transcript 1 (non-protein coding)	378938
203227_s_at	0.128	2.51E-02	-1.068578	TSPAN31	tetraspanin 31	6302
203396_at	0.1282	2.52E-02	-1.165808	PSMA4	proteasome subunit alpha 4	5685
218031_s_at	0.1282	2.52E-02	-1.248052	FOXN3	forkhead box N3	1112
208689_s_at	0.1284	2.53E-02	-1.0591207	RPN2	ribophorin II	6185
212973_at	0.1284	2.53E-02	-1.0046787	RPIA	ribose 5-phosphate isomerase A	22934
208948_s_at	0.1284	2.53E-02	-1.047306	STAU1	staufen double-stranded RNA binding protein 1	6780
212878_s_at	0.1284	2.53E-02	-1.0683633	KLC1	kinesin light chain 1	3831
238768_at	0.1284	2.54E-02	-1.085484	C2orf68	chromosome 2 open reading frame 68	388969
202077_at	0.1287	2.55E-02	-1.0600547	NDUFAB1	NADH:ubiquinone oxidoreductase subunit AB1	4706

217739_s_at	0.1287	2.55E-02	-1.708762	NAMPT	nicotinamide phosphoribosyltransferase	10135
202546_at	0.1288	2.56E-02	-1.3791513	VAMP8	vesicle associated membrane protein 8	8673
231948_s_at	0.1288	2.56E-02	-1.1693487	UBE2F	ubiquitin conjugating enzyme E2 F (putative)	140739
213720_s_at	0.1288	2.56E-02	-1.003288	SMARCA4	SWI/SNF related, matrix associated, actin dependent regulator of chromatin, subfamily a, member 4	6597
207795_s_at	0.1289	2.56E-02	-1.132576	KLRD1	killer cell lectin like receptor D1	3824
202888_s_at	0.1289	2.56E-02	-1.0411927	ANPEP	alanyl aminopeptidase, membrane	290

227639_at	0.1289	2.57E-02	-1.2736973	PIGK	phosphatidylinositol glycan anchor biosynthesis class K	10026
226353_at	0.1289	2.57E-02	-1.0223527	SPPL2A	signal peptide peptidase like 2A	84888
202657_s_at	0.1289	2.57E-02	-1.2907707	SERTAD2	SERTA domain containing 2	9792
219329_s_at	0.1289	2.57E-02	-1.134154	ATRAID	all-trans retinoic acid induced differentiation factor	51374
200873_s_at	0.129	2.57E-02	-1.2162313	CCT8	chaperonin containing TCP1 subunit 8	10694
209154_at	0.129	2.57E-02	-1.5101773	P2RX5-TAX1BP3///TAX1BP3	P2RX5-TAX1BP3 readthrough (NMD candidate)///Tax1 binding protein 3	100533970///30851
223310_x_at	0.129	2.58E-02	-1.2114427	PNPLA8	patatin like phospholipase domain containing 8	50640
203075_at	0.1291	2.58E-02	-1.2464393	SMAD2	SMAD family member 2	4087

208722_s_at	0.1291	2.58E-02	-1.026502	ANAPC5	anaphase promoting complex subunit 5	51433
239143_x_at	0.1292	2.59E-02	-1.023532	RNF138	ring finger protein 138	51444
217728_at	0.1292	2.59E-02	-1.2215733	S100A6	S100 calcium binding protein A6	6277
211942_x_at	0.1293	2.60E-02	-1.1101647	RPL13AP5///RPL13AP6///SNORD32A///SNORD33///SNORD34///SNORD35A///RPL13A	ribosomal protein L13a pseudogene 5///ribosomal protein L13a pseudogene 6///small nucleolar RNA, C/D box 32A///small nucleolar RNA, C/D box 33///small nucleolar RNA, C/D box 34///small nucleolar RNA, C/D box	728658///644511///26819///26818///26817///26816///23521
205335_s_at	0.1293	2.60E-02	-1.0159393	SRP19	signal recognition particle 19	6728
203647_s_at	0.1294	2.61E-02	-1.0590693	FDX1	ferredoxin 1	2230

200004_at	0.1294	2.61E-02	-1.0940313	EIF4G2	eukaryotic translation initiation factor 4 gamma 2	1982
204559_s_at	0.1294	2.61E-02	-1.0619307	LSM7	LSM7 homolog, U6 small nuclear RNA and mRNA degradation associated	51690
200650_s_at	0.1295	2.62E-02	-1.1206747	LDHA	lactate dehydrogenase A	3939
212998_x_at	0.1295	2.62E-02	-1.0549213	LOC101060835///HLA-DQB1	HLA class II histocompatibility antigen, DQ beta 1 chain-like///major histocompatibility complex, class II, DQ beta 1	101060835///3119
221749_at	0.1295	2.62E-02	-1.2303547	YTHDF3	YTH N6-methyladenosine RNA binding protein 3	253943
200999_s_at	0.1295	2.62E-02	-1.0696993	CKAP4	cytoskeleton associated protein 4	10970

228853_at	0.1295	2.62E-02	-1.0139613	STYX	serine/threonine/tyrosine interacting protein	6815
202591_s_at	0.1298	2.63E-02	-1.5452047	SSBP1	single stranded DNA binding protein 1	6742
1556209_at	0.1299	2.64E-02	-1.14528	CLEC2B	C-type lectin domain family 2 member B	9976
227447_at	0.1302	2.65E-02	-1.050046	SKIV2L2	Ski2 like RNA helicase 2	23517
224446_at	0.1302	2.65E-02	-1.026722	LLPH	LLP homolog, long-term synaptic facilitation	84298
225849_s_at	0.1303	2.66E-02	-1.360718	SFT2D1	SFT2 domain containing 1	113402
211581_x_at	0.1304	2.66E-02	-1.1367693	LST1	leukocyte specific transcript 1	7940
200086_s_at	0.1304	2.66E-02	-1.0418247	COX4I1	cytochrome c oxidase subunit 4I1	1327

209654_at	0.1305	2.67E-02	-1.0723313	ICE1	interactor of little elongation complex ELL subunit 1	23379
211275_s_at	0.1305	2.67E-02	-1.192702	GYG1	glycogenin 1	2992
203663_s_at	0.1306	2.67E-02	-1.1829593	COX5A	cytochrome c oxidase subunit 5A	9377
214211_at	0.1306	2.67E-02	-1.048072	FTH1	ferritin heavy chain 1	2495
209515_s_at	0.1306	2.67E-02	-1.0234847	RAB27A	RAB27A, member RAS oncogene family	5873
200631_s_at	0.1306	2.68E-02	-1.087156	SETSIP///SET	SET-like protein///SET nuclear proto-oncogene	646817///6418
224747_at	0.1306	2.68E-02	-1.2228093	UBE2Q2	ubiquitin conjugating enzyme E2 Q2	92912
201754_at	0.1307	2.68E-02	-1.157348	COX6C	cytochrome c oxidase subunit 6C	1345
200869_at	0.1308	2.69E-02	-1.199022	SNORA68///RPL18A	small nucleolar RNA, H/ACA box 68///ribosomal protein L18a	26780///6142

1555938_x_at	0.1308	2.69E-02	-1.348502	VIM	vimentin	7431
202088_at	0.131	2.70E-02	-1.0987947	SLC39A6	solute carrier family 39 member 6	25800
201443_s_at	0.131	2.70E-02	-1.2610433	ATP6AP2	ATPase H+ transporting accessory protein 2	10159
213988_s_at	0.131	2.70E-02	-1.2372327	SAT1	spermidine/spermine N1-acetyltransferase 1	6303
212410_at	0.1314	2.71E-02	-1.1607833	MICU2	mitochondrial calcium uptake 2	221154
227087_at	0.1316	2.73E-02	-1.0882833	INPP4A	inositol polyphosphate-4-phosphatase type I A	3631
238706_at	0.1316	2.73E-02	-1.021754	PAPD4	poly(A) RNA polymerase D4, non-canonical	167153

210396_s_at	0.1317	2.74E-02	-1.0031	SMG1P5///BOLA2///SMG1P2	SMG1P5, nonsense mediated mRNA decay associated PI3K related kinase pseudogene 5///bolA family member 2///SMG1P2, nonsense mediated mRNA decay associated PI3K related kinase pseudogene 2	595101///552900///440354
222992_s_at	0.1318	2.75E-02	-1.33543	NDUFB9	NADH:ubiquinone oxidoreductase subunit B9	4715
205495_s_at	0.1318	2.75E-02	-1.4023327	GNLY	granulysin	10578
201541_s_at	0.1318	2.75E-02	-1.0779153	ZNHIT1	zinc finger HIT-type containing 1	10467
212511_at	0.1319	2.75E-02	-1.3365147	PICALM	phosphatidylinositol binding clathrin assembly protein	8301

212131_at	0.1319	2.76E-02	-1.24631	LSM14A	LSM14A, mRNA processing body assembly factor	26065
209674_at	0.1319	2.76E-02	-1.184342	CRY1	cryptochrome circadian clock 1	1407
200891_s_at	0.132	2.76E-02	-1.3061733	SSR1	signal sequence receptor subunit 1	6745
224826_at	0.132	2.76E-02	-1.0557033	GPCPD1	glycerophosphocholine phosphodiesterase 1	56261
225845_at	0.132	2.76E-02	-1.27613	ZBTB44	zinc finger and BTB domain containing 44	29068
200640_at	0.1322	2.77E-02	-1.1100713	YWHAZ	tyrosine 3-monooxygenase/tryptophan 5-monooxygenase activation protein zeta	7534
222572_at	0.1322	2.77E-02	-1.0110267	PDP1	pyruvate dehyrogenase phosphatase catalytic subunit 1	54704

212040_at	0.1322	2.78E-02	-1.4092473	TGOLN2	trans-golgi network protein 2	10618
225334_at	0.1322	2.78E-02	-1.0128067	BORCS7	BLOC-1 related complex subunit 7	119032
211368_s_at	0.1322	2.78E-02	-1.57353	CASP1	caspase 1	834
202428_x_at	0.1322	2.78E-02	-1.114962	DBI	diazepam binding inhibitor, acyl-CoA binding protein	1622
213366_x_at	0.1322	2.78E-02	-1.0294807	ATP5C1	ATP synthase, H+ transporting, mitochondrial F1 complex, gamma polypeptide 1	509
202698_x_at	0.1322	2.78E-02	-1.174188	COX4I1	cytochrome c oxidase subunit 4I1	1327
208864_s_at	0.1323	2.79E-02	-1.502384	TXN	thioredoxin	7295
235615_at	0.1324	2.79E-02	-1.081254	PGGT1B	protein geranylgeranyltransferase type I subunit beta	5229
226835_s_at	0.1325	2.79E-02	-1.0180693	ZFAS1	ZNFX1 antisense RNA 1	441951

201290_at	0.1325	2.79E-02	-1.0204447	SEC11A	SEC11 homolog A, signal peptidase complex subunit	23478
212334_at	0.1325	2.80E-02	-1.3606653	GNS	glucosamine (N-acetyl)-6-sulfatase	2799
222390_at	0.1325	2.80E-02	-1.0092113	WAC	WW domain containing adaptor with coiled-coil	51322
212671_s_at	0.1325	2.80E-02	-1.5687573	LOC100509457///HLA-DQA2///HLA-DQA1	HLA class II histocompatibility antigen, DQ alpha 1 chain-like///major histocompatibility complex, class II, DQ alpha 2///major histocompatibility complex, class II, DQ alpha 1	100509457///3118///3117
222555_s_at	0.1326	2.80E-02	-1.00818	MRPL44	mitochondrial ribosomal protein L44	65080

201816_s_at	0.1326	2.80E-02	-1.1984587	GBAS	glioblastoma amplified sequence	2631
222391_at	0.1327	2.81E-02	-1.1339213	TMEM30A	transmembrane protein 30A	55754
213915_at	0.1327	2.81E-02	-1.282978	NKG7	natural killer cell granule protein 7	4818
201456_s_at	0.1327	2.81E-02	-1.021654	BUB3	BUB3, mitotic checkpoint protein	9184
226413_at	0.1327	2.82E-02	-1.2191047	LINC00938	long intergenic non-protein coding RNA 938	400027
225783_at	0.1328	2.82E-02	-1.0257047	UBE2F	ubiquitin conjugating enzyme E2 F (putative)	140739
201123_s_at	0.1329	2.82E-02	-1.4915653	EIF5A	eukaryotic translation initiation factor 5A	1984
208981_at	0.1329	2.82E-02	-1.10458	PECAM1	platelet and endothelial cell adhesion molecule 1	5175

224779_s_at	0.133	2.83E-02	-1.3045467	FAM96A	family with sequence similarity 96 member A	84191
225956_at	0.1331	2.84E-02	-1.0565833	CREBRF	CREB3 regulatory factor	153222
225912_at	0.1331	2.84E-02	-1.0682907	TP53INP1	tumor protein p53 inducible nuclear protein 1	94241
210275_s_at	0.1332	2.84E-02	-1.2574453	ZFAND5	zinc finger AN1-type containing 5	7763
211953_s_at	0.1337	2.87E-02	-1.0287267	IPO5	importin 5	3843
218106_s_at	0.1337	2.87E-02	-1.0462007	MRPS10	mitochondrial ribosomal protein S10	55173
201901_s_at	0.1337	2.87E-02	-1.0005553	YY1	YY1 transcription factor	7528
229584_at	0.1337	2.87E-02	-1.2630793	LRRK2	leucine rich repeat kinase 2	120892
218247_s_at	0.1338	2.87E-02	-1.0626447	MEX3C	mex-3 RNA binding family member C	51320

201210_at	0.1338	2.87E-02	-1.09981	DDX3X	DEAD-box helicase 3, X-linked	1654
200839_s_at	0.1338	2.87E-02	-1.0381947	CTSB	cathepsin B	1508
202651_at	0.1339	2.88E-02	-1.4439033	LPGAT1	lysophosphatidylglycerol acyltransferase 1	9926
228315_at	0.1339	2.88E-02	-1.0516947	ZMAT3	zinc finger matrin-type 3	64393
202114_at	0.134	2.89E-02	-1.0124327	SNX2	sorting nexin 2	6643
243296_at	0.1341	2.89E-02	-1.4464787	NAMPT	nicotinamide phosphoribosyltransferase	10135
224842_at	0.1341	2.89E-02	-1.13457	LOC101060386///BOLA2///SMG1	putative uncharacterized SMG1-like protein///bolA family member 2///SMG1, nonsense mediated mRNA decay associated PI3K related kinase	101060386///552900///23049
205992_s_at	0.1341	2.89E-02	-1.0301193	IL15	interleukin 15	3600
214288_s_at	0.1344	2.91E-02	-1.0187033	PSMB1	proteasome subunit beta 1	5689

1557905_s_at	0.1345	2.91E-02	-1.0124233	CD44	CD44 molecule (Indian blood group)	960
201588_at	0.1345	2.92E-02	-1.2294533	TXNL1	thioredoxin like 1	9352
214084_x_at	0.1347	2.93E-02	-1.0100327	NCF1	neutrophil cytosolic factor 1	653361
201038_s_at	0.1347	2.93E-02	-1.295958	ANP32A	acidic nuclear phosphoprotein 32 family member A	8125
203582_s_at	0.1348	2.93E-02	-1.1020387	SPHAR///RAB4A	S-phase response (cyclin related)///RAB4A, member RAS oncogene family	10638///5867
200970_s_at	0.1348	2.93E-02	-1.043876	SERP1	stress-associated endoplasmic reticulum protein 1	27230
212205_at	0.1348	2.93E-02	-1.1888653	H2AFV	H2A histone family member V	94239
211058_x_at	0.1349	2.94E-02	-1.022578	TUBA1B	tubulin alpha 1b	10376
200048_s_at	0.135	2.95E-02	-1.0739753	JTB	jumping translocation breakpoint	10899

212195_at	0.135	2.95E-02	-1.2665193	IL6ST	interleukin 6 signal transducer	3572
211939_x_at	0.135	2.95E-02	-1.0968747	BTF3	basic transcription factor 3	689
202386_s_at	0.135	2.95E-02	-1.1155267	KIAA0430	KIAA0430	9665
200706_s_at	0.135	2.95E-02	-1.0185387	LITAF	lipopolysaccharide induced TNF factor	9516
211911_x_at	0.1354	2.97E-02	-1.0508293	HLA-B	major histocompatibility complex, class I, B	3106
200738_s_at	0.1354	2.97E-02	-1.311176	PGK1	phosphoglycerate kinase 1	5230
208892_s_at	0.1354	2.97E-02	-1.71922	DUSP6	dual specificity phosphatase 6	1848
209075_s_at	0.1354	2.97E-02	-1.0087613	ISCU	iron-sulfur cluster assembly enzyme	23479
200669_s_at	0.1354	2.97E-02	-1.033918	UBE2D3	ubiquitin conjugating enzyme E2 D3	7323

226934_at	0.1355	2.98E-02	-1.1584673	CPSF6	cleavage and polyadenylation specific factor 6	11052
224569_s_at	0.1356	2.98E-02	-1.0953347	IRF2BP2	interferon regulatory factor 2 binding protein 2	359948
210031_at	0.1356	2.99E-02	-1.026316	CD247	CD247 molecule	919
225265_at	0.1356	2.99E-02	-1.0854567	RBMS1	RNA binding motif single stranded interacting protein 1	5937
217491_x_at	0.1356	2.99E-02	-1.010302	COX7C	cytochrome c oxidase subunit 7C	1350
228812_at	0.1357	2.99E-02	-1.2141927	REL	REL proto-oncogene, NF-kB subunit	5966
202325_s_at	0.1357	2.99E-02	-1.2421193	ATP5J	ATP synthase, H+ transporting, mitochondrial Fo complex subunit F6	522

200014_s_at	0.1358	3.00E-02	-1.077028	HNRNPC	heterogeneous nuclear ribonucleoprotein C (C1/C2)	3183
243916_x_at	0.1359	3.00E-02	-1.2434707	UBLCP1	ubiquitin like domain containing CTD phosphatase 1	134510
206245_s_at	0.1359	3.00E-02	-1.0693553	IVNS1ABP	influenza virus NS1A binding protein	10625
223000_s_at	0.136	3.01E-02	-1.00076	F11R	F11 receptor	50848
221478_at	0.136	3.01E-02	-1.2819967	BNIP3L	BCL2 interacting protein 3 like	665
217851_s_at	0.1361	3.02E-02	-1.11869	PRELID3B	PRELI domain containing 3B	51012
217812_at	0.1361	3.02E-02	-1.181462	YTHDF2	YTH N6-methyladenosine RNA binding protein 2	51441
200881_s_at	0.1361	3.02E-02	-1.0242007	DNAJA1	DnaJ heat shock protein family (Hsp40) member A1	3301

201399_s_at	0.1362	3.02E-02	-1.142324	TRAM1	translocation associated membrane protein 1	23471
211666_x_at	0.1363	3.03E-02	-1.060362	SNORD83B///SNORD139///RPL3	small nucleolar RNA, C/D box 83B///small nucleolar RNA, C/D box 139///ribosomal protein L3	116938///116936///6122
225658_at	0.1363	3.03E-02	-1.073356	SPOPL	speckle type BTB/POZ protein like	339745
200782_at	0.1363	3.03E-02	-1.2313793	ANXA5	annexin A5	308
224972_at	0.1364	3.05E-02	-1.1171473	ROMO1	reactive oxygen species modulator 1	140823
218334_at	0.1364	3.05E-02	-1.18705	THOC7	THO complex 7	80145
213453_x_at	0.1364	3.05E-02	-1.1125587	GAPDH	glyceraldehyde-3-phosphate dehydrogenase	2597

211997_x_at	0.1364	3.05E-02	-1.131198	MIR4738///H3F3B///H3F3A	microRNA 4738///H3 histone, family 3B///H3 histone, family 3A	100616282///3021///3020
225123_at	0.1365	3.05E-02	-1.0514953	SESN3	sestrin 3	143686
221547_at	0.1365	3.05E-02	-1.0652193	PRPF18	pre-mRNA processing factor 18	8559
200971_s_at	0.1365	3.06E-02	-1.0460573	SERP1	stress-associated endoplasmic reticulum protein 1	27230
201258_at	0.1365	3.06E-02	-1.1410313	RPS16	ribosomal protein S16	6217
200826_at	0.1365	3.06E-02	-1.006994	SNRPD2	small nuclear ribonucleoprotein D2 polypeptide	6633
1562836_at	0.1366	3.06E-02	1.1219193	DDX6	DEAD-box helicase 6	1656
200910_at	0.1367	3.07E-02	-1.0808887	LOC101927137///CCT3	uncharacterized LOC101927137///chaperonin containing TCP1 subunit 3	101927137///7203

217982_s_at	0.1367	3.07E-02	-1.2368147	MORF4L1	mortality factor 4 like 1	10933
204220_at	0.1368	3.07E-02	-1.068022	GMFG	glia maturation factor gamma	9535
203721_s_at	0.137	3.08E-02	-1.191928	UTP18	UTP18, small subunit processome component	51096
202113_s_at	0.137	3.08E-02	-1.2471627	SNX2	sorting nexin 2	6643
202232_s_at	0.137	3.09E-02	-1.331628	EIF3M	eukaryotic translation initiation factor 3 subunit M	10480
202131_s_at	0.1371	3.09E-02	-1.2472273	RIOK3	RIO kinase 3	8780
212221_x_at	0.1371	3.09E-02	-1.0421307	IDS	iduronate 2-sulfatase	3423
200819_s_at	0.1375	3.11E-02	-1.0083487	RPS15	ribosomal protein S15	6209
200740_s_at	0.1377	3.12E-02	-1.1778627	SUMO3	small ubiquitin-like modifier 3	6612

201761_at	0.1377	3.13E-02	-1.1516867	MTHFD2	methylenetetrahydrofolate dehydrogenase (NADP+ dependent) 2, methenyltetrahydrofolate cyclohydrolase	10797
210250_x_at	0.1378	3.13E-02	-1.0013787	ADSL	adenylosuccinate lyase	158
201921_at	0.1379	3.14E-02	-1.1292973	GNG10	G protein subunit gamma 10	2790
200700_s_at	0.1381	3.15E-02	-1.09437	KDELR2	KDEL endoplasmic reticulum protein retention receptor 2	11014
40189_at	0.1382	3.16E-02	-1.151368	SETSIP///SET	SET-like protein///SET nuclear proto-oncogene	646817///6418
202984_s_at	0.1382	3.16E-02	-1.0357633	BAG5	BCL2 associated athanogene 5	9529
209796_s_at	0.1382	3.16E-02	-1.0410333	CNPY2	canopy FGF signaling regulator 2	10330

212773_s_at	0.1383	3.17E-02	-1.1022507	TOMM20	translocase of outer mitochondrial membrane 20	9804
200667_at	0.1384	3.18E-02	-1.0125193	UBE2D3	ubiquitin conjugating enzyme E2 D3	7323
200037_s_at	0.1384	3.18E-02	-1.0295093	CBX3	chromobox 3	11335
226319_s_at	0.1385	3.18E-02	-1.3937267	ALYREF	Aly/REF export factor	10189
208993_s_at	0.1385	3.19E-02	-1.0190033	PPIG	peptidylprolyl isomerase G	9360
201327_s_at	0.1387	3.20E-02	-1.0400373	CCT6A	chaperonin containing TCP1 subunit 6A	908
201877_s_at	0.1387	3.21E-02	-1.0450913	PPP2R5C	protein phosphatase 2 regulatory subunit B'gamma	5527
202842_s_at	0.1387	3.21E-02	-1.0746147	DNAJB9	DnaJ heat shock protein family (Hsp40) member B9	4189
212560_at	0.1388	3.21E-02	-1.0199747	SORL1	sortilin related receptor 1	6653

202149_at	0.1389	3.22E-02	-1.039772	NEDD9	neural precursor cell expressed, developmentally down-regulated 9	4739
223106_at	0.1389	3.22E-02	-1.2134427	TMEM14C	transmembrane protein 14C	51522
202244_at	0.1391	3.24E-02	-1.0364993	PSMB4	proteasome subunit beta 4	5692
224983_at	0.1392	3.24E-02	-1.0320047	SCARB2	scavenger receptor class B member 2	950
218223_s_at	0.1392	3.24E-02	-1.0352853	PLEKHO1	pleckstrin homology domain containing O1	51177
204479_at	0.1392	3.25E-02	-1.1436173	OSTF1	osteoclast stimulating factor 1	26578
221931_s_at	0.1392	3.25E-02	-1.0320293	SEH1L	SEH1 like nucleoporin	81929
224731_at	0.1393	3.25E-02	-1.0225407	HMGB1	high mobility group box 1	3146
226392_at	0.1393	3.25E-02	-1.037322	RASA2	RAS p21 protein activator 2	5922

201400_at	0.1394	3.26E-02	-1.2002233	PSMB3	proteasome subunit beta 3	5691
212245_at	0.1394	3.26E-02	-1.0937567	MCFD2	multiple coagulation factor deficiency 2	90411
200010_at	0.1394	3.26E-02	-1.001946	RPL11	ribosomal protein L11	6135
209389_x_at	0.1394	3.26E-02	-1.1382487	DBI	diazepam binding inhibitor, acyl-CoA binding protein	1622
218577_at	0.1395	3.27E-02	-1.0218893	LRRC40	leucine rich repeat containing 40	55631
200750_s_at	0.1395	3.27E-02	-1.2835673	RAN	RAN, member RAS oncogene family	5901
200726_at	0.1396	3.27E-02	-1.0857127	PPP1CC	protein phosphatase 1 catalytic subunit gamma	5501
209046_s_at	0.1396	3.28E-02	-1.1186813	GABARAPL2	GABA type A receptor associated protein like 2	11345

226905_at	0.1396	3.28E-02	-1.157796	FAM101B	family with sequence similarity 101 member B	359845
208700_s_at	0.1396	3.28E-02	-1.208766	TKT	transketolase	7086
222435_s_at	0.1396	3.28E-02	-1.0840247	UBE2J1	ubiquitin conjugating enzyme E2 J1	51465
209106_at	0.1396	3.28E-02	-1.034604	NCOA1	nuclear receptor coactivator 1	8648
208946_s_at	0.14	3.30E-02	-1.0093487	BECN1	beclin 1	8678
201088_at	0.14	3.30E-02	-1.1241233	KPNA2	karyopherin subunit alpha 2	3838
204122_at	0.1401	3.31E-02	-1.4955473	TYROBP	TYRO protein tyrosine kinase binding protein	7305
217763_s_at	0.1401	3.31E-02	-1.1590313	RAB31	RAB31, member RAS oncogene family	11031
201312_s_at	0.1402	3.31E-02	-1.1081727	SH3BGRL	SH3 domain binding glutamate rich protein like	6451
209388_at	0.1402	3.31E-02	-1.186932	PAPOLA	poly(A) polymerase alpha	10914

229145_at	0.1402	3.31E-02	-1.1528133	ANAPC16	anaphase promoting complex subunit 16	119504
214429_at	0.1403	3.32E-02	-1.1265147	MTMR6	myotubularin related protein 6	9107
220864_s_at	0.1404	3.32E-02	-1.0304947	NDUFA13	NADH:ubiquinone oxidoreductase subunit A13	51079
214500_at	0.1404	3.32E-02	-1.294848	H2AFY	H2A histone family member Y	9555
224935_at	0.1404	3.32E-02	-1.138824	EIF2S3	eukaryotic translation initiation factor 2 subunit gamma	1968
233759_s_at	0.1405	3.33E-02	-1.0382673	PPP4R3B	protein phosphatase 4 regulatory subunit 3B	57223
228999_at	0.1406	3.34E-02	-1.1153913	CHD2	chromodomain helicase DNA binding protein 2	1106

1555691_a_at	0.1408	3.35E-02	-1.1143653	KLRC4-KLRK1///KLRK1	KLRC4-KLRK1 readthrough///killer cell lectin like receptor K1	100528032///22914
224691_at	0.1408	3.35E-02	-1.1447167	UHMK1	U2AF homology motif (UHM) kinase 1	127933
221509_at	0.1409	3.36E-02	-1.099616	DENR	density regulated re-initiation and release factor	8562
202635_s_at	0.1409	3.36E-02	-1.1306213	POLR2K	RNA polymerase II subunit K	5440
200886_s_at	0.1409	3.36E-02	-1.115288	PGAM1	phosphoglycerate mutase 1	5223
200775_s_at	0.1409	3.37E-02	-1.014072	HNRNPK	heterogeneous nuclear ribonucleoprotein K	3190
214665_s_at	0.1409	3.37E-02	-1.126022	CHP1	calcineurin like EF-hand protein 1	11261

207507_s_at	0.141	3.37E-02	-1.4264933	ATP5G3	ATP synthase, H+ transporting, mitochondrial Fo complex subunit C3 (subunit 9)	518
220526_s_at	0.141	3.38E-02	-1.129222	MRPL20	mitochondrial ribosomal protein L20	55052
200760_s_at	0.1411	3.38E-02	-1.0663553	ARL6IP5	ADP ribosylation factor like GTPase 6 interacting protein 5	10550
209835_x_at	0.1412	3.39E-02	-1.0304907	CD44	CD44 molecule (Indian blood group)	960
226436_at	0.1413	3.39E-02	-1.0178333	RASSF4	Ras association domain family member 4	83937
226635_at	0.1414	3.41E-02	-1.012724	EBLN3P	endogenous Bornavirus-like nucleoprotein 3, pseudogene	100506710

224692_at	0.1416	3.42E-02	-1.0457453	PPP1R15B	protein phosphatase 1 regulatory subunit 15B	84919
211072_x_at	0.1417	3.42E-02	-1.011126	TUBA1B	tubulin alpha 1b	10376
225708_at	0.1418	3.43E-02	-1.0040227	MED29	mediator complex subunit 29	55588
201398_s_at	0.1418	3.43E-02	-1.0111313	TRAM1	translocation associated membrane protein 1	23471
200934_at	0.1419	3.44E-02	-1.0344753	DEK	DEK proto-oncogene	7913
205005_s_at	0.1419	3.45E-02	-1.00385	NMT2	N-myristoyltransferase 2	9397
200876_s_at	0.142	3.45E-02	-1.229128	PSMB1	proteasome subunit beta 1	5689
207168_s_at	0.1421	3.46E-02	-1.0651827	H2AFY	H2A histone family member Y	9555
203791_at	0.1422	3.46E-02	-1.0937933	DMXL1	Dmx like 1	1657
210555_s_at	0.1422	3.46E-02	-1.1319267	NFATC3	nuclear factor of activated T-cells 3	4775

213699_s_at	0.1422	3.46E-02	-1.1238073	YWHAQ	tyrosine 3-monooxygenase/tryptophan 5-monooxygenase activation protein theta	10971
203983_at	0.1423	3.46E-02	-1.1070507	TSNAX	translin associated factor X	7257
202484_s_at	0.1423	3.46E-02	-1.0716987	MBD2	methyl-CpG binding domain protein 2	8932
204362_at	0.1423	3.47E-02	-1.023482	SKAP2	src kinase associated phosphoprotein 2	8935
202139_at	0.1424	3.47E-02	-1.0688767	AKR7A2	aldo-keto reductase family 7 member A2	8574
211747_s_at	0.1424	3.48E-02	-1.350304	LSM5	LSM5 homolog, U6 small nuclear RNA and mRNA degradation associated	23658

223244_s_at	0.1424	3.48E-02	-1.062198	NDUFA12	NADH:ubiquinone oxidoreductase subunit A12	55967
201892_s_at	0.1426	3.49E-02	-1.0285147	IMPDH2	inosine monophosphate dehydrogenase 2	3615
208980_s_at	0.1426	3.49E-02	-1.0556693	UBC	ubiquitin C	7316
200047_s_at	0.1426	3.49E-02	-1.2480427	YY1	YY1 transcription factor	7528
225356_at	0.1428	3.50E-02	-1.0353833	SFT2D2	SFT2 domain containing 2	375035
238778_at	0.1429	3.50E-02	-1.020836	MPP7	membrane palmitoylated protein 7	143098
217759_at	0.1429	3.51E-02	-1.012824	TRIM44	tripartite motif containing 44	54765
235593_at	0.1429	3.51E-02	-1.017726	ZEB2	zinc finger E-box binding homeobox 2	9839
206420_at	0.1431	3.52E-02	-1.445634	IGSF6	immunoglobulin superfamily member 6	10261
201110_s_at	0.1432	3.53E-02	-1.4341	THBS1	thrombospondin 1	7057

209312_x_at	0.1432	3.54E-02	-1.185634	LOC101060835///LOC100996809///HLA-DRB5///HLA-DRB4///HLA-DRB1///HLA-DQB1	HLA class II histocompatibility antigen, DQ beta 1 chain-like///HLA class II histocompatibility antigen, DRB1-10 beta chain-like///major histocompatibility complex, class II, DR beta 5///major histocompatibility complex, class II, DR beta 4///major	101060835///100996809///3127///3126///3123///3119
224791_at	0.1437	3.56E-02	-1.071488	ASAP1	ArfGAP with SH3 domain, ankyrin repeat and PH domain 1	50807
212250_at	0.1438	3.57E-02	-1.1706953	MTDH	metadherin	92140
203613_s_at	0.1439	3.57E-02	-1.1191393	NDUFB6	NADH:ubiquinone oxidoreductase subunit B6	4712
225368_at	0.1439	3.58E-02	-1.0345653	HIPK2	homeodomain interacting protein kinase 2	28996

208699_x_at	0.1439	3.58E-02	-1.132176	TKT	transketolase	7086
213911_s_at	0.1439	3.58E-02	-1.139568	H2AFZ	H2A histone family member Z	3015
222035_s_at	0.144	3.59E-02	-1.3929213	PAPOLA	poly(A) polymerase alpha	10914
227930_at	0.144	3.59E-02	-1.046202	AGO4	argonaute 4, RISC catalytic component	192670
218323_at	0.144	3.59E-02	-1.0790513	RHOT1	ras homolog family member T1	55288
212600_s_at	0.1441	3.60E-02	-1.08774	UQCRC2	ubiquinol-cytochrome c reductase core protein II	7385
201097_s_at	0.1441	3.60E-02	-1.2362413	ARF4	ADP ribosylation factor 4	378
225673_at	0.1442	3.61E-02	-1.180306	MYADM	myeloid associated differentiation marker	91663
219549_s_at	0.1443	3.61E-02	-1.1309673	RTN3	reticulon 3	10313
208805_at	0.1443	3.61E-02	-1.1158713	KIAA0391///PSMA6	KIAA0391///proteasome subunit alpha 6	9692///5687

203484_at	0.1444	3.62E-02	-1.1395193	SEC61G	Sec61 translocon gamma subunit	23480
221505_at	0.1445	3.63E-02	-1.1357167	ANP32E	acidic nuclear phosphoprotein 32 family member E	81611
1555837_s_at	0.1445	3.63E-02	-1.1480753	POLR2B	RNA polymerase II subunit B	5431
222235_s_at	0.1446	3.63E-02	-1.0797213	CSGALNACT2	chondroitin sulfate N-acetylgalactosaminyltransferase 2	55454
208766_s_at	0.1446	3.64E-02	-1.0056947	HNRNPR	heterogeneous nuclear ribonucleoprotein R	10236
211730_s_at	0.1446	3.64E-02	-1.167492	POLR2L	RNA polymerase II subunit L	5441
217947_at	0.1448	3.65E-02	-1.0631193	CMTM6	CKLF like MARVEL transmembrane domain containing 6	54918
219267_at	0.1449	3.66E-02	-1.0882173	GLTP	glycolipid transfer protein	51228

201132_at	0.1449	3.66E-02	-1.3874113	RPL36A-HNRNPH2///HNRNPH2	RPL36A-HNRNPH2 readthrough///heterogeneous nuclear ribonucleoprotein H2 (H')	100529097///3188
211967_at	0.1449	3.66E-02	-1.2811493	TMEM123	transmembrane protein 123	114908
202727_s_at	0.1449	3.66E-02	-1.08482	IFNGR1	interferon gamma receptor 1	3459
206790_s_at	0.1449	3.66E-02	-1.0839547	NDUFB1	NADH:ubiquinone oxidoreductase subunit B1	4707
211671_s_at	0.145	3.67E-02	-1.0384307	NR3C1	nuclear receptor subfamily 3 group C member 1	2908
200818_at	0.1451	3.67E-02	-1.1328613	ATP5O	ATP synthase, H+ transporting, mitochondrial F1 complex, O subunit	539
217811_at	0.1451	3.67E-02	-1.2766353	SELT	selenoprotein T	51714

210453_x_at	0.1451	3.67E-02	-1.1060887	ATP5L	ATP synthase, H+ transporting, mitochondrial Fo complex subunit G	10632
210980_s_at	0.1451	3.67E-02	-1.3416127	ASAH1	N-acylsphingosine amidohydrolase 1	427
217835_x_at	0.1452	3.69E-02	-1.097772	TGIF2-C20orf24///C20orf24	TGIF2-C20orf24 readthrough///chromosome 20 open reading frame 24	100527943///55969
202957_at	0.1452	3.69E-02	-1.0268927	HCLS1	hematopoietic cell-specific Lyn substrate 1	3059
213738_s_at	0.1453	3.70E-02	-1.101026	ATP5A1	ATP synthase, H+ transporting, mitochondrial F1 complex, alpha subunit 1, cardiac muscle	498
38241_at	0.1454	3.71E-02	-1.1029207	BTN3A3	butyrophilin subfamily 3 member A3	10384

225181_at	0.1454	3.71E-02	-1.0068467	ARID1B	AT-rich interaction domain 1B	57492
203401_at	0.1458	3.73E-02	-1.0445047	PRPS2	phosphoribosyl pyrophosphate synthetase 2	5634
209732_at	0.146	3.75E-02	-1.019972	CLEC2B	C-type lectin domain family 2 member B	9976
208248_x_at	0.1463	3.76E-02	-1.0094147	APLP2	amyloid beta precursor like protein 2	334
201004_at	0.1465	3.78E-02	-1.0039113	SSR4	signal sequence receptor subunit 4	6748
225283_at	0.1465	3.78E-02	-1.0747647	ARRDC4	arrestin domain containing 4	91947
225125_at	0.1466	3.78E-02	-1.1963147	MMGT1	membrane magnesium transporter 1	93380

208640_at	0.1466	3.78E-02	-1.2494213	RAC1	ras-related C3 botulinum toxin substrate 1 (rho family, small GTP binding protein Rac1)	5879
208918_s_at	0.1466	3.79E-02	-1.3051207	NADK	NAD kinase	65220
204971_at	0.1466	3.79E-02	-1.2072113	CSTA	cystatin A	1475
203140_at	0.1467	3.80E-02	-1.09964	BCL6	B-cell CLL/lymphoma 6	604
217773_s_at	0.1468	3.80E-02	-1.2189467	NDUFA4	NDUFA4, mitochondrial complex associated	4697
217741_s_at	0.1469	3.81E-02	-1.12023	ZFAND5	zinc finger AN1-type containing 5	7763
221493_at	0.1473	3.83E-02	-1.115768	TSPYL1	TSPY like 1	7259
200729_s_at	0.1473	3.84E-02	-1.2333293	ACTR2	ARP2 actin related protein 2 homolog	10097
202602_s_at	0.1473	3.84E-02	-1.02783	HTATSF1	HIV-1 Tat specific factor 1	27336
224769_at	0.1475	3.85E-02	-1.0446773	TAOK1	TAO kinase 1	57551
203885_at	0.1475	3.85E-02	-1.0314907	RAB21	RAB21, member RAS oncogene family	23011

39729_at	0.1476	3.86E-02	-1.2801653	PRDX2	peroxiredoxin 2	7001
224891_at	0.1476	3.86E-02	-1.0636567	FOXO3	forkhead box O3	2309
211985_s_at	0.1478	3.87E-02	-1.179536	CALM3///CALM2///CALM1	calmodulin 3///calmodulin 2///calmodulin 1	808///805///801
1554678_s_at	0.1479	3.87E-02	-1.3069053	HNRNPDL	heterogeneous nuclear ribonucleoprotein D like	9987
209142_s_at	0.1479	3.87E-02	-1.20178	UBE2G1	ubiquitin conjugating enzyme E2 G1	7326
204094_s_at	0.1479	3.88E-02	-1.1683313	TSC22D2	TSC22 domain family member 2	9819
201343_at	0.1485	3.91E-02	-1.0431453	UBE2D2	ubiquitin conjugating enzyme E2 D2	7322
212761_at	0.1485	3.91E-02	-1.239414	TCF7L2	transcription factor 7 like 2	6934
217764_s_at	0.1486	3.92E-02	-1.0575113	RAB31	RAB31, member RAS oncogene family	11031
201317_s_at	0.1486	3.92E-02	-1.148328	PSMA2	proteasome subunit alpha 2	5683

201017_at	0.1488	3.93E-02	-1.0519967	EIF1AX	eukaryotic translation initiation factor 1A, X-linked	1964
207785_s_at	0.1488	3.94E-02	-1.0419507	RBPJ	recombination signal binding protein for immunoglobulin kappa J region	3516
214181_x_at	0.1488	3.94E-02	-1.139324	LST1	leukocyte specific transcript 1	7940
218438_s_at	0.1488	3.94E-02	-1.020928	MED28	mediator complex subunit 28	80306
210629_x_at	0.1488	3.95E-02	-1.1416527	LST1	leukocyte specific transcript 1	7940
218213_s_at	0.1489	3.95E-02	-1.2651033	TMEM258	transmembrane protein 258	746
214149_s_at	0.1489	3.95E-02	-1.2121907	ATP6V0E1	ATPase H+ transporting V0 subunit e1	8992

209249_s_at	0.149	3.96E-02	-1.0745667	GHITM	growth hormone inducible transmembrane protein	27069
225795_at	0.149	3.96E-02	-1.335444	SMDT1	single-pass membrane protein with aspartate rich tail 1	91689
200693_at	0.1492	3.97E-02	-1.1862087	YWHAQ	tyrosine 3-monooxygenase/tryptophan 5-monooxygenase activation protein theta	10971
231259_s_at	0.1492	3.98E-02	1.086658	CCND2	cyclin D2	894
212857_x_at	0.1492	3.98E-02	-1.015336	SUB1	SUB1 homolog, transcriptional regulator	10923
209949_at	0.1492	3.98E-02	-1.2321953	NCF2	neutrophil cytosolic factor 2	4688
200065_s_at	0.1492	3.98E-02	-1.0780573	MIR3620///ARF1	microRNA 3620///ADP ribosylation factor 1	100500810///375

221666_s_at	0.1492	3.98E-02	-1.2170827	PYCARD	PYD and CARD domain containing	29108
225892_at	0.1492	3.98E-02	-1.164146	IREB2	iron responsive element binding protein 2	3658
226810_at	0.1494	3.99E-02	-1.3582667	OGFRL1	opioid growth factor receptor like 1	79627
201968_s_at	0.1496	4.00E-02	-1.0404573	PGM1	phosphoglucomutase 1	5236
209901_x_at	0.1497	4.01E-02	-1.0718	AIF1	allograft inflammatory factor 1	199
209189_at	0.15	4.05E-02	-1.189392	FOS	Fos proto-oncogene, AP-1 transcription factor subunit	2353
201299_s_at	0.15	4.05E-02	-1.0216913	MOB1A	MOB kinase activator 1A	55233
217761_at	0.1502	4.06E-02	-1.0188713	ADI1	acireductone dioxygenase 1	55256
222465_at	0.1503	4.07E-02	-1.2534933	RSL24D1	ribosomal L24 domain containing 1	51187
217398_x_at	0.1504	4.08E-02	-1.108006	GAPDH	glyceraldehyde-3-phosphate dehydrogenase	2597

215245_x_at	0.1505	4.08E-02	-1.2036253	FMR1	fragile X mental retardation 1	2332
221698_s_at	0.1506	4.09E-02	-1.0627933	CLEC7A	C-type lectin domain family 7 member A	64581
221482_s_at	0.1507	4.10E-02	-1.0901607	ARPP19	cAMP regulated phosphoprotein 19	10776
200798_x_at	0.1508	4.10E-02	-1.3600313	MCL1	BCL2 family apoptosis regulator	4170
201351_s_at	0.1508	4.10E-02	-1.2042053	YME1L1	YME1 like 1 ATPase	10730
227278_at	0.1509	4.12E-02	-1.134226	TAF13	TATA-box binding protein associated factor 13	6884
218351_at	0.151	4.12E-02	-1.053244	COMMD8	COMM domain containing 8	54951
205945_at	0.1515	4.15E-02	-1.139692	IL6R	interleukin 6 receptor	3570
227787_s_at	0.1515	4.15E-02	-1.0760587	MED30	mediator complex subunit 30	90390

211070_x_at	0.1516	4.16E-02	-1.1832967	DBI	diazepam binding inhibitor, acyl-CoA binding protein	1622
225351_at	0.1517	4.17E-02	-1.0058653	FAM45A///FAM45BP	family with sequence similarity 45 member A///family with sequence similarity 45, member A pseudogene	404636///55855
200902_at	0.1518	4.18E-02	-1.276888	15-Sep	15 kDa selenoprotein	9403
221532_s_at	0.1518	4.18E-02	-1.312664	WDR61	WD repeat domain 61	80349
201163_s_at	0.1518	4.18E-02	-1.254686	IGFBP7	insulin like growth factor binding protein 7	3490
200626_s_at	0.152	4.19E-02	-1.1206413	SNHG4///MATR3	small nucleolar RNA host gene 4///matrin 3	724102///9782
221553_at	0.1523	4.21E-02	-1.1374633	MAGT1	magnesium transporter 1	84061

207857_at	0.1523	4.22E-02	-1.32732	LILRA2	leukocyte immunoglobulin like receptor A2	11027
225921_at	0.1523	4.22E-02	-1.0120513	NIN	ninein	51199
203338_at	0.1523	4.22E-02	-1.054562	PPP2R5E	protein phosphatase 2 regulatory subunit B'epsilon	5529
1558972_s_at	0.1524	4.22E-02	-1.008572	THEMIS	thymocyte selection associated	387357
209970_x_at	0.1524	4.22E-02	-1.156366	CASP1	caspase 1	834
224899_s_at	0.1524	4.22E-02	-1.187902	MAGT1	magnesium transporter 1	84061
224584_at	0.1526	4.23E-02	-1.034364	TMEM230	transmembrane protein 230	29058
233011_at	0.1526	4.24E-02	-1.2981173	ANXA1	annexin A1	301
1553587_a_at	0.1526	4.24E-02	-1.0316573	POLE4	DNA polymerase epsilon 4, accessory subunit	56655
200608_s_at	0.1527	4.25E-02	-1.0230013	RAD21	RAD21 cohesin complex component	5885

200657_at	0.1528	4.26E-02	-1.0723193	SLC25A5	solute carrier family 25 member 5	292
209471_s_at	0.153	4.27E-02	-1.0374293	FNTA	farnesyltransferase, CAAX box, alpha	2339
218007_s_at	0.153	4.27E-02	-1.0399273	RPS27L	ribosomal protein S27 like	51065
209845_at	0.1531	4.28E-02	-1.0006633	MKRN1	makorin ring finger protein 1	23608
212820_at	0.1532	4.28E-02	-1.3647187	DMXL2	Dmx like 2	23312
202603_at	0.1532	4.29E-02	-1.045256	ADAM10	ADAM metallopeptidase domain 10	102
212867_at	0.1532	4.29E-02	-1.074466	NCOA2	nuclear receptor coactivator 2	10499
208894_at	0.1533	4.29E-02	-1.3869687	HLA-DRA	major histocompatibility complex, class II, DR alpha	3122
208692_at	0.1535	4.30E-02	-1.120388	RPS3	ribosomal protein S3	6188
207585_s_at	0.1537	4.32E-02	-1.012906	RPL36AL	ribosomal protein L36a like	6166
225579_at	0.1538	4.32E-02	-1.1608653	PQLC3	PQ loop repeat containing 3	130814

211609_x_at	0.1538	4.32E-02	-1.01422	PSMD4	proteasome 26S subunit, non-ATPase 4	5710
220990_s_at	0.1538	4.33E-02	-1.2343313	MIR21///VMP1	microRNA 21///vacuole membrane protein 1	406991///81671
211990_at	0.1538	4.33E-02	-1.0220167	HLA-DPA1	major histocompatibility complex, class II, DP alpha 1	3113
203799_at	0.154	4.33E-02	-1.4587073	LY75-CD302///CD302///LY75	LY75-CD302 readthrough///CD302 molecule///lymphocyte antigen 75	100526664///9936///4065
208891_at	0.154	4.34E-02	-1.4184213	DUSP6	dual specificity phosphatase 6	1848
213572_s_at	0.1541	4.34E-02	-1.280638	SERPINB1	serpin family B member 1	1992
221434_s_at	0.1543	4.35E-02	-1.1490173	SLIRP	SRA stem-loop interacting RNA binding protein	81892

210561_s_at	0.1544	4.36E-02	-1.0702793	WSB1	WD repeat and SOCS box containing 1	26118
211935_at	0.1545	4.37E-02	-1.096748	ARL6IP1	ADP ribosylation factor like GTPase 6 interacting protein 1	23204
208896_at	0.1546	4.37E-02	-1.0223913	DDX18	DEAD-box helicase 18	8886
218190_s_at	0.1548	4.38E-02	-1.0860067	UQCR10	ubiquinol-cytochrome c reductase, complex III subunit X	29796
226459_at	0.1549	4.39E-02	-1.0073227	PIK3AP1	phosphoinositide-3-kinase adaptor protein 1	118788
212774_at	0.155	4.40E-02	-1.0137827	ZBTB18	zinc finger and BTB domain containing 18	10472
201515_s_at	0.1551	4.41E-02	-1.0240247	TSN	translin	7247
217854_s_at	0.1552	4.41E-02	-1.0697807	POLR2E	RNA polymerase II subunit E	5434

234987_at	0.1552	4.41E-02	-1.493628	SAMHD1	SAM and HD domain containing deoxynucleoside triphosphate triphosphohydrolase 1	25939
214527_s_at	0.1553	4.42E-02	-1.0898827	PQBP1	polyglutamine binding protein 1	10084
205133_s_at	0.1554	4.43E-02	-1.0594753	HSPE1	heat shock protein family E (Hsp10) member 1	3336
213154_s_at	0.1554	4.43E-02	-1.0825093	BICD2	BICD cargo adaptor 2	23299
201463_s_at	0.1554	4.43E-02	-1.169766	TALDO1	transaldolase 1	6888
235542_at	0.1555	4.44E-02	-1.049088	TET3	tet methylcytosine dioxygenase 3	200424
212461_at	0.1556	4.44E-02	-1.2607787	AZIN1	antizyme inhibitor 1	51582
209330_s_at	0.1556	4.45E-02	-1.1776973	HNRNPD	heterogeneous nuclear ribonucleoprotein D	3184
226106_at	0.1557	4.45E-02	-1.1055873	RNF141	ring finger protein 141	50862

225512_at	0.1557	4.45E-02	-1.117608	ZBTB38	zinc finger and BTB domain containing 38	253461
235766_x_at	0.1557	4.45E-02	-1.0302113	RAB27A	RAB27A, member RAS oncogene family	5873
210592_s_at	0.1557	4.46E-02	-1.123066	SAT1	spermidine/spermine N1-acetyltransferase 1	6303
222990_at	0.1558	4.46E-02	-1.02781	UBQLN1	ubiquilin 1	29979
204174_at	0.1559	4.47E-02	-1.0101407	ALOX5AP	arachidonate 5-lipoxygenase activating protein	241
201724_s_at	0.1561	4.48E-02	-1.1364053	GALNT1	polypeptide N-acetylgalactosaminyltransferase 1	2589
1555756_a_at	0.1563	4.49E-02	-1.5771113	CLEC7A	C-type lectin domain family 7 member A	64581
217865_at	0.1563	4.50E-02	-1.232492	RNF130	ring finger protein 130	55819
204031_s_at	0.1563	4.50E-02	-1.037816	PCBP2	poly(rC) binding protein 2	5094

217883_at	0.1565	4.50E-02	-1.1674407	MMADHC	methylmalonic aciduria and homocystinuria, cblD type	27249
208761_s_at	0.1567	4.52E-02	-1.0357733	SUMO1	small ubiquitin-like modifier 1	7341
223042_s_at	0.1569	4.53E-02	-1.1823833	FUNDC2	FUN14 domain containing 2	65991
200022_at	0.1569	4.53E-02	-1.0418373	RPL18	ribosomal protein L18	6141
208739_x_at	0.1569	4.54E-02	-1.0854347	LOC101929087///SUMO2///SUMO3	small ubiquitin-related modifier 2 pseudogene///small ubiquitin-like modifier 2///small ubiquitin-like modifier 3	101929087///6613///6612
224702_at	0.1572	4.55E-02	-1.199796	TMEM167A	transmembrane protein 167A	153339
218700_s_at	0.1573	4.56E-02	-1.0350073	RAB29	RAB29, member RAS oncogene family	8934

217848_s_at	0.1573	4.56E-02	-1.1048947	PPA1	pyrophosphatase (inorganic) 1	5464
200762_at	0.1573	4.57E-02	-1.4224333	DPYSL2	dihydropyrimidinase like 2	1808
200036_s_at	0.1574	4.57E-02	-1.110996	RPL10A	ribosomal protein L10a	4736
201172_x_at	0.1575	4.58E-02	-1.0342587	ATP6V0E1	ATPase H+ transporting V0 subunit e1	8992
218226_s_at	0.1576	4.59E-02	-1.0553087	NDUFB4	NADH:ubiquinone oxidoreductase subunit B4	4710
223112_s_at	0.1576	4.59E-02	-1.1231487	NDUFB10	NADH:ubiquinone oxidoreductase subunit B10	4716
212501_at	0.1576	4.59E-02	-1.19584	CEBPB	CCAAT/enhancer binding protein beta	1051
229101_at	0.1577	4.60E-02	-1.0123687	IL17RA	interleukin 17 receptor A	23765
209459_s_at	0.1578	4.61E-02	-1.0172713	ABAT	4-aminobutyrate aminotransferase	18

202076_at	0.1579	4.61E-02	-1.0212387	BIRC2	baculoviral IAP repeat containing 2	329
223993_s_at	0.1579	4.62E-02	-1.049928	CNIH4	cornichon family AMPA receptor auxiliary protein 4	29097
212038_s_at	0.158	4.62E-02	-1.0004507	VDAC1	voltage dependent anion channel 1	7416
202797_at	0.158	4.63E-02	-1.0317787	SACM1L	SAC1 suppressor of actin mutations 1-like (yeast)	22908
201470_at	0.1584	4.65E-02	-1.0446433	GSTO1	glutathione S-transferase omega 1	9446
236198_at	0.1585	4.66E-02	-1.147718	LINC01215	long intergenic non-protein coding RNA 1215	101929623
200006_at	0.1586	4.66E-02	-1.0940713	PARK7	Parkinsonism associated deglycase	11315
214800_x_at	0.1587	4.67E-02	-1.0035207	BTF3	basic transcription factor 3	689
223592_s_at	0.1587	4.67E-02	-1.1644547	RNF135	ring finger protein 135	84282

218042_at	0.1591	4.69E-02	-1.0193267	COPS4	COP9 signalosome subunit 4	51138
200633_at	0.1592	4.70E-02	-1.0303793	UBB	ubiquitin B	7314
235446_at	0.1592	4.70E-02	1.62481	XIST	X inactive specific transcript (non-protein coding)	7503
200821_at	0.1593	4.71E-02	-1.353886	LAMP2	lysosomal associated membrane protein 2	3920
201923_at	0.1595	4.72E-02	-1.0013867	PRDX4	peroxiredoxin 4	10549
223516_s_at	0.1597	4.73E-02	-1.0556173	TOMM6///PRICKLE4	translocase of outer mitochondrial membrane 6///prickle planar cell polarity protein 4	100188893///29964
202090_s_at	0.1598	4.74E-02	-1.1033407	UQCR11	ubiquinol-cytochrome c reductase, complex III subunit XI	10975

223343_at	0.1599	4.75E-02	-1.422256	MS4A7	membrane spanning 4-domains A7	58475
230543_at	0.1599	4.75E-02	1.1726493	USP9X	ubiquitin specific peptidase 9, X-linked	8239
221452_s_at	0.1599	4.75E-02	-1.3327993	TMEM14B	transmembrane protein 14B	81853
212476_at	0.16	4.75E-02	-1.165268	ACAP2	ArfGAP with coiled-coil, ankyrin repeat and PH domains 2	23527
210754_s_at	0.16	4.76E-02	-1.250076	LYN	LYN proto-oncogene, Src family tyrosine kinase	4067
210024_s_at	0.16	4.76E-02	-1.123678	UBE2E3	ubiquitin conjugating enzyme E2 E3	10477
224321_at	0.1601	4.77E-02	1.1317107	TMEFF2	transmembrane protein with EGF like and two follistatin like domains 2	23671

217933_s_at	0.1605	4.79E-02	-1.222424	LAP3	leucine aminopeptidase 3	51056
225798_at	0.1608	4.80E-02	-1.1661607	JAZF1	JAZF zinc finger 1	221895
213537_at	0.1609	4.81E-02	-1.094638	HLA-DPA1	major histocompatibility complex, class II, DP alpha 1	3113
209248_at	0.1609	4.81E-02	-1.12027	GHITM	growth hormone inducible transmembrane protein	27069
222473_s_at	0.1612	4.83E-02	-1.0348133	ERBIN	erbb2 interacting protein	55914
224989_at	0.1612	4.83E-02	-1.117988	SMIM14	small integral membrane protein 14	201895
201568_at	0.1614	4.84E-02	-1.0595027	UQCRQ	ubiquinol-cytochrome c reductase complex III subunit VII	27089
222430_s_at	0.1616	4.86E-02	-1.0348407	YTHDF2	YTH N6-methyladenosine RNA binding protein 2	51441
201426_s_at	0.1621	4.90E-02	-1.0043353	VIM	vimentin	7431

224604_at	0.1622	4.91E-02	-1.4050973	C4orf3	chromosome 4 open reading frame 3	401152
218611_at	0.1622	4.91E-02	-1.1703393	IER5	immediate early response 5	51278
213702_x_at	0.1623	4.93E-02	-1.26028	ASAH1	N-acylsphingosine amidohydrolase 1	427
200718_s_at	0.1624	4.93E-02	-1.0032807	SKP1	S-phase kinase-associated protein 1	6500
201273_s_at	0.1624	4.93E-02	-1.0930227	SRP9	signal recognition particle 9	6726
218499_at	0.1624	4.93E-02	-1.0429953	STK26	serine/threonine protein kinase 26	51765
213241_at	0.1624	4.94E-02	-1.173842	PLXNC1	plexin C1	10154
203535_at	0.1625	4.94E-02	-1.263982	S100A9	S100 calcium binding protein A9	6280
201630_s_at	0.1628	4.96E-02	-1.005488	ACP1	acid phosphatase 1, soluble	52
200871_s_at	0.163	4.98E-02	-1.177606	PSAP	prosaposin	5660
201858_s_at	0.1632	4.99E-02	-1.208804	SRGN	serglycin	5552
