# Supplementary material for: Virtual Screening of Cablin Patchouli Herb as a Treatment for Heat Stress: A Study Based on Network Pharmacology, Molecular Docking, and Experimental Verification
Source: Evid Based Complement Alternat Med. 2021 Mar 10;2021:8057587. doi: 10.1155/2021/8057587 (PMC7969090; doi:10.1155/2021/8057587)
Supplement: Supplementary Materials — Supplementary Table 1: 1789 heat stress differentially expressed genes. Supplementary Table 2: the CPB components and targets after correction. [file 8057587.f1.zip › 8057587.f1/Supplementary table 2.pdf]

MolName	TargetName	GeneSymbol
quercetin	26S proteasome non-ATPase regulatory subunit 3	PSMD3
quercetin	Acetyl-CoA carboxylase 1	ACACA
quercetin	Acetylcholinesterase	ACHE
irisolidone	Acetylcholinesterase	ACHE
3,23-dihydroxy-12-oleanen-28-oic acid	Acetylcholinesterase	ACHE
quercetin	Activator of 90 kDa heat shock protein ATPase homolog 1	AHSA1
3,23-dihydroxy-12-oleanen-28-oic acid	Androgen receptor	AR
Genkwanin	Androgen receptor	AR
irisolidone	Androgen receptor	AR
quercetin	Androgen receptor	AR
phenanthrone	Androgen receptor	AR
quercetin 7-O-β-D-glucoside	Androgen receptor	AR
quercetin	Apoptosis regulator Bcl-2	BCL2
quercetin	Arachidonate 5-lipoxygenase	ALOX5
quercetin	Aryl hydrocarbon receptor	AHR
quercetin	Baculoviral IAP repeat-containing protein 5	BIRC5
quercetin	C-reactive protein	CRP
quercetin	Caspase-3	CASP3
quercetin	Caspase-8	CASP8
quercetin	Caspase-9	CASP9
quercetin	Cathepsin D	CTSD
quercetin	Caveolin-1	CAV1
quercetin	Caveolin-1	CAV1 CAV
quercetin	Cellular tumor antigen p53	TP53
quercetin	Cellular tumor antigen p53	TP63
quercetin	Cellular tumor antigen p53	TP73 hCG_19088
quercetin	Coagulation factor VII	F7
quercetin	Collagen alpha-1(III) chain	COL3A1
quercetin	Cytochrome P450 1A1	CYP1A1
quercetin	Cytochrome P450 1B1	CYP1B1
quercetin	Cytochrome P450 3A4	CYP3A4
quercetin	DDB1- and CUL4-associated factor 5	DCAF5
quercetin	Dual oxidase 2	DUOX2
quercetin	E-selectin	SELE
quercetin	Epidermal growth factor receptor	EGFR
irisolidone	Estrogen receptor	ESR1
3,23-dihydroxy-12-oleanen-28-oic acid	Estrogen receptor	ESR1
quercetin 7-O-β-D-glucoside	Estrogen receptor	ESR1
Genkwanin	Estrogen receptor beta	ESR2
irisolidone	Estrogen receptor beta	ESR2
3,23-dihydroxy-12-oleanen-28-oic acid	Estrogen receptor beta	ESR2

quercetin	ETS domain-containing protein Elk-1	ELK1
quercetin	Eukaryotic translation initiation factor 6	EIF6
quercetin	G1/S-specific cyclin-D1	CCND1
quercetin	G2/mitotic-specific cyclin-B1	CCNB1 CCNB
quercetin	Gamma-aminobutyric acid receptor subunit alpha-1	GABRA1
phenanthrone	Gamma-aminobutyric acid receptor subunit alpha-1	GABRA1
quercetin	Glutathione S-transferase Mu 1	GSTM1
quercetin	Glutathione S-transferase Mu 2	GSTM2
quercetin	Glutathione S-transferase P	GSTP1
irisolidone	Glycogen synthase kinase-3 beta	GSK3B
3,23-dihydroxy-12-oleanen-28-oic acid	Glycogen synthase kinase-3 beta	GSK3B
quercetin	Heat shock factor protein 1	HSF1
quercetin	Heat shock protein beta-1	HSPB1
quercetin	Hexokinase-2	HK2
quercetin	Hypoxia-inducible factor 1-alpha	HIF1A
quercetin	Insulin-like growth factor-binding protein 3	IGFBP3
quercetin	Intercellular adhesion molecule 1	ICAM1
quercetin	Interferon regulatory factor 1	IRF1
quercetin	Interleukin-10	IL10
quercetin	Interleukin-6	IL6
quercetin	Maltase-glucoamylase, intestinal	MGAM
phenanthrone	Muscarinic acetylcholine receptor M1	CHRM1
Diop	Muscarinic acetylcholine receptor M3	CHRM3
phenanthrone	Muscarinic acetylcholine receptor M3	CHRM3
quercetin	Myc proto-oncogene protein	MYC
quercetin	Myc proto-oncogene protein	MYC hCG_15917
quercetin	NAD(P)H dehydrogenase [quinone] 1	NQO1
quercetin	NADPH--cytochrome P450 reductase	POR
quercetin	NF-kappa-B inhibitor alpha	NFKBIA

quercetin	Nitric oxide synthase, endothelial	NOS3
quercetin	Nuclear factor erythroid 2-related factor 2	NFE2L2
irisolidone	Nuclear receptor coactivator 1	NCOA1
3,23-dihydroxy-12-oleanen-28-oic acid	Nuclear receptor coactivator 1	NCOA1
Genkwanin	Nuclear receptor coactivator 1	NCOA1
Genkwanin	Nuclear receptor coactivator 2	NCOA2
irisolidone	Nuclear receptor coactivator 2	NCOA2
quercetin	Nuclear receptor coactivator 2	NCOA2
quercetin 7-O-β-D-glucoside	Nuclear receptor coactivator 2	NCOA2
3,23-dihydroxy-12-oleanen-28-oic acid	Nuclear receptor coactivator 2	NCOA2
quercetin	Nuclear receptor subfamily 1 group I member 3	NR1I3
quercetin	Peroxisome proliferator-activated receptor gamma	PPARG
quercetin	Poly [ADP-ribose] polymerase 1	PARP1
quercetin	Pro-epidermal growth factor	EGF
quercetin	Prostaglandin E2 receptor EP3 subtype	PTGER3
5-Hydroxy-7,4'-dimethoxyflavanon	Prostaglandin G/H synthase 1	PTGS1
irisolidone	Prostaglandin G/H synthase 1	PTGS1
quercetin 7-O-β-D-glucoside	Prostaglandin G/H synthase 1	PTGS1
phenanthrone	Prostaglandin G/H synthase 1	PTGS1
Genkwanin	Prostaglandin G/H synthase 1	PTGS1
3,23-dihydroxy-12-oleanen-28-oic acid	Prostaglandin G/H synthase 1	PTGS1
quercetin	Prostaglandin G/H synthase 1	PTGS1
quercetin	Protein CBFA2T1	RUNX1T1
quercetin	Protein kinase C alpha type	PRKCA
quercetin	Proto-oncogene c-Fos	FOS
quercetin	Puromycin-sensitive aminopeptidase	NPEPPS
quercetin	RAF proto-oncogene serine/threonine-protein kinase	RAF1
quercetin	Ras association domain-containing protein 1	RASSF1 RDA32
quercetin	Ras GTPase-activating protein 1	RASA1
quercetin	Receptor tyrosine-protein kinase erbB-2	ERBB2
quercetin	Receptor tyrosine-protein kinase erbB-3	ERBB3
quercetin	Retinoblastoma-associated protein	RB1
phenanthrone	Retinoic acid receptor RXR-alpha	RXRA

quercetin	Retinoic acid receptor RXR-alpha	RXRA
5-Hydroxy-7,4'-dimethoxyflavanon	Retinoic acid receptor RXR-alpha	RXRA
irisolidone	Retinoic acid receptor RXR-alpha	RXRA
Genkwanin	Retinoic acid receptor RXR-alpha	RXRA
quercetin	Runt-related transcription factor 2	RUNX2
Genkwanin	Serine/threonine-protein kinase Chk1	CHEK1
irisolidone	Serine/threonine-protein kinase Chk1	CHEK1
3,23-dihydroxy-12-oleanen-28-oic acid	Serine/threonine-protein kinase Chk1	CHEK1
quercetin	Serine/threonine-protein kinase Chk2	CHEK2
quercetin	Serum paraoxonase/arylesterase 1	PON1
quercetin	Solute carrier family 2, facilitated glucose transporter member 4	SLC2A4
quercetin	Stromelysin-1	MMP3
quercetin	Transcription factor p65	RELA
irisolidone	Transcription factor p65	RELA
quercetin	Trypsin-1	PRSS1
Genkwanin	Trypsin-1	PRSS1
irisolidone	Trypsin-1	PRSS1
3,23-dihydroxy-12-oleanen-28-oic acid	Trypsin-1	PRSS1
quercetin	Type I iodothyronine deiodinase	DIO1
quercetin	Urokinase-type plasminogen activator	PLAU
quercetin	Vascular cell adhesion protein 1	VCAM1
quercetin	Vascular endothelial growth factor A	VEGFA
